# Comparing efficacy and safety in catheter ablation strategies for atrial fibrillation: a network meta-analysis

**DOI:** 10.1186/s12916-022-02385-2

**Published:** 2022-05-31

**Authors:** Emmanouil Charitakis, Silvia Metelli, Lars O. Karlsson, Antonios P. Antoniadis, Konstantinos D. Rizas, Ioan Liuba, Henrik Almroth, Anders Hassel Jönsson, Jonas Schwieler, Dimitrios Tsartsalis, Skevos Sideris, Elena Dragioti, Nikolaos Fragakis, Anna Chaimani

**Affiliations:** 1 Department of Cardiology and Department of Health, Medicine and Caring Sciences, Linköping University, Linköping, Sweden; 2 Research Center of Epidemiology and Statistics (CRESS-U1153), Université Paris Cité, INSERM, Paris, France; 3 3rd Cardiology Department, Hippokrateion General Hospital, Aristotle University Medical School, Thessaloniki, Greece; 4 Medizinische Klinik Und Poliklinik I, LMU Klinikum, Ludwig-Maximilians-University Munich, Munich, Germany; 5 Heart and Vascular Theme, Karolinska University Hospital, Stockholm, Sweden; 6 Department of Emergency Medicine, Hippokration Hospital, Athens, Greece; 7 Department of Cardiology, National and Kapodistrian University of Athens, Hippokration Hospital, Athens, Greece; 8 Pain and Rehabilitation Centre and Department of Health, Medicine and Caring Sciences, Linköping University, Linköping, Sweden

**Keywords:** Network meta-analysis, Atrial fibrillation, Catheter ablation, Efficacy, Safety, Antiarrhythmic drugs

## Abstract

**Background:**

There is no consensus on the most efficient catheter ablation (CA) strategy for patients with atrial fibrillation (AF). The objective of this study was to compare the efficacy and safety of different CA strategies for AF ablation through network meta-analysis (NMA).

**Methods:**

A systematic search of PubMed, Web of Science, and CENTRAL was performed up to October 5th, 2020. Randomized controlled trials (RCT) comparing different CA approaches were included. Efficacy was defined as arrhythmia recurrence after CA and safety as any reported complication related to the procedure during a minimum follow-up time of 6 months.

**Results:**

In total, 67 RCTs (*n* = 9871) comparing 19 different CA strategies were included. The risk of recurrence was significantly decreased compared to pulmonary vein isolation (PVI) alone for PVI with renal denervation (RR: 0.60, CI: 0.38–0.94), PVI with ganglia-plexi ablation (RR: 0.62, CI: 0.41–0.94), PVI with additional ablation lines (RR: 0.8, CI: 0.68–0.95) and PVI in combination with bi-atrial modification (RR: 0.32, CI: 0.11–0.88). Strategies including PVI appeared superior to non-PVI strategies such as electrogram-based approaches. No significant differences in safety were observed.

**Conclusions:**

This NMA showed that PVI in combination with additional CA strategies, such as autonomic modulation and additional lines, seem to increase the efficacy of PVI alone. These strategies can be considered in treating patients with AF, since, additionally, no differences in safety were observed. This study provides decision-makers with comprehensive and comparative evidence about the efficacy and safety of different CA strategies.

**Systematic review registration:**

PROSPERO registry number: CRD42020169494.

**Supplementary Information:**

The online version contains supplementary material available at 10.1186/s12916-022-02385-2.

## Background

The main goal of treatment for atrial fibrillation (AF) is to treat symptoms and/or arrhythmia-induced heart failure. Primarily this involves pharmacological treatment and optimization of comorbidity, followed by antiarrhythmic treatment [1].

Cather ablation (CA) of AF became a treatment option after Haissaguerre’s seminal study, where ectopic areas adjacent to the pulmonary veins were found to initiate AF and thus objectified an ablation target for the treatment of AF [2, 3]. Since then, ablation procedures for AF have become an important treatment option and the number of interventions is increasing worldwide.

The clinical problem is that the AF population is heterogeneous, and pulmonary vein isolation (PVI) alone is not the solution for all patients. As a result, different approaches or CA strategies have been suggested, but robust data are scarce, and randomized direct comparisons are rare [3, 4].

Commonly used CA strategies include linear lesions, left atrial (LA) posterior wall isolation, substrate modification, electrocardiogram (EGM)-based approaches, along with ablation of trigger sites and ganglia-plexi (GP), mainly as add-ons to PVI [3]. Yet, the efficacy of different CA ablation strategies as stand-alone or add-on to PVI has been ambiguous [4].

The objective of this study was to systematically review the efficacy and safety of all different CA strategies for the treatment of patients with paroxysmal (PAF) and non-paroxysmal AF (non-PAF). To assess treatments that have not been directly compared in previous trials, we employed a network meta-analysis (NMA). NMA is a statistical method that enables the possibility to evaluate multiple treatments in a single analysis, combining not only direct but also indirect comparisons of treatments.

Compared to a conventional meta-analysis that is limited to evaluate two interventions at a time and only compares interventions evaluated directly in head-to-head trials, the NMA provides the possibility to evaluate multiple treatments in a single analysis. This is possible by combining direct and indirect evidence (i.e., comparisons can be made even if two strategies have not been directly compared in a single study) [4, 5]. NMA is robust and has already been applied in several medical fields [6] supporting guidelines and decision-making at different levels [7].

## Methods

### Study design and registration

This study follows the Preferred Reporting Items for Systematic Reviews and Meta-analyses (PRISMA) Extension Statement for NMA [8] (Additional file 1). This NMA is based on previously published data, thus it does not require ethical approval or consent to participate. The study protocol was registered on PROSPERO (registration number CRD42020169494) and has been published previously [9].

### Eligibility criteria and type of interventions

Randomized controlled trials (RCTs) that included men and women with PAF and non-PAF (2) were eligible for inclusion. RCTs that included patients with prior ablation (catheter, surgical or atrioventricular node ablation) were non-eligible.

The primary interventions of interest included CA strategies. In addition to PVI, non-PVI strategies, along with different strategies complementary to PVI, were evaluated. Table 1 contains the full list of interventions included alongside the abbreviations used.

**Table 1:** Interventions included in NMA and their abbreviations

Interventions included in the NMA	Abbreviations of interventions included in NMA
Electrocardiogram based ablation	EGM
Ganglia plexi ablation	GP
Non-PVI lines ablation	lines
Pulmonary vein isolation	PVI
PVI and bi-atrial modification	PVI + BI-mod
PVI and combination of additional lines ablation and electrocardiogram-based ablation	PVI + comb
PVI and electrocardiogram-based ablation	PVI + EGM
PVI and ganglia plexi ablation	PVI + GP
PVI and left atrial auricle isolation	PVI + LAA
PVI and additional lines ablation	PVI + lines
PVI, posterior box isolation ± additional lines ablation	PVI + posterior ± lines
PVI and renal denervation	PVI + RDN
PVI and substrate modification	PVI + SUB-mod
PVI, superior vena cava isolation ± additional line ablation	PVI + SVC ± lines
PVI and stepwise approach	PVI + step
Isolation of some pulmonary veins	PVI partly
PVI and trigger ablation	PVI + trig
Single box isolation	Single box
Single box isolation and additional lines	Single box + lines
Antiarrhythmic drugs	AAD (used only in a sensitivity analysis)

### Search strategy and study selection

A comprehensive search to identify relevant studies was performed by two investigators (EC and DT) using PubMed, the Cochrane Central Register of Controlled Trials, and the Web of Science. The search code is available in Additional file 2. The reference lists of included studies and previously published systematic reviews were searched to identify additional studies. The final search date was October 5th, 2020.

Two investigators (EC and DT) reviewed identified titles and abstracts independently, after which complete texts of eligible articles were obtained. Any disagreement between the reviewers was resolved by discussion with a third member of the investigator team (ED).

### Data items and data collection

Two investigators (EC and DT) read each article and performed data abstraction independently. Any disagreements were resolved by consultation with a third reviewer (ED) [9].

Data on study characteristics were summarized (e.g., first author, publication year, trial design), patient characteristics (age, sex, type of AF, etc.), intervention-related data (fluoroscopy time, blanking period, time for follow-up, the method used for the detection of AF, etc.) and outcome measures. If articles were lacking data, the original authors were contacted for supplementation requests.

### Outcomes

#### Primary outcomes

##### Efficacy

Recurrence of AF or/and atrial tachycardia (AT) with a duration of ≥ 30 s recorded on implantable loop recorder, pacemaker, defibrillator, ECG, or ambulatory-ECG during a minimum follow-up of 6 months after CA.

##### Safety

Any reported complications related to the procedure (periprocedural or occurring during the follow-up).

#### Secondary outcomes

*All-cause mortality* was evaluated from randomization and study start to the end of follow-up.

*Procedural time* was defined as the time from vessel puncture to end of the procedure.

### Quality assessment

The Cochrane Collaboration Risk of Bias (RoB) tool for randomized trials (RoB V.2) was used to rate the quality of the included RCTs [10]. When supplementation was not possible, the impact of missing outcome data was assessed using RoB (Additional file 3) [9, 10].

### Statistical analysis and evaluation of assumptions

The fundamental assumption of transitivity (i.e., the assumption that the relative effect between two treatments can be inferred via one or more intermediate comparators) was evaluated by comparing the distribution of potential effect modifiers across the different direct comparisons in the data [11, 12].

We estimated summary risk ratios (RRs) for efficacy and safety and mean differences (MDs) for procedural time using random-effects pairwise, and network meta-analysis and the graph-theoretical approach to NMA implemented in the R package “netmeta” [13, 14]. A network diagram for each outcome was created to present the structure of the data. Finally, forest plots and league tables presenting relative effect estimates between all included strategies for the outcomes were modeled. As many treatments included in the analyses were combinations of two or more strategies, we additionally performed a component network meta-analysis (CNMA), an extension of standard NMA that allowed deriving estimates of the effects of singular treatments, even when they have been used in combination with others [15, 16]. Specifically, to assess the individual effect of each treatment component we used an additive CNMA model in which the effect of a treatment combination is modeled as the sum of the individual treatments. Finally, we ranked the treatments for the primary outcomes using P-scores, which provide an *average degree of certainty* for a treatment to be better than the other interventions in the network [17].

Statistical heterogeneity was assessed considering the magnitude of the between-study variance (${\tau }^{2}$) [18]. In the NMA, the amount of heterogeneity was assumed to be the same across treatment comparisons.

Statistical incoherence (i.e., the disagreement of direct and indirect evidence) was assessed using the side-splitting method [19] and the design-by-treatment interaction model [20]. The former tests incoherence for every comparison while the latter is a global test for the entire network.

We used comparison-adjusted funnel plots for all active strategies against control (PVI) to graphically evaluate the presence of small-study effects [5, 21], namely whether results in imprecise studies differ from those in more precise studies.

Potential sources of heterogeneity and incoherence were evaluated through subgroup analyses and network meta regressions. Subgroup analyses depended on AF detection device, re-ablation, and antiarrhythmic drugs (AADs) allowance during the follow-up, follow-up duration, type of AF included in the original studies (PAF, non-PAF, or mixed), duration of the blanking period, and year of publication. For network meta-regression we used age, sex, publication year, hypertension, structural heart disease or coronary artery disease, left atrial dimensions, duration of follow-up, and AF detection device as covariates, analyzing only those variables with non-missing values for at least 10 studies. For primary outcomes, we ran sensitivity analyses by omitting studies with high RoB, studies involving renal denervation, studies involving “only PAF patients” and the use of non-irrigational RFA-catheters. Finally, we conducted a sensitivity analysis including studies with AADs as a comparing arm in the network, and to sum up our results in fewer categories, we performed a sensitivity analysis summing the 19 categories into 6 larger ones (more details can be found in Additional files 4, 5, 6, 7, 8, 9, 10, 11, 12, 13).

### Credibility of the evidence

Regarding the primary outcomes, we evaluated the overall credibility of the evidence in the network using the Confidence in Network-Meta Analysis (CINeMA) tool [22]. CINeMA allows to assess and summarize the level of concern for each comparison based on the contributions of the direct comparisons to the NMA estimation (Additional file 14, Tables S1-S2).

## Results

### Characteristics and risk of bias of the included studies

**Fig. 1:**
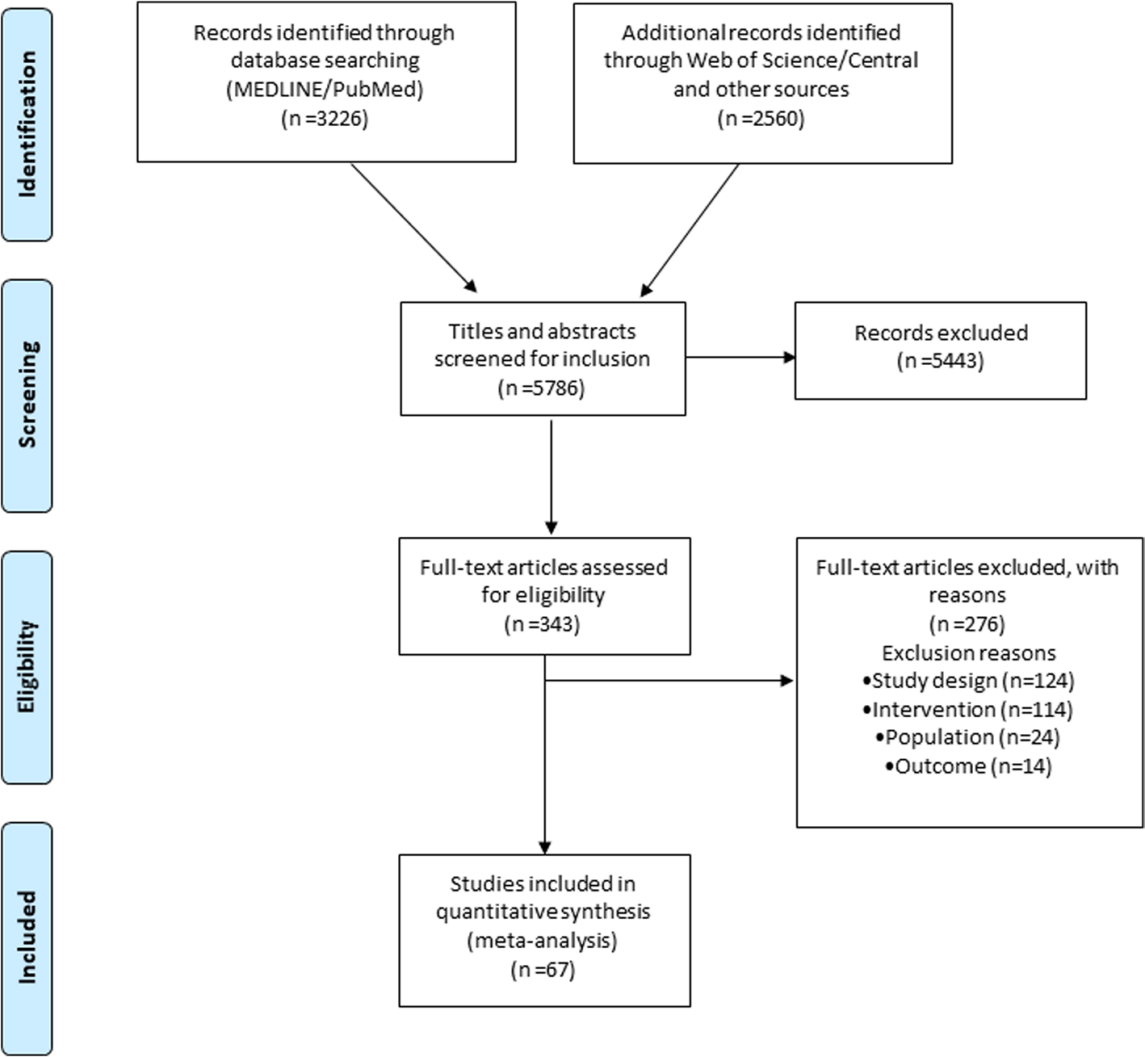
PRISMA flow chart diagram

The literature search yielded 5786 articles of which 343 were potentially eligible. Overall, 67 RCTs including 9871 patients between 2003 and 2020 (*n* = 9871) comparing 19 different CA strategies were included (Fig. 1) (59 two-arm, 7 three-arm, and 1 four-arm studies). The mean age was 58 ± 3 years and the mean proportion of males was 73 ± 9%. Twenty-seven RCTs (40%) included patients with only PAF, 23 (34%) only non-PAF and 17 (26%) had a mixed population (Additional file 4, Table S1) [4, 23–88].

The secondary endpoint “all-cause mortality” was not analyzed due to the large number of studies with zero events in both arms. All deviations from the original protocol are presented in the Additional file 5 in the Supplement.

Eleven (16%) RCTs included in this NMA revealed a high risk of bias when assessed by RoB V2-tool, whereas the remaining raised some concerns (Additional file 3, Table S1). The blinding of the care providers was not feasible due to the nature of the compared interventions.

### Network geometries and transitivity

**Fig. 2:**
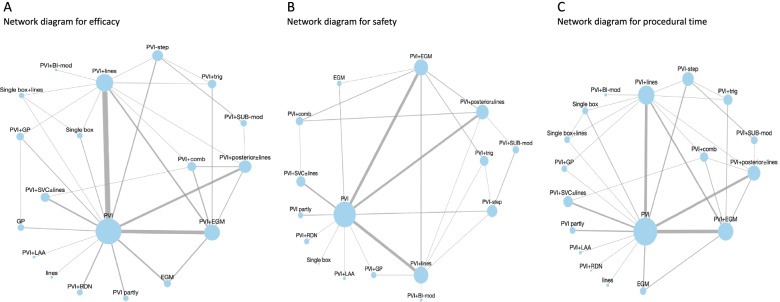
Network plots for efficacy (**A**), safety (**B**), and procedural time (**C**). Each treatment is represented as a node and an edge exists between two nodes if direct trial evidence is available. The size of each node is proportional to the number of patients involved in each treatment across all trials, while the size of the edges is proportional to the number of studies available in the corresponding comparison. Abbreviations: Bi, bi-atrial; comb, combination; EGM, electrocardiogram; GP, ganglia plexi; mod. modification; LAA, left atrial appendage; PVI, pulmonary vein isolation; RDN, renal denervation; RR, risk ratio; step, stepwise ablation; sub, substrate; SVC, superior vena cava; trig, trigger

The network diagrams of available comparisons for each outcome are presented in Fig. 2. PVI in combination with lines (PVI + lines) vs PVI and PVI in combination with EGM (PVI + EGM) vs PVI were the most prevalent. Efficacy was reported in all studies, safety in 58 (86.5%) and procedural time in 57 (85.1%). For efficacy, at least one direct comparison was available for each strategy. For safety, no direct evidence was present for ablation lines nor PVI in combination with renal denervation (PVI + RDN), while for procedural time, no direct evidence involved GP ablation.

Studies including AADs were excluded from the main analysis due to transitivity issues. No important clinical differences in the distributions of most effect modifiers were observed (Additional file 6) in the remaining network. The transitivity assumption could not be properly evaluated for structural heart disease (SHD) and coronary artery disease (CAD) due to the small number of available data.

### Relative effects and ranking of strategies

**Fig. 3:**
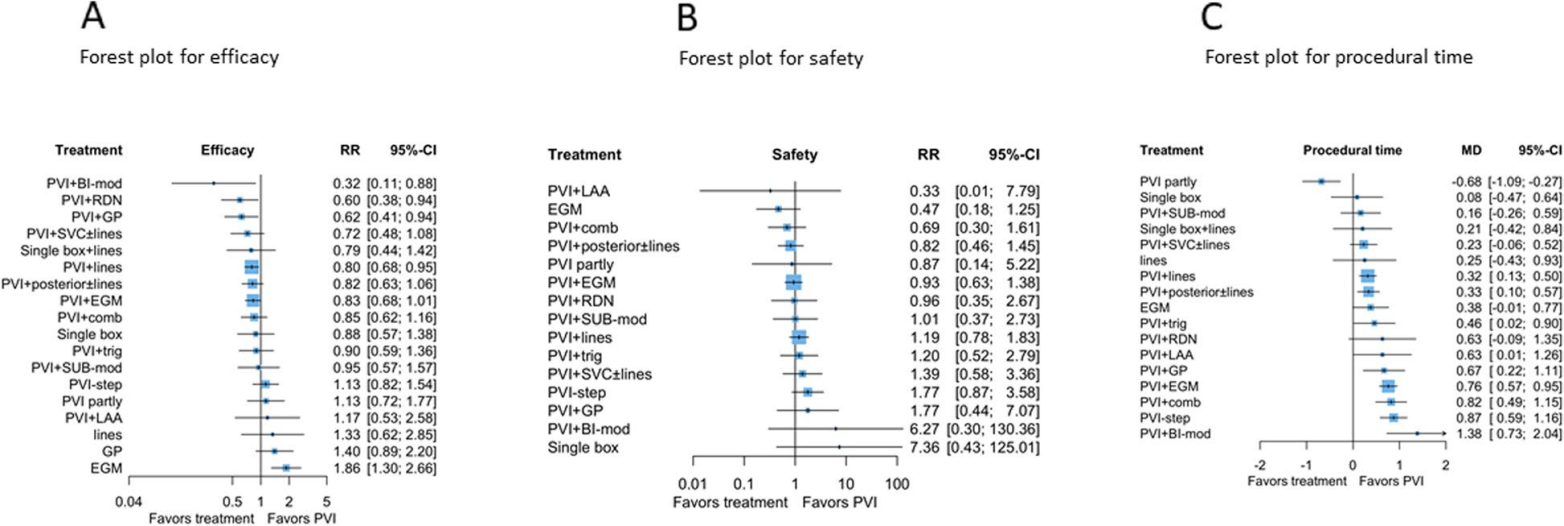
Forest plots for efficacy (**A**), safety (**B**), and procedural time (**C**) compared with PVI showing the network meta-analysis RRs with their 95% CIs. Abbreviations: Bi, bi-atrial; comb, combination; EGM, electrocardiogram; GP, ganglia plexi; mod, modification; LAA, left atrial appendage; PVI, pulmonary vein isolation; RDN, renal denervation; RR, risk ratio; step, stepwise ablation; sub, substrate; SVC, superior vena cava; trig, trigger

**Fig. 4:**
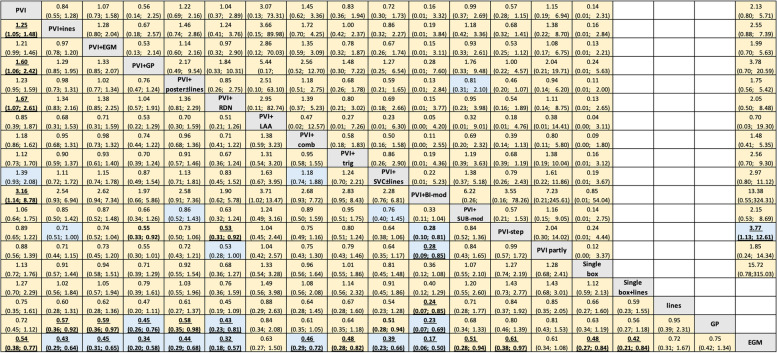
RRs for efficacy (lower triangle) and safety (upper triangle) with their 95% CIs derived from network meta-analysis of 19 AF strategies in the full network colored by certainty of evidence assessed for each comparison with CINeMA and classified in high (in green), moderate (in blue), low (in yellow) and very low (in red). Empty cells correspond to comparisons not available for the safety outcome. RRs lower than 1 favor the treatment in the column for both outcomes. Abbreviations: Bi, bi-atrial; comb, combination; EGM, electrocardiogram; GP, ganglia plexi; mod, modification; LAA, left atrial appendage; RDN, renal denervation; RR, risk ratio; step, stepwise ablation; sub, substrate; SVC, superior vena cava; trig, trigger

According to the NMA results, the risk for arrhythmia recurrence was significantly decreased for PVI in combination with biatrial modification (PVI + BI-mod) (RR: 0.31, CI: 0.11–0.88), PVI + RDN (RR: 0.60, CI: 0.38–0.94), PVI + GP (RR: 0.62, CI: 0.41–0.94) and PVI + lines (RR: 0.8, CI: 0.68–0.95) in comparison with PVI alone (Fig. 3a and Additional file 7 Table S1). However, PVI proved to be superior to an EGM-derived approach (RR: 1.86, CI 1.30–2.66). Interestingly, there were no significant differences in the efficacy between common CA-strategies, such as PVI + lines or PVI with posterior box (PVI + posterior ± lines) and PVI + EGM (Fig. 4). Moreover, non-PVI ablation strategies, such as EGM ablation as stand-alone strategies, had significantly lower efficacy compared to most other CA strategies.

Regarding safety, no significant difference between different CA-strategies and CA-strategies compared to PVI (Fig. 3b and Additional file 7 Table S2) was evident. PVI in combination with left atrial appendage isolation (PVI + LAA), PVI + EGM, PVI + posterior ± lines, and isolation of some pulmonary veins (PVI partly) appeared to have a lower risk of complications, but overall uncertainty was large, given their wide Cis. This finding might be partially explained by the limited statistical power of the model.

Only “PVI partly” was found to have significantly lower procedural time than PVI (MD: − 0.68, CI: − 1.09, − 0.27), while PVI + GP, PVI + EGM, PVI + BI-mod, PVI with a stepwise approach (PVI + step), PVI + lines and PVI + EGM, was more time consuming when compared with stand-alone PVI (Fig. 3c and Additional file 7 Table S3).

According to the *P*-scores, the highest-ranked treatments for efficacy were PVI in combination with adjuvant ablation (Additional file 7, Fig. S1).

When an additive CNMA model was employed (Additional file 8, Figs. S1-4), very small differences concerning the standard NMA model were identified, suggesting that no specific singular component is driving the total effect of treatments used in combination. Full results and further explanation of the CNMA model used can be found in Additional file 8, Figs. S1-4.

### Assessment of heterogeneity, incoherence, and small-study effects

There was evidence of moderate heterogeneity in the network (*τ*^2^ = 0.087) for efficacy and procedural time (*τ*^2^ = 0.092), while safety heterogeneity was estimated at zero. The design-by-treatment interaction model and the side-splitting method did not suggest statistical incoherence for any outcome (Additional file 9, Tables S1-S2, Figs. S1-S3). The comparison-adjusted funnel plots appeared quite symmetrical, suggesting the absence of important small-study effects for all the outcomes (Additional file 10, Figs. S1-S3).

### Subgroup, meta-regression, and sensitivity analyses

Subgroup analyses did not reveal any noteworthy differences in treatment effects for the two primary outcomes (Additional file 11, analyses 1–6). In addition, when examining the possible impact of effect modifiers with meta-regressions, all coefficients were non-significant and close to zero (Additional file 12, Table S1), with the exception for hypertension, for which a mild significant effect was observed. This is likely due to the extra variability observed in the distribution of hypertension across comparisons (Additional file 5). Still, age and presence of CAD resulted in a 44% and 80% reduction of heterogeneity, respectively.

The sensitivity analyses performed excluding studies with a high risk of bias, studies with patients with PAF, non-irrigated catheters, and the RDN technic did not result in any noteworthy change in the risk of AF recurrence or AF complications (Additional file 13, analyses 1–4).

A sensitivity analysis excluding studies with a PAF-only population (Additional file 13, analysis 3) showed that the addition of lines compared to PVI alone remained more efficient in this population (RR: 0.77, CI: 0.63–0.95). However, PVI combined with RDN lost its statistical superiority compared with PVI alone marginally (RR: 0.59, CI: 0.34–1.03). The subgroup analysis on the type of AF (Additional file 11, analysis 5) [89–99] showed that in studies with PAF-only patients none of the CA strategies outperformed PVI-alone in efficacy.

In order to sum up our results, we performed a sensitivity analysis summing the 19 categories to 6 larger ones (i.e., PVI, PVI and additional lines or substrate modification, PVI and EGM based approach, PVI and combination of substrate modification and EGM based approach, PVI and autonomic modification, and non-PVI strategies). The results of this analysis showed that PVI combined with additional lines or substrate modification, PVI and EGM based approach, and PVI and autonomic modification were more efficacious than PVI alone (RR: 0.81, CI: 0.7–0.93; RR: 0.81, CI: 0.67–0.97; RR: 0.62, CI: 0.46–0.83, respectively). PVI was more efficient than non-PVI strategies (RR: 1.47, CI: 1.18–1.83) (Additional file 13, analysis 6).

Another sensitivity analysis including AADs as comparing arm was performed. This analysis added another 11 RCTs to the 67 of the main analysis, (i.e., in total RCTs) involving 11,248 patients. In accordance with the other sensitivity analyses, this did not result in any important change in the main outcome. Additionally, it revealed that CA strategies including PVI, either as a stand-alone treatment or in combination with other complimentary ablation strategies, were associated with a statistically lower risk of recurrence when compared with ADDs (RRs range from 0.16 (CI: 0.06, 0.47) to 0.47 (CI: 0.32, 0.71) (Additional files 6 and 13).

### Overall credibility of evidence

The CINeMA evaluation suggested that the confidence in the full body of evidence was low for most comparisons both in the efficacy and safety network, with the efficacy network containing a larger proportion of comparisons rated as moderate confidence. Reasons for downgrading to moderate or low certainty were mainly related to the presence of high concerns in imprecision and heterogeneity. This is generally expected in NMAs of non-pharmacological interventions. More details and the specific rules deployed for downgrading are provided in Additional file 14, Tables S1-2.

## Discussion

Catheter ablation has been established as the most effective method for rhythm and symptom control in AF patients [100, 101]. Different strategies are continuously emerging to optimize ablation outcomes. Yet, a lack of agreement about the most effective CA strategy [102] is evident. To address this, we modeled a Network Meta-Analysis after scrutinizing available literature for different CA strategies. In this novel approach, 67 RCTs involving close to 10,000 patients qualified to shed light on different CA strategies with special regard to efficacy and safety.

The foremost findings of this NMA were that: a. PVI with additional sympathetic modulation and PVI with the addition of extra lines seemed to be superior compared to PVI alone, b. There were no differences in safety between different CA-strategies, c. non-PVI strategies were not associated with a better outcome when compared to strategies including PVI and d. All CA strategies that include PVI were superior to AADs with regards to efficacy.

### Differences in efficacy between different CA strategies

PVI has proved to be an effective treatment strategy for AF patients to control symptoms. However**,** the AF population is heterogeneous and for a subset of patients, PVI alone is not sufficient.

As a result, various treatment hypotheses have evolved to different ablation strategies. The rationale for these strategies has support from previous reports that are in clinical use. When summarized, PVI has become the cornerstone for AF ablation, but additional strategies are often used, especially in patients with non-PAF.

The value of additional ablation has been questioned, especially since the publication of the STAR AF 2 study [4], showing the lack of benefit associated with additional ablation. A possible explanation for the results could be that more extensive ablation may cause new areas of arrhythmogenesis. That is, unnecessary ablation and incomplete lines may increase the risk for AF recurrence or atrial tachycardia after the procedure [4]. However, the success of PVI as a stand-alone treatment remains limited, especially in patients with non-PAF [1]. Summarizing our results from evaluating more than 24 RCTs including PVI in combination with additional lines, PVI is a more effective therapy than AADs [101], and no less, there is support for completing this approach with additional lines to enhance the efficacy of the procedure without hampering safety. A sensitivity analysis excluding RCTs with only PAF patients confirmed that the addition of lines to PVI is more efficacious to PVI alone in this category of patients (Additional file 13, analysis 3). These findings are supported by a newly published NMA focusing only on patients with persistent AF [103]. However, in a subgroup analysis (Additional file 11, analysis 5) including only studies with a PAF population, no strategy outperformed PVI alone in efficacy. However, the number of studies in this subgroup analysis was limited, and the result can depend on the lack of power.

Isolation of the pulmonary veins has always been the focus of the CA strategies [94, 104]. Thus, it is not surprising that non-PVI strategies were not as efficient as PVI in this NMA, consistent with previous studies [4, 68, 105]. However, this NMA also showed that adding complementary therapies to PVI, such as GP ablation [52], RDN [77], or performing additional ablation [28, 85] can increase procedural efficacy.

Our results seem plausible with regards to what is known. The Cox/maze procedure is a very effective treatment option for patients with AF undergoing thoracic surgery [106]. It would therefore be reasonable that ablation with bi-atrial modification in combination with PVI is effective. However, this result should be treated with caution as there was only one small study performed before 2011 [27] that included this treatment option, thus the risk of bias is high.

Moreover, RDN and GP ablation as methods of adrenergic modulation in patients with AF have been used for more than 10 years [51, 72]. Research has shown that RDN improves AF outcome, possibly through better blood pressure control and a direct antiarrhythmic effect mediated by sympatholysis [58, 77]. Ganglia plexi ablation as a complementary therapy to PVI can improve CA ablation’s outcome by a more complete autonomic denervation and possibly by ablation of complex electrical activity areas located at the ablated parts of the left atrium [51, 52].

The results of our main analysis are also in line with a sensitivity analysis (Additional file 13, analysis 6) summing up the 19 different strategies to 6. This analysis showed that PVI with additional ablation with either additional lines, substrate modification, or by following EGM-based strategy, or by employing an autonomic modification is more efficient than PVI alone without any costs in safety.

### Safety outcome

Complications are uncommon following the CA of AF. Nevertheless, they remain a major concern. The overall incidence of reported complications was < 5%, with a death rate of < 1%, in line with previous findings [107, 108]. This confirms that CA of AF is a relatively safe procedure, but complication rates are not trivial and should remind us of the importance of a careful selection of patients for CA of AF [109].

Additionally, the complication rates remained low regardless of the CA strategy followed. This finding is of great importance, as the choice of strategy can focus more on patient’s needs than on the fear of complications of more complex procedures. It is also important to remember that most RCTs are performed by high volume academic centers, which can lead to underestimation of the complication risk, especially in more complex procedures [107, 109].

### Procedural time

The procedural time is an aspect that must be taken into consideration concerning the CA of AF, especially when comparing procedures with similar efficacy. This NMA confirms that PVI in combination with supplementary therapies, in particular PVI in combination with additional lines and/or EGM approach, can be more time-consuming.

### CA strategies compared with AADs

In the sensitivity analyses including also AADs as comparator arm, we identified 11 additional RCTs (78 RCTs in total) (Additional file 13, analysis 5). The results showed that regardless of the CA strategy used, except for non-PVI strategies, CA is more effective compared with ADDs. Furthermore, when PVI is combined with adjuvant ablation therapies the results are even better compared with AADs [90]. Our results are in agreement with more recent studies [100] and meta-analyses [101] comparing CA in general [110] or a specific CA strategy with AADs [94]. Nonetheless, these results should be treated with caution due to concerns about transitivity, i.e., comparison of an invasive strategy with a drug treatment (Additional file 6).

### Strengths and limitations

A major strength of this NMA is that it is the first of its kind and extent concerning this topic. With regards to the generalizability of this NMA, patients included in the original studies are assumed to have been sampled from the same theoretical population. However, efficacy is highly dependent on the monitoring strategy employed in the original studies. Thus, perceived differences, particularly between strategies tested in a small number of studies, may be driven by differences in monitoring devices, rather than the ablation strategy. Differences in the type of AF (PAF, non-PAF, or mixed), the length of the blanking period between studies, and the use of AADs after CA of AF can also add to the variability of the results. Furthermore, our long temporal period of inclusion and the different ways used for measuring outcomes (efficacy, safety, and procedural time) between studies may have an impact on the results of our analyses. Nevertheless, the additional analyses that aimed to capture these differences across studies provided similar results. In conclusion, we believe that our results can be generalized since we included RCTs with both PAF and non-PAF patients, using different ablation strategies and various energy sources, supporting applicability to real-world scenarios and clinical practice.

The strict definition of > 30 s of arrhythmia on monitoring is under much debate and can indeed be questioned as a meaningful endpoint for catheter ablation. However, this has been the endpoint used in the original RCTs. Sixteen percent of RCTs were judged to have a high risk of bias. This observation was mainly due to blinding issues, as the operator could not be blinded in the original studies, owing to the nature of the study. Further, the inclusion of RCTs with a high risk of biased data increases the risk of biased inferences. Still, the sensitivity analysis excluding these studies did not change the results. Finally, the nature of the intervention could impose heterogeneity as its efficacy may depend on unmeasurable characteristics.

## Conclusions

In the present NMA, PVI in combination with additional ablation therapy such as autonomic modulation by GP ablation or RDN and additional lines seem to add efficacy when compared to PVI alone. These CA strategies could be considered to yield higher efficacy, without hampering safety. Additionally, CA seems to be superior to AADs apart from non-PVI strategies. This is the first study to provide decision-makers with robust, comprehensive, and comparative evidence about the efficacy and safety of different CA strategies that reflect the available evidence.

## Supplementary Information

**Additional file 1.** PRISMA NMA checklist of items to include when reporting a systematic review involving a network meta-analysis.

**Additional file 2.** Search Strategy (PubMed, Cochrane central database of clinical trials, Web of Science).

**Additional file 3.** Risk of bias assessment, Table S1- [Risk of Bias assessment with domains (67 RCT of the main analysis)].

**Additional file 4.** Characteristics and list of RCTs included in the network meta-analysis, Table S1- [ Characteristics of the 67 RCTs included in the network meta-analysis].

**Additional file 5.** Deviations from the original protocol.

**Additional file 6.** Evaluation of transitivity and additional transitivity boxplots for all comparisons, including also comparisons with AADs: (age distribution, male distribution, hypertension distribution, SHD distribution, CAD distribution, left atrial dimension distribution, left ventricular ejection fraction distribution).

**Additional file 7.** Additional results from pairwise and network meta-analysis. Tables S1-S3, Fig. S1. Table S1- [Relative risk ratios estimated from the network meta-analysis (lower triangle) and pairwise meta-analysis (upper triangle) comparing every pair of the 20 interventions with respect to efficacy.], Table S2- [Relative risk ratios estimated from the network meta-analysis (lower triangle) and pairwise meta-analysis (upper triangle) comparing every pair of the 17 interventions with respect to safety.], Table S3- [Relative risk ratios estimated from the network meta-analysis (lower triangle) and pairwise meta-analysis (upper triangle) comparing every pair of the 18 interventions with respect to procedural time.], Fig. S1- [*P*-scores for the two primary outcomes].

**Additional file 8.** Results from component network meta-analysis. Figures S1-S4. Figure S1- [Network plots from CNMA model for efficacy (a), safety (b) and procedural time (c). Each treatment is represented as a node and an edge exists between two nodes if direct trial evidence is available. The size of each node is proportional to the number of patients involved in each treatment across all trials, while the size of the edges is proportional to the number of studies available in the corresponding comparison]. Figure S2- [Component network forest plots of relative risk ratios for efficacy]. Figure S3- [Component network forest plots of relative risk ratios for safety]. Figure S4- [Component network forest plots of relative risk ratios for procedural time].

**Additional file 9.** Evaluation of inconsistency. Tables S1-S2, Figures S1-S3. Table S1- [Design-by-treatment interaction test, with global *p*-value, Q statistic and degrees of freedom for each outcome]. Table S2- [Results of the inconsistency net-split approach for all outcomes. For each comparison the direct and indirect estimates are provided along with the respective z-values and *p*-values of differences. *P*-values<0.10 indicate significant disagreement between direct and indirect evidence (in red)]. Figure S1- [Forest plots of the net-split approach separating direct and indirect evidence for efficacy]. Figure S2- [Forest plots of the net-split approach separating direct and indirect evidence for safety]. Figure S3- [Forest plots of the net-split approach separating direct and indirect evidence for procedural time].

**Additional file 10.** Investigation of small-study effects. Figures S1-S3. Figure S1- [Comparison-adjusted funnel plot for efficacy]. Figure S2- [Comparison-adjusted funnel plot for safety]. Figure S3- [Comparison-adjusted funnel plot for procedural time].

**Additional file 11.** Subgroup analyses. 1 Depending on AF detection device. 2 Depending on AAD or reablation allowance during the follow-up. 3 Depending on follow-up duration. 4 Depending on publication year. 5. Subgroup analysis on the type of AF included in the original studies (PAF, non-PAF, and mixed). 6 Subgroup analysis depending on the blanking period (cut-off 8 weeks).

**Additional file 12.** Meta-regression. Table S1- [Meta-regression coefficients, alongside Credible Intervals and percentage reduction in heterogeneity for efficacy outcome].

**Additional file 13.** Sensitivity analyses. 1 Excluding high risk of bias RCTs (57 RCTs left). 2 Excluding RCTs with Renal Denervation (RDN) treatment (64 RCTs left). 3 Excluding RCTs with only PAF patients (42 RCTs left). 4 Excluding catheter 8mm, 8mm plus 3.5mm irrigated, 8mm and 4mm irrigated (55 RCTs left). 5 INCLUDING RCTs with antiarrhythmic drugs (AADs) as control arm (78 total RCTs). 6. Sensitivity analysis with reduced categories.

**Additional file 14.** Overall quality of the evidence with CINeMA assessment. Tables S1-S2. Table S1- [Confidence rating for efficacy using CINeMA]. Table S2- [Confidence rating for safety using CINeMA].

## Data Availability

All data analyzed in this study are available in this published article and supplementary material. The references of articles included in this network meta-analysis are presented on the reference list and the background data of the original studies in the Supplementary Material.

## Abbreviations

AAD: Antiarrhythmic drug
AF: Atrial fibrillation
AT: Atrial tachycardia
Bi: Bi-atrial
CA: Catheter ablation
CAD: Coronary artery disease
CINeMA: Confidence in Network-Meta Analysis
CNMA: Component network meta-analysis
Comb: Combination
EGM: Electrocardiogram
GP: Ganglia plexi
LAA: Left atrial appendage
MD: Mean difference
Mod: Modification
NMA: Network Meta-analysis
PAF: Paroxysmal atrial fibrillation
PRISMA: Preferred Reporting Items for Systematic Reviews and Meta-analyses
PVI: Pulmonary vein isolation
RCT: Randomized control study
RDN: Renal denervation
RoB: Risk of bias
RR: Risk ratio
SHD: Structural heart disease
Step: Stepwise
Sub: Substrate
SVC: Superior vena cava
Trig: Trigger
