# Supplementary material for: Comparing efficacy and safety in catheter ablation strategies for atrial fibrillation: a network meta-analysis
Source: BMC Med. 2022 May 31;20:193. doi: 10.1186/s12916-022-02385-2 (PMC9153169; doi:10.1186/s12916-022-02385-2)
Supplement: Supplementary file 2 — Additional file 2. Search Strategy (PubMed, Cochrane central database of clinical trials, Web of Science). [file 12916_2022_2385_MOESM2_ESM.docx]

**Additional file 2. SEARCH STRATEGY**

**Pubmed**

("catheter ablation" OR RFA OR "radiofrequency ablation" OR ablation OR CA OR cryoablation OR cryoballoon OR pulmonary vein isolation ) AND ("auricular fibrillation" OR "atrial fibrillation") AND ("Clinical Trial" [PT:NoExp] OR "clinical trial, phase i"[pt] OR "clinical trial, phase ii"[pt] OR "clinical trial, phase iii"[pt] OR "clinical trial, phase iv"[pt] OR "controlled clinical trial"[pt] OR "multicenter study"[pt] OR "randomized controlled trial"[pt] OR "Clinical Trials as Topic"[mesh:noexp] OR "clinical trials, phase i as topic"[MeSH Terms:noexp] OR "clinical trials, phase ii as topic"[MeSH Terms:noexp] OR "clinical trials, phase iii as topic"[MeSH Terms:noexp] OR "clinical trials, phase iv as topic"[MeSH Terms:noexp] OR "controlled clinical trials as topic"[MeSH Terms:noexp] OR "randomized controlled trials as topic"[MeSH Terms:noexp] OR "early termination of clinical trials"[MeSH Terms:noexp] OR "multicenter studies as topic"[MeSH Terms:noexp] OR “Double-Blind Method”[Mesh] OR ((randomised[TIAB] OR randomized[TIAB]) AND (trial[TIAB] OR trials[tiab])) OR ((single[TIAB] OR double[TIAB] OR doubled[TIAB] OR triple[TIAB] OR tripled[TIAB] OR treble[TIAB] OR treble[TIAB]) AND (blind*[TIAB] OR mask*[TIAB])) OR ("4 arm"[tiab] OR "four arm"[tiab]))

**Cochrane central database of clinical trials**

(¨atrial fibrillation¨ OR ¨auricular fibrillation¨ OR ¨AF¨) AND (¨radiofrequency ablation¨ OR ¨catheter ablation¨ OR ¨ablation¨ OR ¨CA¨ OR ¨cryoablation¨ OR ¨cryoballoon¨ OR ¨pulmonary vein isolation¨)

**Web of science**

(TS=(atrial fibrillation) AND TS=(ablation OR CA OR cryoablation OR cryoballoon OR pulmonary vein isolation) AND TS=(random*)) AND LANGUAGE: (English) AND DOCUMENT TYPES: (Article)
