# Supplementary material for: Comparing efficacy and safety in catheter ablation strategies for atrial fibrillation: a network meta-analysis
Source: BMC Med. 2022 May 31;20:193. doi: 10.1186/s12916-022-02385-2 (PMC9153169; doi:10.1186/s12916-022-02385-2)
Supplement: Supplementary file 3 — Additional file 3. Risk of bias assessment, Table S1- [Risk of Bias assessment with domains (67 RCT of the main analysis)]. [file 12916_2022_2385_MOESM3_ESM.docx]

**Appendix 3. RISK OF BIAS ASSESSMENT**

The Cochrane Collaboration RoB tool for randomized trials (RoB V.2) was used to rate the quality of the included RCTs [10]. RoB V.2 is structured into domains of bias, focusing on different aspects of trial: design (randomization), conduct (deviations from the intended intervention, missing outcome data), and reporting (measurement of the outcome, selection of the reported results). Within each domain, a number of questions (‘signaling questions’) aim to obtain information about features of the trial that are relevant to RoB. An algorithm arising from the reported signaling questions is then applied and the decision about the RoB is generated. A decision can be of ‘low’ or ‘high’ RoB or can express ‘some concerns.^1^ If a study is judged to be at ‘high risk’ of bias in at least one domain or to have ‘some concerns’ for multiple domains that lower the confidence of the result, the overall risk of bias of the particular study will be judged to be of ‘high risk’ of bias. If a study is judged to raise ‘some concerns’ in at least one domain for the result, then the overall risk of bias of the study will also raise some concerns. Finally, if all the domains assessed are of low risk of bias, then the overall judgment of the study will also be of ‘low risk’ of bias [10].

The way missing data were treated has been previously described [9]. In short, in case of missing data, the original authors have been contacted. Missing outcome data have been assessed with the use of RoB.

It is worthwhile noticing that the original studies in this meta-analysis concern the employment of different interventions. Blinding of the operators is not possible. However, blinding of participants is still possible and the domain of blinding has been assessed on rating the RoB of each study.

Table S1 Risk of Bias assessment with domains (67 RCT of the main analysis).

**
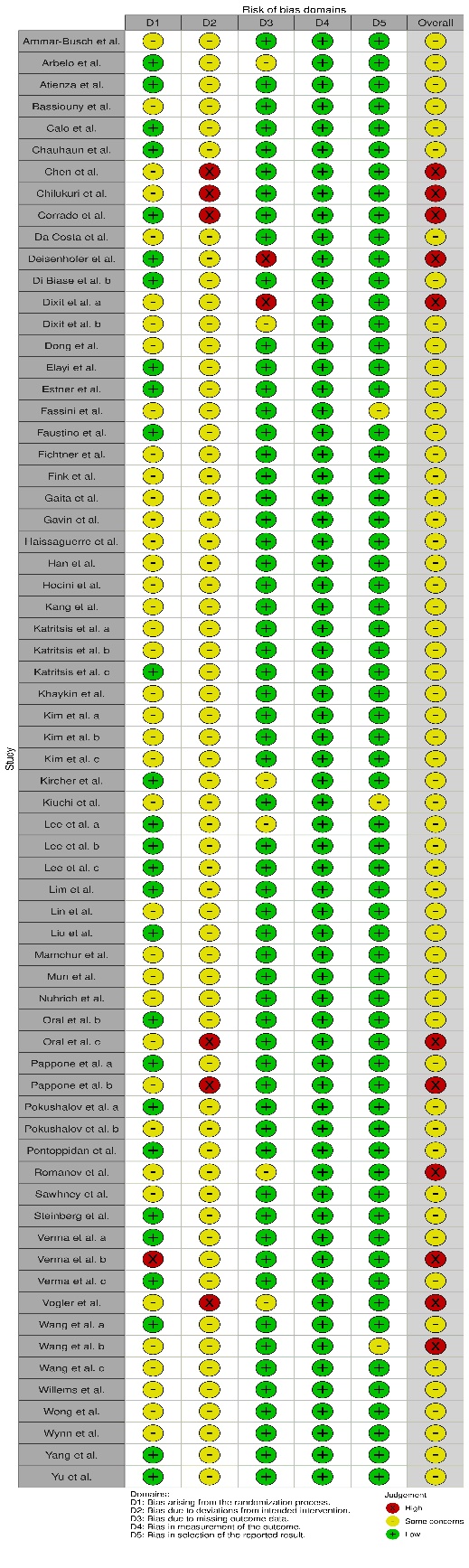
**
