# Supplementary material for: Comparing efficacy and safety in catheter ablation strategies for atrial fibrillation: a network meta-analysis
Source: BMC Med. 2022 May 31;20:193. doi: 10.1186/s12916-022-02385-2 (PMC9153169; doi:10.1186/s12916-022-02385-2)
Supplement: Supplementary file 4 — Additional file 4. Characteristics and list of RCTs included in the network meta-analysis, Table S1- [ Characteristics of the 67 RCTs included in the network meta-analysis]. [file 12916_2022_2385_MOESM4_ESM.docx]

**Additional file 4. RCTs INCLUDED IN THE NETWORK META-ANALYSIS**

**Table S1.** Characteristics of the 67 RCTs included in the network meta-analysis.

| **Study** | **Year of publication** | **blanking period (weeks)** | **follow up period (months)** | **Total number (n)** | **Strategies** | **Number of patients available for analysis of efficacy** | **Age (mean ± SD)** | **Sex (% male)** | **PAF (%)** | **Hypertension (%)** | **CAD (%)** | **CHF (%)** | **SHD (%)** | **LVEF % (mean±SD)** | **LAD mm(mean±SD)** |
| --- | --- | --- | --- | --- | --- | --- | --- | --- | --- | --- | --- | --- | --- | --- | --- |
| Ammar-Busch et al [23] | 2017 | 8 | 12 | 90 | PVI+combination (lines and egm) | 45 | 64 ±9 | 34 (76) | 0 | 38 (84) | 7 (16) | NR | 25 (56) | NR | 49±7 |
|  |  |  |  |  | PVI+EGM | 45 | 65 ±8 | 37 (82) | 0 | 39 (87) | 13 (29) | NR | 26 (58) | NR | 48±6 |
| Arbelo et al. [24] | 2014 | 12 | 12 | 120 | PVI+lines | 59 | 55±11 | 42 (71) | 59 (100) | 19 (32) | 4 (7) | NR | 14 (24) | 62±7 | 41±6 |
|  |  |  |  |  | PVI | 59 | 55±12 | 42 (69) | 59 (100) | 24 (39) | 2 (3) | NR | 7 (12) | 62±5 | 41±6 |
| Atienza et al. [25] | 2014 | 8 | 12 | 232 | PVI+EGM | 54 | 54±12 | 40 (73) | 54 (100) | 24 (44) | NR | NR | 12 (22) | 60±9 | 40±6 |
|  |  |  |  |  | PVI | 58 | 53±10 | 49 (84) | 58 (100) | 17 (29) | NR | NR | 7 (12) | 60±9 | 40±5 |
| Bassiouny et al. [26] | 2016 | 12 | 12 | 90 | PVI+EGM | 44 | 65±9 | 33 (75) | 0 | 27 (61) | 16 (36) | 14 (32) | NR | 60±8 | 42±10 |
|  |  |  |  |  | PVI+posterior wall±lines | 46 | 62±9 | 34 (74) | 0 | 32 (70) | 12 (26) | 18 (39) | NR | 60±8 | 45±9 |
| Calo et al. [27] | 2006 | 6 | 12 | 80 | PVI+biatrial modification | 39 | 58±9 | 26 (65) | 0 | 16 (40) | 8 (21) | NR | 33 (85) | 50±8 | 50±5 |
|  |  |  |  |  | PVI+lines | 41 | 59±9 | 25 (63) | 0 | 18 (45) | 9 (22) | NR | 34 (83) | 51±7 | 51±5 |
| Chauhan et al. [28] | 2020 | 12 | 12 |  | PVI+EGM | 39 | 60±10 | 28 (72) | 21 (54) | 15 (38) | 1 (3) | NR | NR | 59±8 | 42±7 |
|  |  |  |  |  | PVI | 39 | 62±9 | 60 (75) | 21 (51) | 23 (56) | 1 (2) | NR | NR | 58±8 | 42±7 |
| Chen et al. [29] | 2011 | 12 | 12 | 118 | PVI+EGM | 58 | 56 ±11 | 40 (68) | 58 (100) | 12 (20.6) | 2 (3) | NR | NR | 65±3 | 34±4 |
|  |  |  |  |  | PVI | 35 | 52±13 | 39 (67) | 35 (100) | 5 (20.8) | 1 (5) | NR | NR | 66±4 | 35±4 |
|  |  |  |  |  | EGM | 24 | 58±9 | 23 (66) | 24 (100) | 10 (29) | 3 (9) | NR | NR | 66±5 | 36±4 |
| Chilukuri et al. [30] | 2011 | 12 | 10 | 30 | Box isolation | 16 | 62±10 | 9 (56) | 12 (75) | 10 (63) | NR | NR | 5 (31) | 60±9 | 40±5 |
|  |  |  |  |  | PVI | 13 | 58±8 | 9 (69) | 11 (85) | 5 (38) | NR | NR | 3 (23) | 60±7 | 45±8 |
| Corrado et al. [31] | 2010 | 8 | 12 | 320 | PVI+SVC±lines | 134 | 55±10 | 99 (74) | 61 (46) | NR | NR | NR | NR | 54±6 | 45±8 |
|  |  |  |  |  | PVI | 160 | 57±9 | 118 (74) | 73 (46) | NR | NR | NR | NR | 53±7 | 46±6 |
| Da Costa et al. [32] | 2015 | 8 | 15 | 100 | PVI+SVC±lines | 51 | 55±10 | 40 (78) | 51 (100) | 16 (31) | NR | NR | 8 (16) | 63±7 | 42±7 |
|  |  |  |  |  | PVI | 49 | 58±9 | 39 (80) | 49 (100) | 16 (33) | NR | NR | 16 (32) | 64±7 | 39±6 |
| Deisenhofer et al. [33] | 2009 | 0 | 6 | 98 | PVI+EGM | 48 | 55 ± 10 | 41 (82) | 48 (100) | NR | NR | NR | 34 (68) | NR | 44±5 |
|  |  |  |  |  | PVI | 46 | 58 ± 10 | 33 (69) | 46 (100) | NR | NR | NR | 28 (58) | NR | 43±6 |
| Di Biase et al. [34] | 2009 | 8 | 12 | 103 | PVI+EGM | 34 | 58±8 | 30 (88) | 34 (100) | 12 (35) | NR | NR | NR | 54±6 | 41±5 |
|  |  |  |  |  | PVI | 35 | 57±8 | 29 (83) | 35 (100) | 12 (34) | NR | NR | NR | 55±8 | 43±6 |
|  |  |  |  |  | EGM | 34 | 60±9 | 26 (76) | 34 (100) | 13 (38) | NR | NR | NR | 56±6 | 41±5 |
| Dixit et al. [35] | 2008 | 6 | 12 | 105 | PVI+partly | 51 | 57±9 | 36 (69) | 36 (69) | NR | NR | NR | NR | NR | NR |
|  |  |  |  |  | PVI | 52 | 57±9 | 40 (75) | 41 (77) | NR | NR | NR | NR | NR | NR |
| Dixit et al. [36] | 2012 | 6 | 12 | 166 | PVI+EGM | 51 | 60±9 | 46 (90) | 0 | 38 (75) | NR | NR |  | 56±14 | 49±8 |
|  |  |  |  |  | PVI+initiation | 55 | 59±8 | 48 (87) | 0 | 44 (80) | NR | NR | NR | 56±9 | 48±7 |
|  |  |  |  |  | PVI+lines | 50 | 57±10 | 42 (84) | 0 | 30 (60) | NR | NR | NR | 57±10 | 47±6 |
| Dong et al. [37] | 2015 | 12 | 12 | 146 | PVI+stepwise approach | 73 | 56+10 | 56 (77) | 0 | 37(51) | 9 (12) | NR | 14(20) | 61±9 | 43±6 |
|  |  |  |  |  | PVI+lines | 73 | 55+11 | 54 (74) | 0 | 37(51) | 7 (10) | NR | 10 (14) | 62±7 | 42±5 |
| Elayi et al. [38] | 2008 | 8 | 15 | 144 | PVI+EGM | 49 | 59±12 | 32(65) | 0 | 25 (51) | 10 (20) | NR | 23 (47) | 55 | 46±6 |
|  |  |  |  |  | PVI | 47 | 60±10 | 30(64) | 0 | 25 (53) | 7 (15) | NR | 18 (38) | 56 | 46±6 |
|  |  |  |  |  | PVI+posterior wall±lines | 48 | 58±10 | 33 (69) | 0 | 24 (50) | 9 (19) | NR | 22 (46) | 52 | 45±7 |
| Estner et al. [39] | 2011 | 10 | 12 |  | PVI+lines | 59 | 59±10 | 41 (69) | 0 | 34 (58) | 8 (14) | NR | NR | NR | 47±6 |
|  |  |  |  |  | PVI+EGM | 57 | 57±11 | 45 (79) | 0 | 32 (56) | 6 (11) | NR | NR | NR | 49± 6 |
| Fassini et al. [40] | 2005 | 0 | 12 | 187 | PVI+lines | 95 | 54±10 | 73 (77) | 63 (66) | NR | NR | NR | 7( 7) | 55.3 | 43.7 |
|  |  |  |  |  | PVI | 92 | 57±8 | 77 (84) | 63 (68) | NR | NR | NR | 4 (4) | 56.8 | 41.5 |
| Faustino et al. [41] | 2015 | 12 | 12 | 150 | PVI+stepwise approach | 75 | 62±9 | 48 (64) | 75 (100) | 48 (64) | 5 (6.7) | NR | NR | 59±7 | 44±3 |
|  |  |  |  |  | PVI | 75 | 63±8 | 44 (59) | 75 (100) | 56 (75) | 9 (12.0) | NR | NR | 59±7 | 44±3 |
| Fichtner et al. [42] | 2013 | NR | 12 | 207 | PVI partly | 105 | 59±12 | 74 (70) | 105 (100) | 69 (66) | 11 (10.4) | NR | NR | NR | 44±6 |
|  |  |  |  |  | PVI | 102 | 61±9 | 72 (71) | 102 (100) | 57 (56) | 15 (15) | NR | NR | NR | 44±6 |
| Fink et al. [43] | 2017 | 12 | 12 | 124 | PVI+EGM | 57 | 61±10 | 42 (74) | 0 | 29 (51) | 6 (11) | NR | NR | NR | 47±4 |
|  |  |  |  |  | PVI | 61 | 62±10 | 42 (69) | 0 | 35 (57) | 5 (8) | NR | NR | NR | 47±5 |
| Gaita et al. [44] | 2008 | 8 | 12 | 204 | PVI + lines | 137 | 56±10 | 107 (78) | 84 (61) | NR | NR | NR | 21 (15) | NR | NR |
|  |  |  |  |  | PVI | 67 | 53±9 | 55 (82) | 41 (61) | NR | NR | NR | 8 (12) | NR | NR |
| Gavin et al. [45] | 2012 | 12 | 18 | 44 | PVI+lines | 20 | 67±14 | 15 (75) | 20 (100) | 8 (40) | NR | NR | NR | 64.8±12 | 41±10 |
|  |  |  |  |  | PVI | 22 | 68±12 | 15 (68) | 22 (100) | 8 (36) | NR | NR | NR | 64±14 | 41±9 |
| Haissaguerre et al. [46] | 2004 | 4 | 7 | 70 | PVI + lines | 35 | 53±9 | 26 (74) | NR | 7 (20) | NR | NR | 12 (34) | 68±13 | 56±7 |
|  |  |  |  |  | PVI | 35 | 53±8 | 26 (74) | NR | 10 (29) | NR | NR | 18 (51.4) | 65±11 | 54±6 |
| [Han et al.](https://www-ncbi-nlm-nih-gov.e.bibl.liu.se/pubmed/?term=Han%20SW%5BAuthor%5D&cauthor=true&cauthor_uid=24315152) [47] | 2014 | 0 | 12 | 120 | PVI+EGM | 59 | 52±12 | 53 (92) | 0 | 32 (54) | 4 (7) | 3 (5) | NR | 63±9 | 43±7 |
|  |  |  |  |  | PVI + lines | 60 | 54±11 | 54 (90) | 0 | 33 (55) | 7 (12) | 7 (12) | NR | 63±8 | 44±6 |
| Hocini et al. [48] | 2005 | 0 | 12 | 90 | PVI + lines | 45 | 54±10 | 37 (82) | 45 (100) | NR | NR | NR | 10 (22) | 67±8 | 54±8 |
|  |  |  |  |  | PVI | 45 | 55±8 | 34 (76) | 45 (100) | NR | NR | NR | 15 (33) | 67±11 | 51±8 |
| Kang et al. [49] | 2014 | 12 | 12 | 200 | PVI+SVC±lines | 100 | 58+12 | 75 (75) | 100 (100) | 53 (53) | NR | NR | 3 (3) | 65+9 | 40+6 |
|  |  |  |  |  | PVI | 100 | 56+12 | 74 (100) | 100 (100) | 41 (41) | NR | NR | 3 (3) | 63+10 | 40+6 |
| Katritsis et al. [50] | 2004 | NR | 12 | 52 | PVI+partly | 27 | 54±9 | 22 (82) | 27 (100) | 15 (54) | 3 (11) | NR | NR | NR | NR |
|  |  |  |  |  | PVI | 25 | 50±10 | 21 (84) | 25 (100) | 12 (48) | 4 (16) | NR | NR | NR | NR |
| Katritsis et al. [51] | 2011 | 12 | 12 | 160 | PVI+GP | 34 | 55±12 | 25 (74) | 34 (100) | 16 (47) | NR | NR | NR | 56±7 | 42±5 |
|  |  |  |  |  | PVI | 33 | 53±11 | 26 (79) | 33 (100) | 20 (61) | NR | NR | NR | 56±5 | 41±3 |
| Katritsis et al. [52] | 2013 | 12 | 24 | 242 | PVI+GP | 82 | 56±9 | 57 (70) | 82 (100) | 58 (71) | NR | NR | NR | 62±8 | 48±6 |
|  |  |  |  |  | PVI | 78 | 56±8 | 53 (68) | 78 (100) | 63 (81) | NR | NR | NR | 63±7 | 48±7 |
|  |  |  |  |  | GP | 82 | 56±8 | 49 (60) | 82 (100) | 63 (77) | NR | NR | NR | 63±7 | 49±6 |
| Khaykin et al. [53] | 2009 | 8 | 12 | 60 | PVI | 30 | 54±7 | 24 (80) | 25 (83) | 8 (27) | NR | 2 (6) | NR | NR | 38±9 |
|  |  |  |  |  | PVI+lines | 30 | 57±9 | 23 (77) | 23 (77) | 10 (33) | NR | 3 (10) | NR | NR | 38±12 |
| Kim et al. [54] | 2015 | 12 | 12 | 120 | PVI+posterior box±lines | 60 | 56± 12 | 46 (77) | 0 | 25 (42) | NR | 9 (15) | NR | 65±9 | 42±6 |
|  |  |  |  |  | PVI+lines | 60 | 58±10 | 41 (68) | 0 | 29 (48) | NR | 14 (23) | NR | 63±8 | 42±5 |
| Kim et al. [55] | 2017 | 12 | 12 | 137 | PVI+combination (lines and egm) | 54 | 59±11 | 44 (82) | 0 | 29 (54) | NR | 11 (20) | NR | 62±8 | 45±5 |
|  |  |  |  |  | PVI+posterior box±lines | 54 | 63±10 | 35 (65) | 0 | 35 (65) | NR | 12 (22) | NR | 59±10 | 54±6 |
| Kim et al. [56] | 2015 | 12 | 12 | 100 | PVI+posterior box±lines | 50 | 58+11 | 35 (70) | 50 (100) | 22 (44) | NR | 1 (2) | NR | 65+7 | 41+7 |
|  |  |  |  |  | PVI | 50 | 55+12 | 40 (80) | 50 (100) | 16 (32) | NR | 2 (4) | NR | 64+8 | 39+6 |
| Kircher et al. [57] | 2018 | 12 | 12 | 124 | PVI+posterior box±lines | 59 | 62±10 | 36 (58) | 26 (44) | 49 (79) | NR | NR | 12 (19) | 59±9 | 43±6 |
|  |  |  |  |  | PVI+substrate modification | 59 | 63±9 | 41 (66) | 21 (36) | 50 (81) | NR | NR | 12 (19) | 61±7 | 42±6 |
| Kiuchi et al. [58] | 2018 | 12 | 12 | 69 | PVI+RDN | 33 | 57±7 | 25 (76) | 33 (100) | 33 (100) | 5 (15) | NR | NR | 62±7 | NR |
|  |  |  |  |  | PVI | 36 | 58±5 | 30 (83) | 36 (100) | 36 (100) | 9 (25) | NR | NR | 61±6 | NR |
| Lee et al. [59] | 2019 | 12 | 12 | 150 | PVI+combination (lines and egm) | 74 | 56±10 | 47 (64) | 0 | 31 (41) | NR | 4 (5) | NR | 54±6 | 43±6 |
|  |  |  |  |  | PVI+EGM | 71 | 56±10 | 45 (64) | 0 | 32 (43) | NR | 1 (1) | NR | 54±5 | 43±6 |
| Lee et al. [60] | 2018 | 12 | 12 | 500 | PVI+triggers | 229 | 56±11 | 186 (74) | 182 (100) | 111 (44) | NR | 10 (4) | NR | 57±3 | 39±5 |
|  |  |  |  |  | PVI+stepwise approach | 240 | 56±11 | 187 (75) | 178 (100) | 97 (39) | NR | 12 (5) | NR | 56±4 | 39±6 |
| Lee et al. [61] | 2019 | 12 | 16 | 207 | PVI+posterior box±lines | 102 | 59±11 | 88 (86) | 0 | 44 (43) | NR | 23 (22) | NR | 59±9 | 45±5 |
|  |  |  |  |  | PVI | 105 | 59±11 | 84 (80) | 0 | 53 (51) | NR | 24 (23) | NR | 59±10 | 45±7 |
| Lim et al. [62] | 2012 | 12 | 12 | 220 | PVI | 55 | 60±10 | 43 (78) | 34 (62) | 24 (44) | NR | NR | 13 (24) | 57±6 | 43±7 |
|  |  |  |  |  | Box isolation | 55 | 56±12 | 48 (87) | 39 (71) | 23 (42) | NR | NR | 16 (29) | 56±6 | 42.5±7 |
|  |  |  |  |  | PVI+lines | 55 | 60±9 | 42 (76) | 32 (58) | 29 (53) | NR | NR | 13 (24) | 55±9 | 44±9 |
|  |  |  |  |  | Box isolation±lines | 55 | 58±8 | 47 (86) | 30 (55) | 24 (44) | NR | NR | 16 (29) | 55±7 | 44±8 |
| Lin et al. [63] | 2012 | 12 | 12 | 126 | PVI+EGM | 63 | 56 ± 9 | 42 (66) | 30 (48) | 18 (29) | NR | 17 (27) | NR | 61 ± 6 | 39 ± 5 |
|  |  |  |  |  | PVI+triggers | 63 | 53 ± 11 | 40 (64) | 32 (50) | 10 (15) | NR | 11 (18) | NR | 61 ± 8 | 38 ± 7 |
| Liu et al. [64] | 2006 | 12 | 9 | 110 | PVI+lines | 55 | 58±8 | 35 (64) | NR | NR | NR | NR | NR | 63±6 | 37±4 |
|  |  |  |  |  | PVI | 55 | 57±10 | 38 (69) | NR | NR | NR | NR | NR | 64±7 | 38±4 |
| Mamchur et al. [65] | 2014 | 12 | 12 | 120 | PVI | 83 | 57±8 | NR | 0 | NR | 9 (11) | NR | NR | 46±4 | 47±5 |
|  |  |  |  |  | GP | 37 | 56±9 | NR | 0 | NR | 4 (11) | NR | NR | 47±4 | 46±4 |
| Mun et al. [66] | 2012 | 12 | 16 | 156 | PVI+posterior box±lines | 45 | 54±11 | 41 (79) | 45 (100) | 22 (42) | NR | 1 (2) | NR | 64±7 | 41±5 |
|  |  |  |  |  | PVI | 46 | 55±13 | 37 (71) | 46 (100) | 21 (40) | NR | 1 (2) | NR | 65±6 | 39±5 |
|  |  |  |  |  | PVI+lines | 43 | 59±11 | 41 (79) | 43 (100) | 24 (46) | NR | 1 (2) | NR | 64±8 | 40±4 |
| Nuhrich et al. [67] | 2014 | 12 | 12 | 68 | PVI+EGM | 35 | 63+2 | 56 (63) | 35 (100) | 20 (57) | 3 (9) | 0 | NR | 68+1 | 40+1 |
|  |  |  |  |  | PVI | 33 | 59+2 | 20 (61) | 35 (100) | 17 (52) | 2 (6) | 0 | NR | 67+1 | 40+1 |
| Oral et al. [68] | 2005 | 8 | 9 | 80 | Lines | 40 | 55±10 | 34 (85) | 0 | NR | NR | NR | 6 (15) | 53±6 | 49±6 |
|  |  |  |  |  | PVI | 40 | 52±8 | 33 (83) | 0 | NR | NR | NR | 7 (18) | 53±11 | 47±4 |
| Oral et al. [69] | 2003 | 4 | 6 | 80 | PVI | 40 | 51±10 | 31 (78) | 40 (100) | NR | NR | NR | NR | 55±4 | 40±5 |
|  |  |  |  |  | PVI+lines | 40 | 54±11 | 31 (78) | 40 (100) | NR | NR | NR | NR | 57±5 | 41±6 |
| Pappone et al. [70] | 2004 | 6 | 12 | 560 |  | 280 | 57±8. | 153 (55) | 170 (61) | NR | 42 (15) | NR | 106 (38) | NR | 40±4 |
|  |  |  |  |  | PVI | 280 | 56±7 | 138 (49) | 184 (65.7) | NR | 43 (15) | NR | 118 (42) | NR | 40±4 |
| Pappone et al. [71] | 2018 | 12 | 12 | 81 | PVI+combination (lines and egm) | 41 | 64±10 | 29 (71) | 0 | 22 (54) | NR | NR | NR | 56±7 | NR |
|  |  |  |  |  | PVI+posterior box±lines | 40 | 60±11 | 31 (77) | 0 | 26 (43) | NR | NR | NR | 54±6 | NR |
| Pokushalov et al. [72] | 2012 | 12 | 12 | 27 | PVI+RDN | 13 | 57 ± 8 | 10 (77) | 5 (39) | 13 (100) | 2 (15) | NR | NR | 65±5 | 49±7 |
|  |  |  |  |  | PVI | 14 | 56 ± 9 | 11 (79) | 4 (29) | 14 (100) | 2 (14) | NR | NR | 66±4 | 50±6 |
| Pokushalov et al. [73] | 2013 | 12 | 12 | 264 | PVI+GP | 132 | 55±6 | 101 (77) | 0 | 43(33) | NR | NR | 49 (37) | 55±5 | 49±7 |
|  |  |  |  |  | PVI+lines | 132 | 54±7 | 105 (88) | 0 | 46(35) | NR | NR | 41 (31) | 54±6 | 48±7 |
| Pontoppidan et al. [74] | 2009 | 12 | 12 | 149 | PVI+lines | 73 | 56±8 | 74 (50) | 38 (52) | 21 (29) | 3 (4) | 16 (22) | NR | 60±10 | 48±7 |
|  |  |  |  |  | PVI | 76 | 56±8 | 68 (46) | 42 (55) | 35 (46) | 2 (3) | 16 (21) | NR | 64±9 | 46±6 |
| Romanov et al. [75] | 2015 | 12 | 24 | 96 | PVI+LAA occlusion | 45 | 60±5 | 28 (62) | 24 (53) | 38 (84) | NR | NR | NR | 62±5 | 49±6 |
|  |  |  |  |  | PVI | 44 | 60±6 | 26 (59) | 25 (57) | 33 (75) | NR | NR | NR | 61±4 | 48±7 |
| Sawhney et al. [76] | 2010 | 12 | 12 | 66 | PVI+lines | 33 | 59±10 | 25 (76) | 33 (100) | NR | NR | NR | NR | 61±4 | 37±4 |
|  |  |  |  |  | PVI | 33 | 55±12 | 23 (70) | 33 (100) | NR | NR | NR | NR | 62±6 | 36±3 |
| Steinberg et al. [77] | 2020 | 12 | 12 | 302 | PVI+RDN | 147 | 59 (54-65) | 91 (59) | 154 (100) | 154 (100) | 14 (9) | NR | NR | 62±5 | 48±3 |
|  |  |  |  |  | PVI | 141 | 60 (58-65) | 91 (62) | 148(100) | 148(100) | 10 (7) | NR | NR | 62±5 | 47±3 |
| Verma et al. [78] | 2010 | 12 | 12 | 100 | PVI+EGM | 34 | 59±10 | 25 (74) | 22 (65) | 14 (41) | 3 (9) | 0 | NR | 59±12 | 41±6 |
|  |  |  |  |  | PVI | 32 | 55±11 | 24 (75) | 21 (66) | 16 (50) | 1 (3) | 2 (6) | NR | 62±7 | 43±5 |
|  |  |  |  |  | EGM | 34 | 57±9 | 25 (74) | 21 (62) | 15 (44) | 3 (9) | 1 (3) | NR | 64±10 | 41±6 |
| Verma et al. [79] | 2007 | 8 | 12 | 200 | PVI+combination (lines and egm) | 40 | 57±12 | 25 (63) | 0 | 19 (48) | NR | NR | 14 (34) | 53±11 | 43±10 |
|  |  |  |  |  |  | 40 | 56±9 | 25 (63) | 0 | 18 (46) | NR | NR | 12 (31) | 53±12 | 42±9 |
| Verma et al. [4] | 2015 | 12 | 18 | 589 | PVI+EGM | 244 | 60±9 | 213 (81) | 0 | 143 (54) | 21 (8) | 10 (4) | NR | 57±10 | 44±6 |
|  |  |  |  |  | PVI | 61 | 58±10 | 52 (78) | 0 | 32 (48) | 2 (3) | 3 (4) | NR | 55±11 | 44±6 |
|  |  |  |  |  | PVI+lines | 244 | 61±9 | 196 (76) | 0 | 158 (61) | 29 (11) | 15 (6) | NR | 57±10 | 46±6 |
| Vogler et al. [80] | 2015 | 12 | 12 | 205 | PVI+stepwise approach | 71 | 61±11 | 60 (80) | 0 | 57 (76) | 12 (16) | NR | NR | 60±7 | 44±5 |
|  |  |  |  |  | PVI | 71 | 63±10 | 56 (72) | 0 | 65 (83) | 21 (27) | NR | NR | 60±7 | 45±7 |
| Wang et al. [81] | 2014 | 12 | 12 | 124 | PVI+stepwise approach | 60 | 62±6 | 35 (58) | 0 | 24 (40) | 3 (7.7) | NR | NR | 64± 5 | 33±16 |
|  |  |  |  |  | PVI+substrate modification | 64 | 63±11 | 41 (64) | 0 | 26 (41) | 4 (6.2) | NR | NR | 65±6 | 41±8 |
| Wang et al. [82] | 2013 | 4 | 12 | 210 | PVI+combination (lines and egm) | 140 | 63 | 123 (88) | 0 | 46 (33) | NR | 17 (12) | NR | 56.5 | 46 |
|  |  |  |  |  | PVI+EGM | 70 | 64 | 30 (43) | 0 | 37 (53) | NR | 13 (19) | NR | 58 | 47 |
| Wang et al. [83] | 2008 | 4 | 12 | 106 | PVI+SVC±lines | 54 | 65+9 | 30 (58) | 54 (100) | 12 (23) | 4 (8) | NR | NR | 62+5 | 37+3 |
|  |  |  |  |  | PVI | 52 | 67+9 | 28 (52) | 54 (100) | 10 (19) | 3 (6) | NR | NR | 62+4 | 37+3 |
| Willems et al. [84] | 2006 | 4 | 16 | 62 | PVI+lines | 32 | 58+12 | NR | 0 | NR | NR | NR | 4 (13) | NR | 47±6 |
|  |  |  |  |  | PVI | 30 | 60+9 | NR | 0 | NR | NR | NR | 4 (13) | NR | 48±4 |
| Wong et al. [85] | 2015 | 12 | 12 | 130 | PVI+combination (lines and egm) | 65 | 61±11 | 50 (77) | 0 | NR | NR | NR | NR | NR | 45±6 |
|  |  |  |  |  | PVI+lines | 65 | 61±9 | 48 (74) | 0 | NR | NR | NR | NR | NR | 45±6 |
| Wynn et al. [86] | 2016 | 12 | 12 | 124 | PVI+lines | 63 | 62±11 | 41 (62) | 26 (41) | 42 (64) | NR | NR | NR | 62±13 | 43±6 |
|  |  |  |  |  | PVI | 59 | 62±10 | 47 (73) | 25 (42) | 33 (52) | NR | NR | NR | 61±11 | 43±6 |
| Yang et al. [87] | 2017 | 12 | 18 | 229 | PVI+substrate modification | 114 | 57±10 | 92 (81) | 0 | 62 (54) | 9 (8) | 3 (3) | NR | 62±8 | 41±5 |
|  |  |  |  |  | PVI+stepwise approach | 114 | 58±8 | 84 (74) | 0 | 63 (55) | 2 (2) | 2 (2) | NR | 62±7 | 41±5 |
| Yu et al. [88] | 2017 | 12 | 18 | 113 | PVI+posterior box±lines | 54 | 59±9 | 41 (76) | NR | 28 (52) | NR | 10 (17) | NR | 63±8 | 43±6 |
|  |  |  |  |  | PVI | 59 | 61±11 | 44 (75) | NR | 32 (54) | NR | 19 (17) | NR | 61±11 | 43±5 |
