# Supplementary material for: Comparing efficacy and safety in catheter ablation strategies for atrial fibrillation: a network meta-analysis
Source: BMC Med. 2022 May 31;20:193. doi: 10.1186/s12916-022-02385-2 (PMC9153169; doi:10.1186/s12916-022-02385-2)
Supplement: Supplementary file 5 — Additional file 5. Deviations from the original protocol. [file 12916_2022_2385_MOESM5_ESM.docx]

**Additional file 5 DEVIATIONS FROM THE ORIGINAL PROTOCOL**

The secondary endpoint of all-cause mortality was not analyzed due to the large number of studies with zero events in both arms.

Studies including AADs as a comparing arm were excluded from the main analysis. This deviation from the original protocol was decided mainly due to transitivity issues. It seemed from the transitivity analysis that the distributions of the main effect modifiers were not the same between studies that included AADs and those with only CA-arms. Studies comparing AADs with CA were included in a sensitivity analysis.

A post-hoc subgroup analysis was performed concerning the year of publication of the included studies. This analysis was performed to reveal possible sources of heterogeneity depending on whether the data were recent.
