# Supplementary material for: Comparing efficacy and safety in catheter ablation strategies for atrial fibrillation: a network meta-analysis
Source: BMC Med. 2022 May 31;20:193. doi: 10.1186/s12916-022-02385-2 (PMC9153169; doi:10.1186/s12916-022-02385-2)

**Additional file 6. EVALUATION OF TRANSITIVITY**

In network meta-analysis, transitivity refers to the assumption that the relative effect between treatment A and treatment B can be validly compared indirectly through one or more intermediate treatments. To evaluate this, we show boxplots for the distributions of seven potential effect modifiers: mean age, percentage of males, presence of hypertension, coronary artery disease (CAD), structural heart disease (SHD), left atrial dimensions, and left ventricle (LV) ejection fraction (EF) across the available direct comparisons. If no important differences in the distributions of effect modifiers are found, the transitivity assumption is considered to hold in the network.

There were a few outliers in the hypertension distribution. This can be explained by the fact that a number of studies tested different catheter ablation strategies in the subgroup of patients with AF and hypertension.


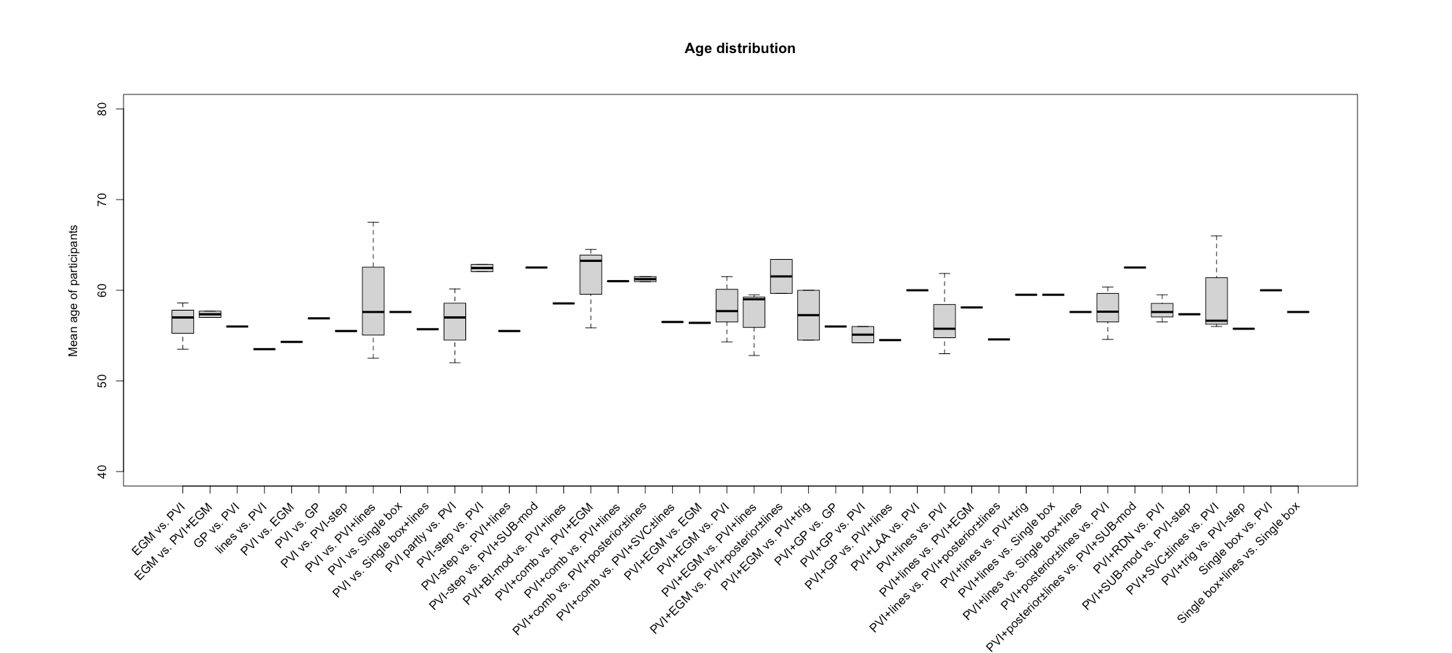

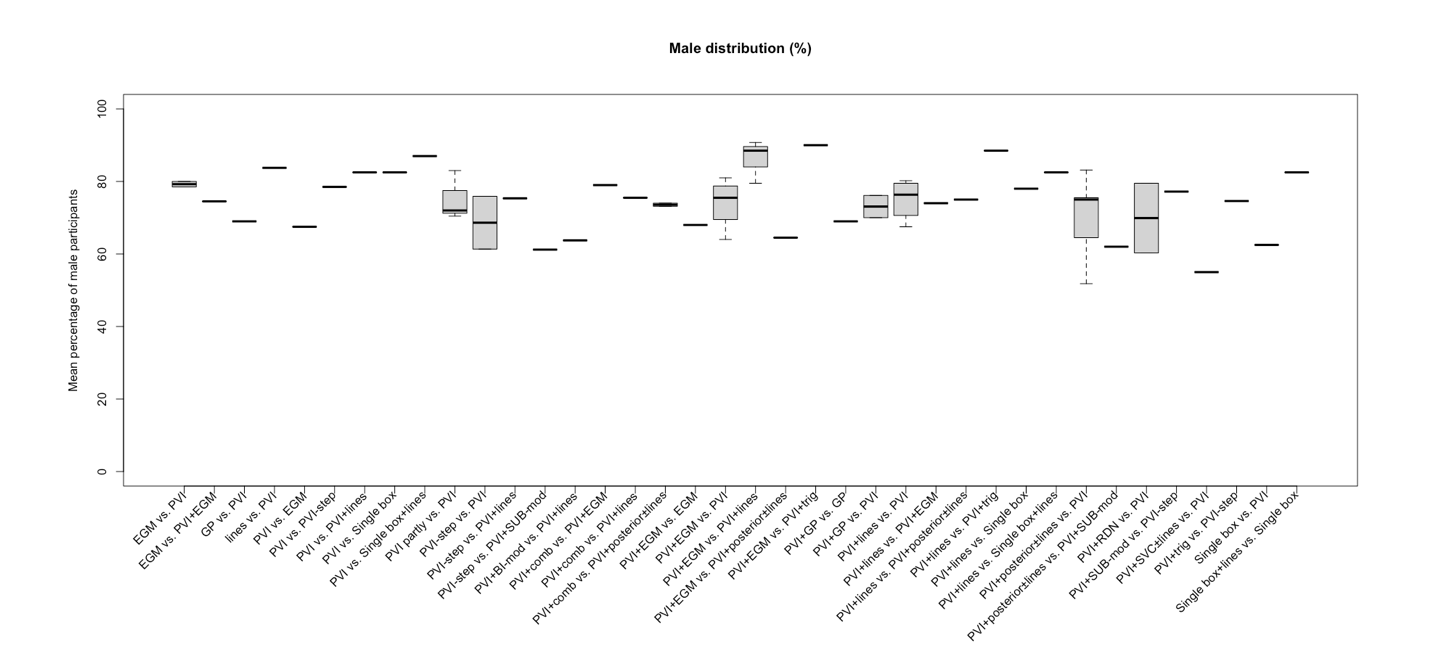

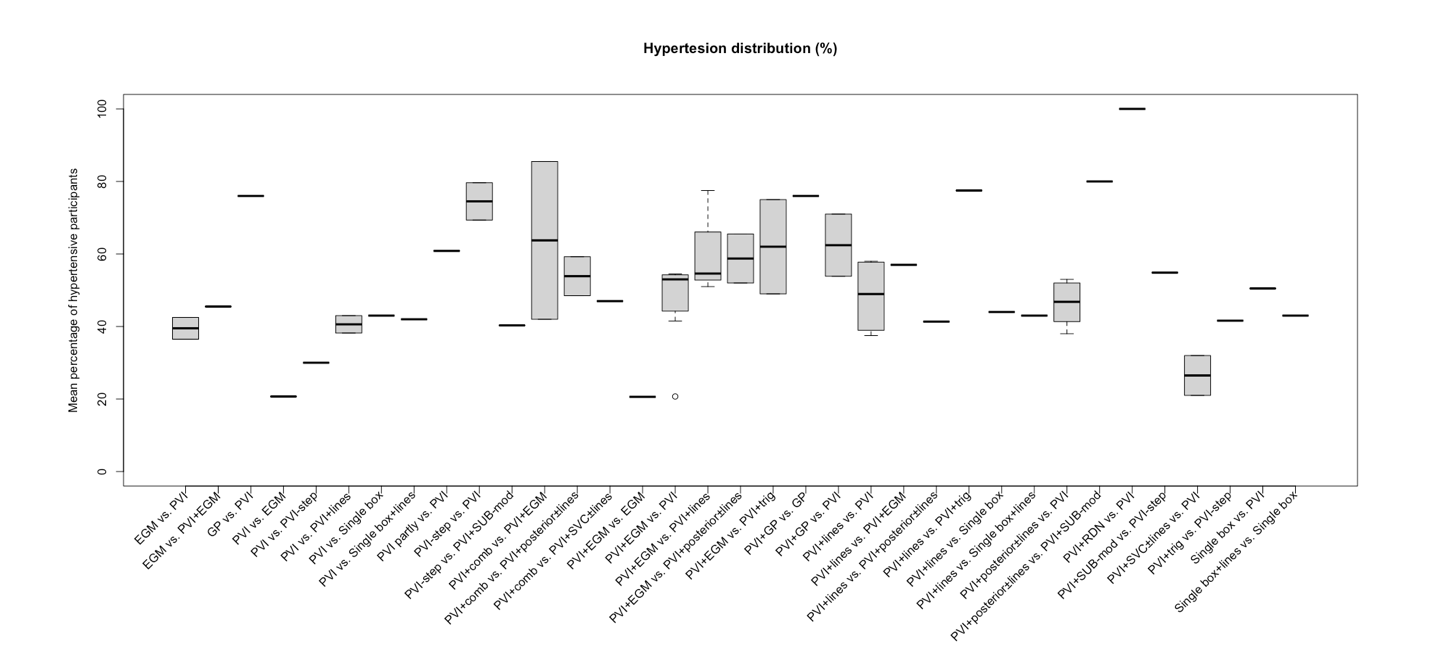


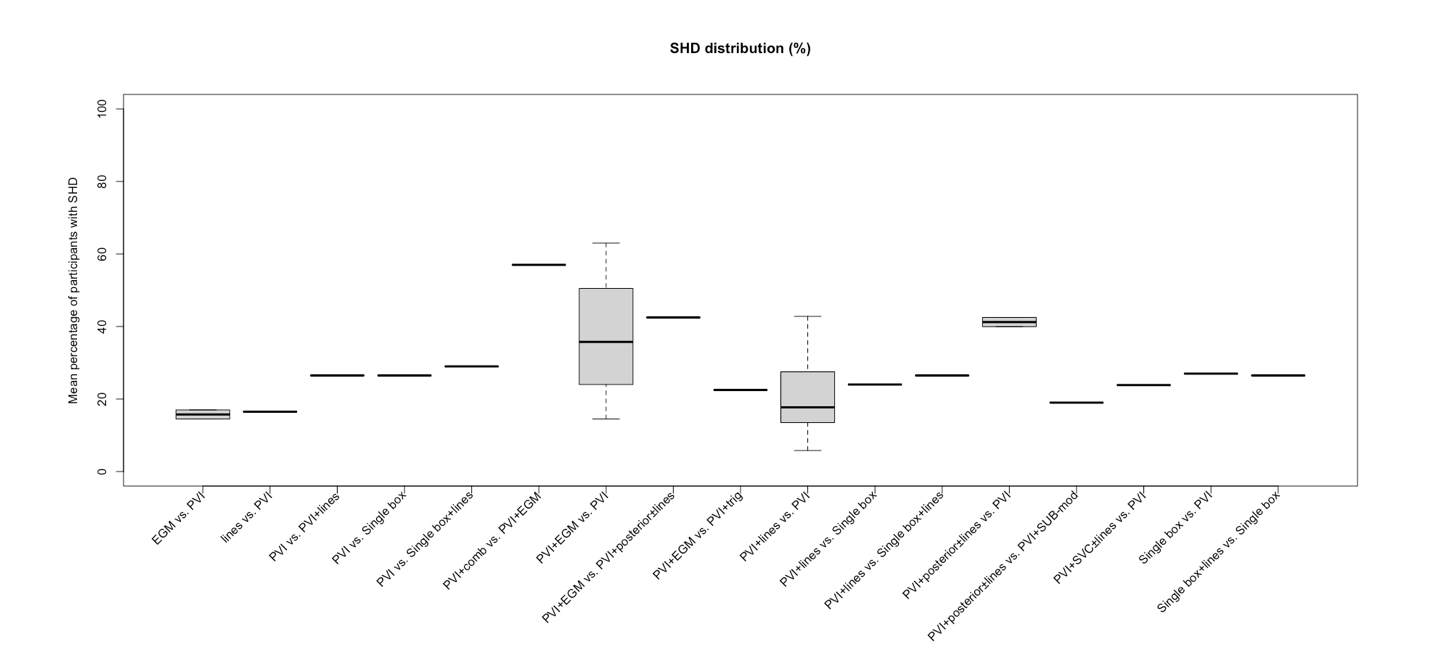

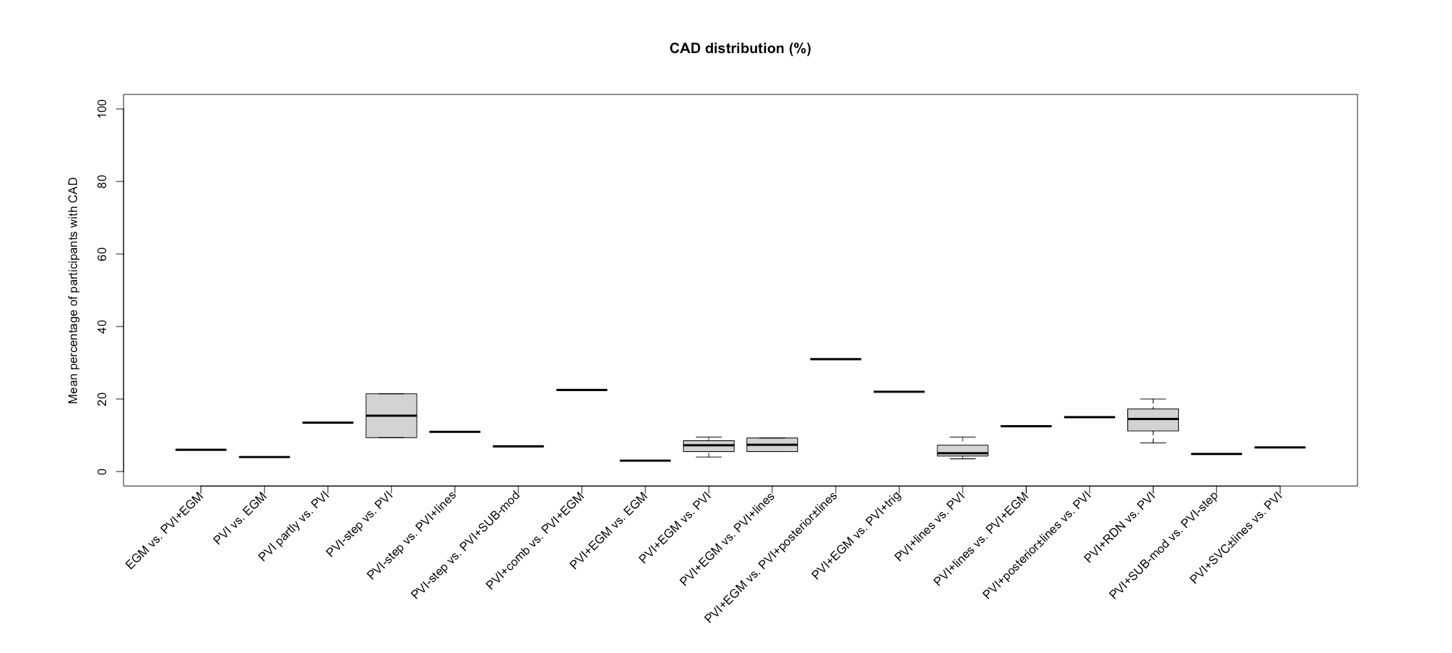


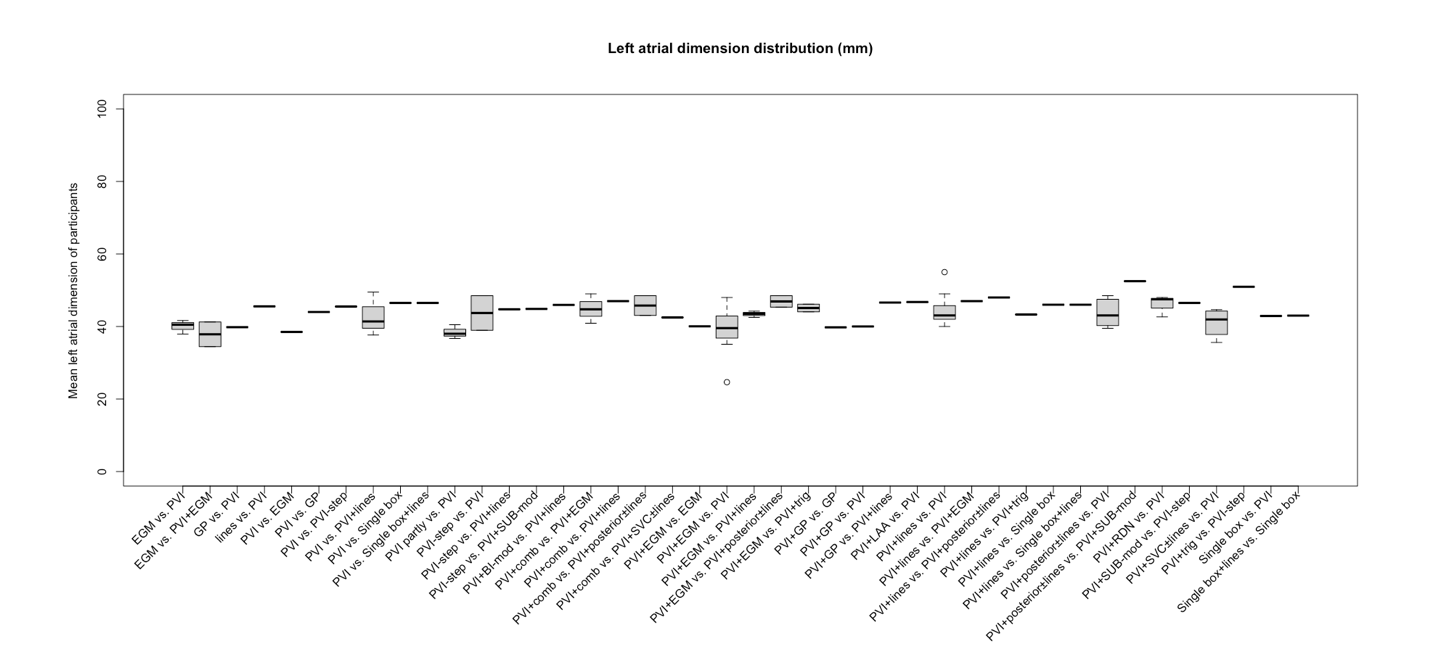

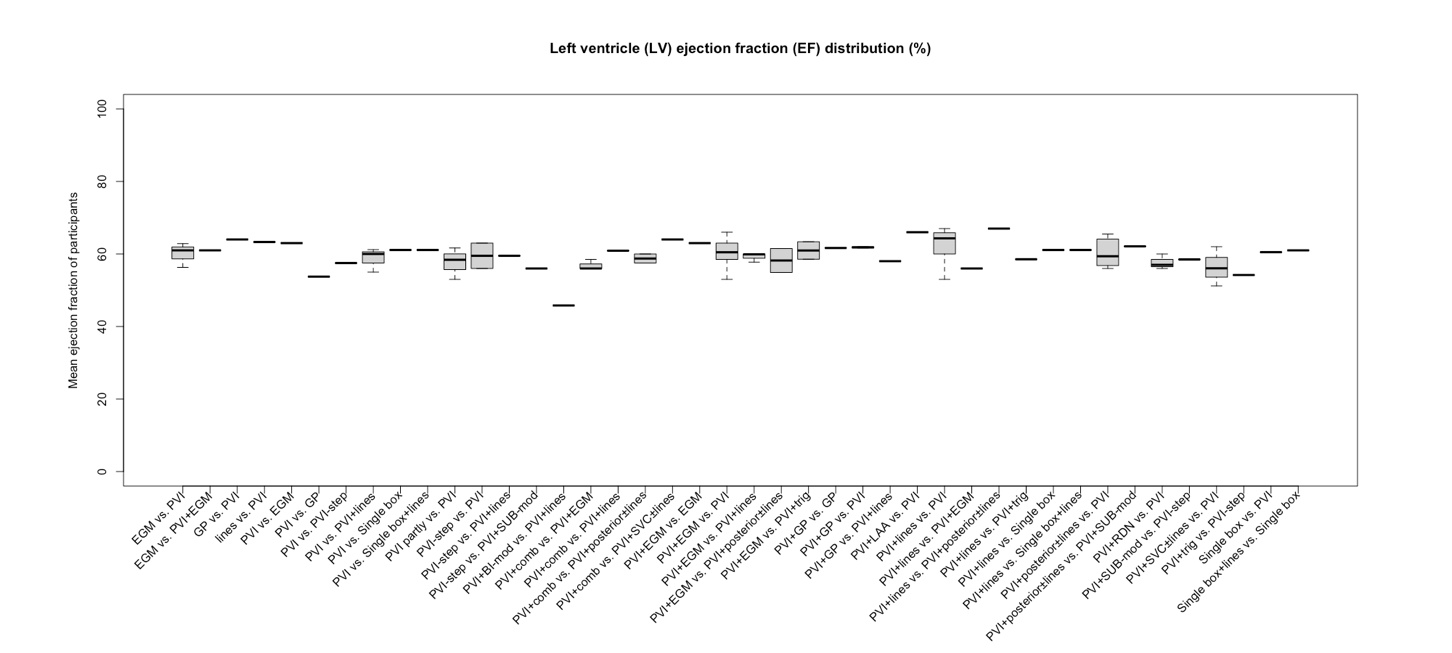


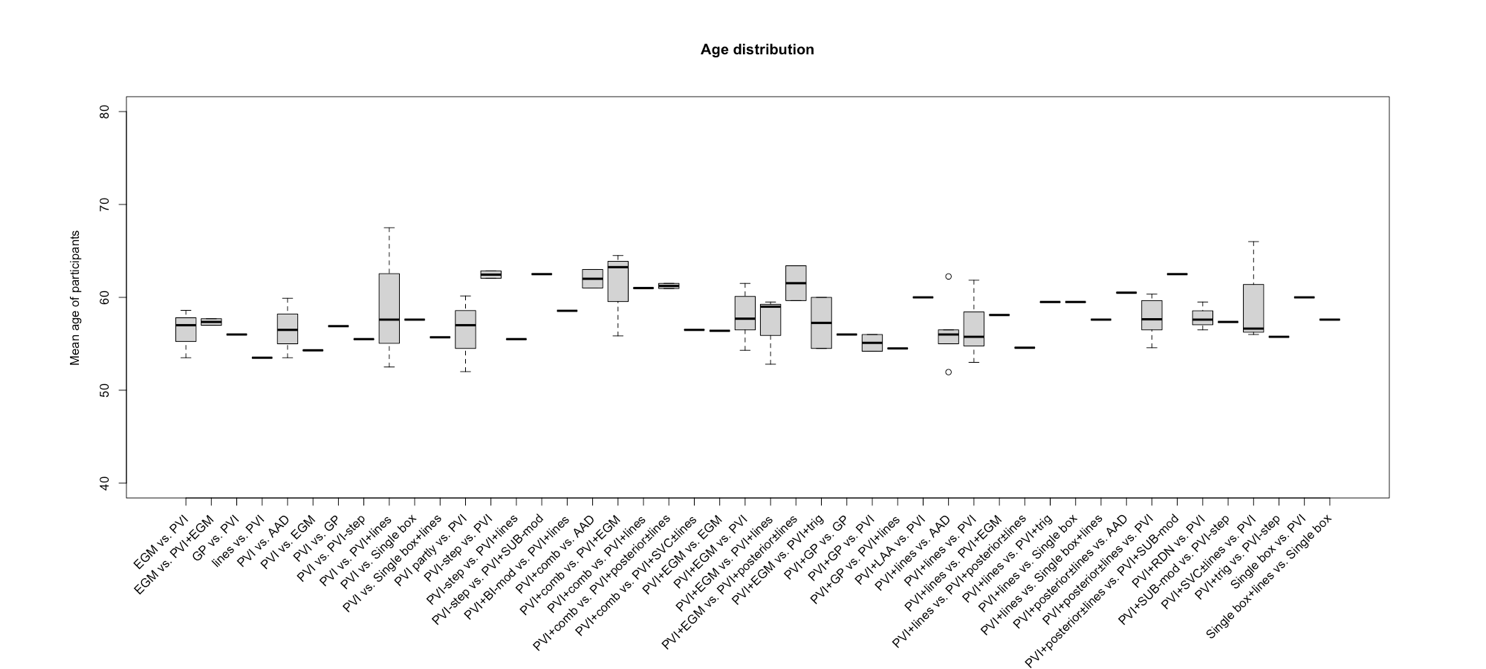

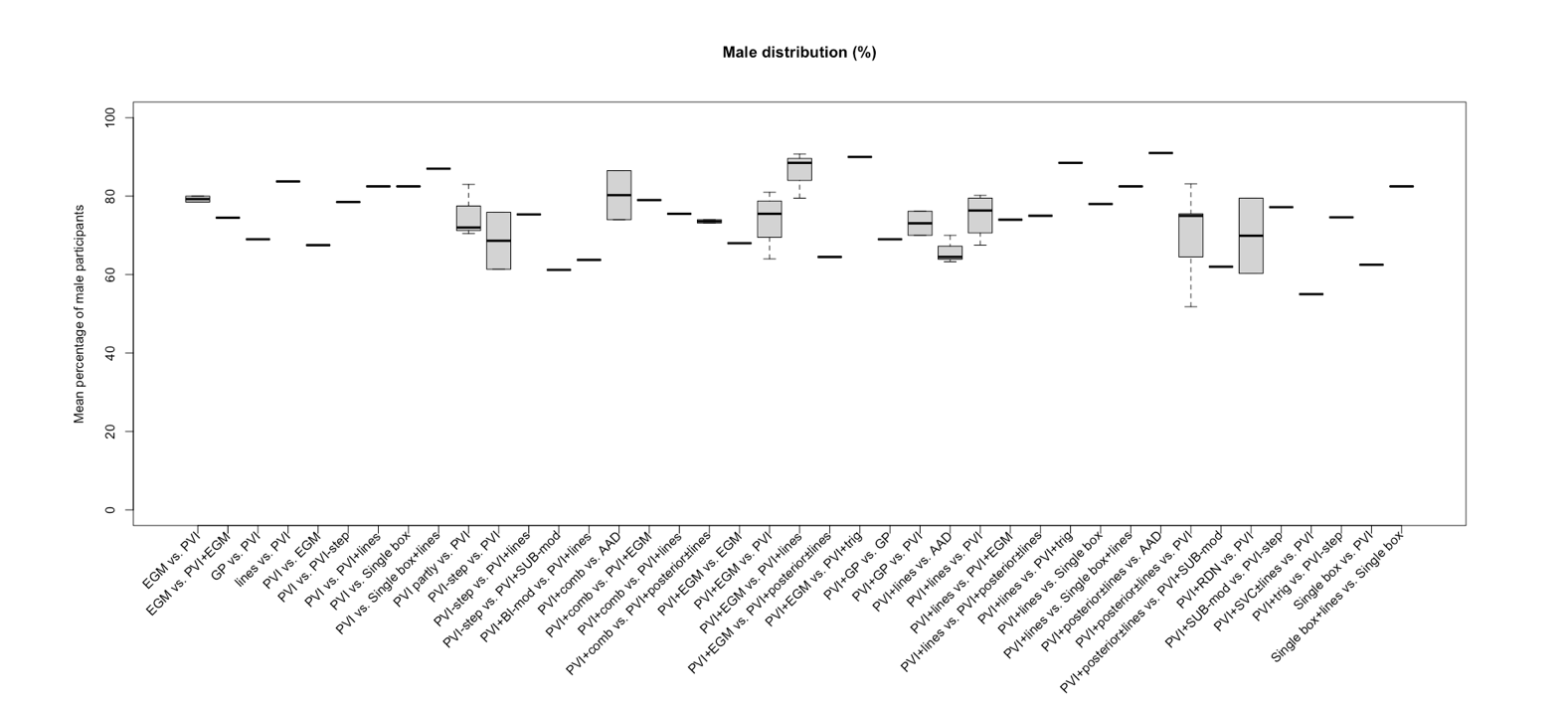
 **Additional transitivity boxplots for all comparisons including also comparisons with AADs:**

As above, we show boxplots for the distributions of mean age, percentage of males, presence of hypertension, coronary artery disease (CAD), structural heart disease (SHD), left atrial dimensions, and left ventricle (LV) ejection fraction (EF) across the available direct comparisons including also comparisons with AADs.


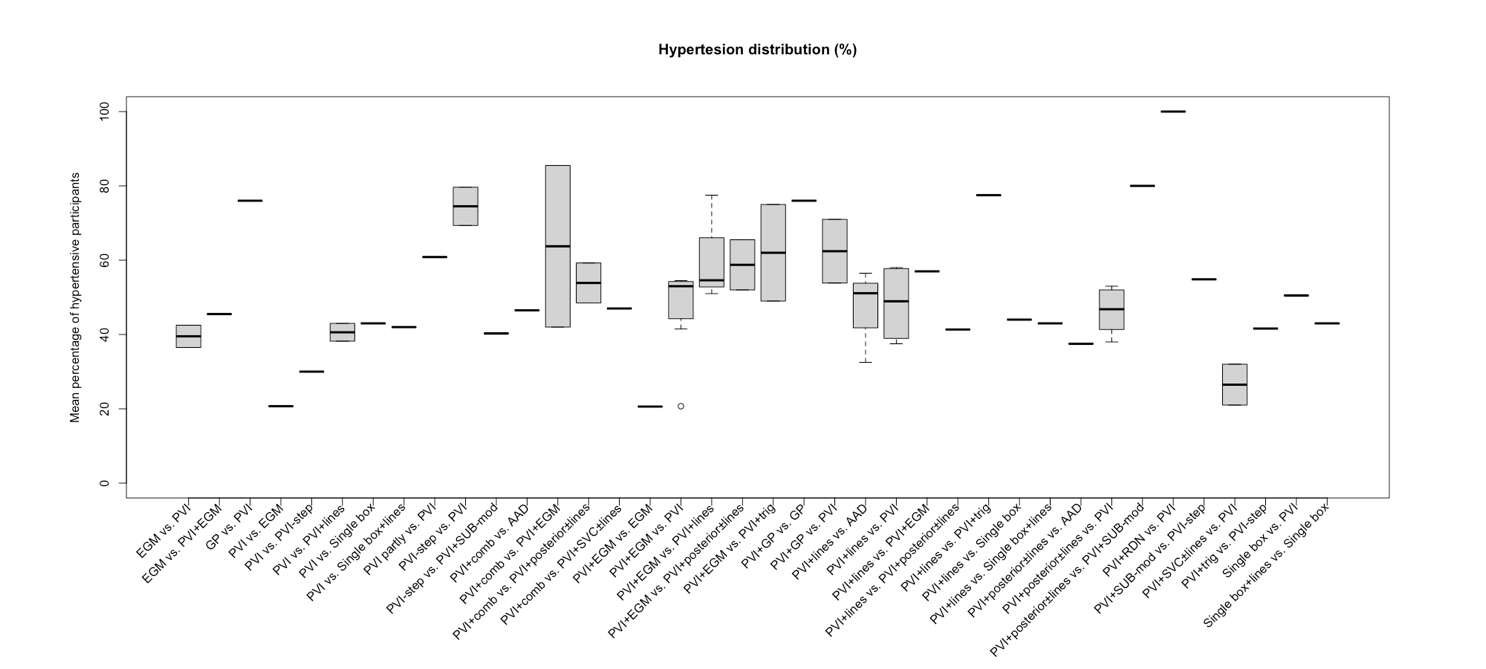

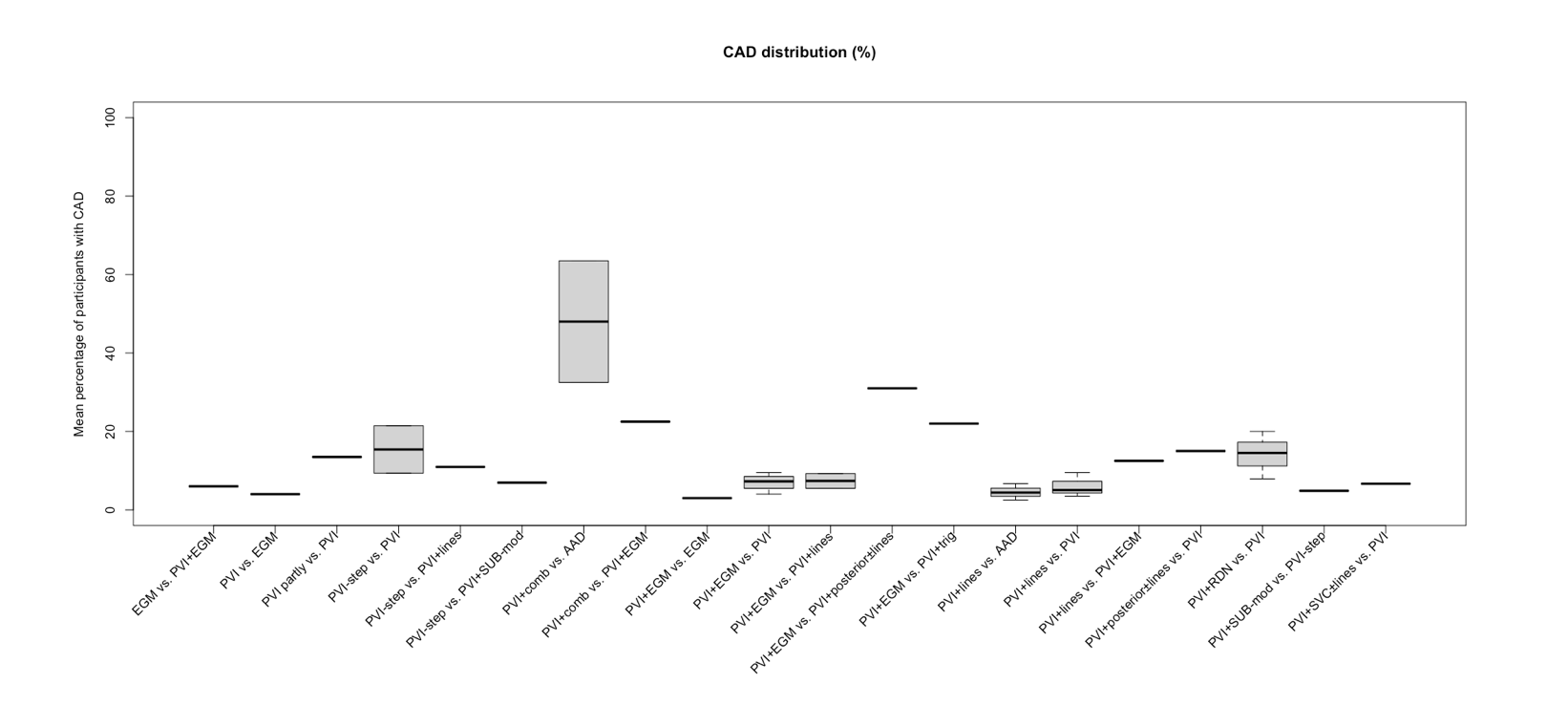

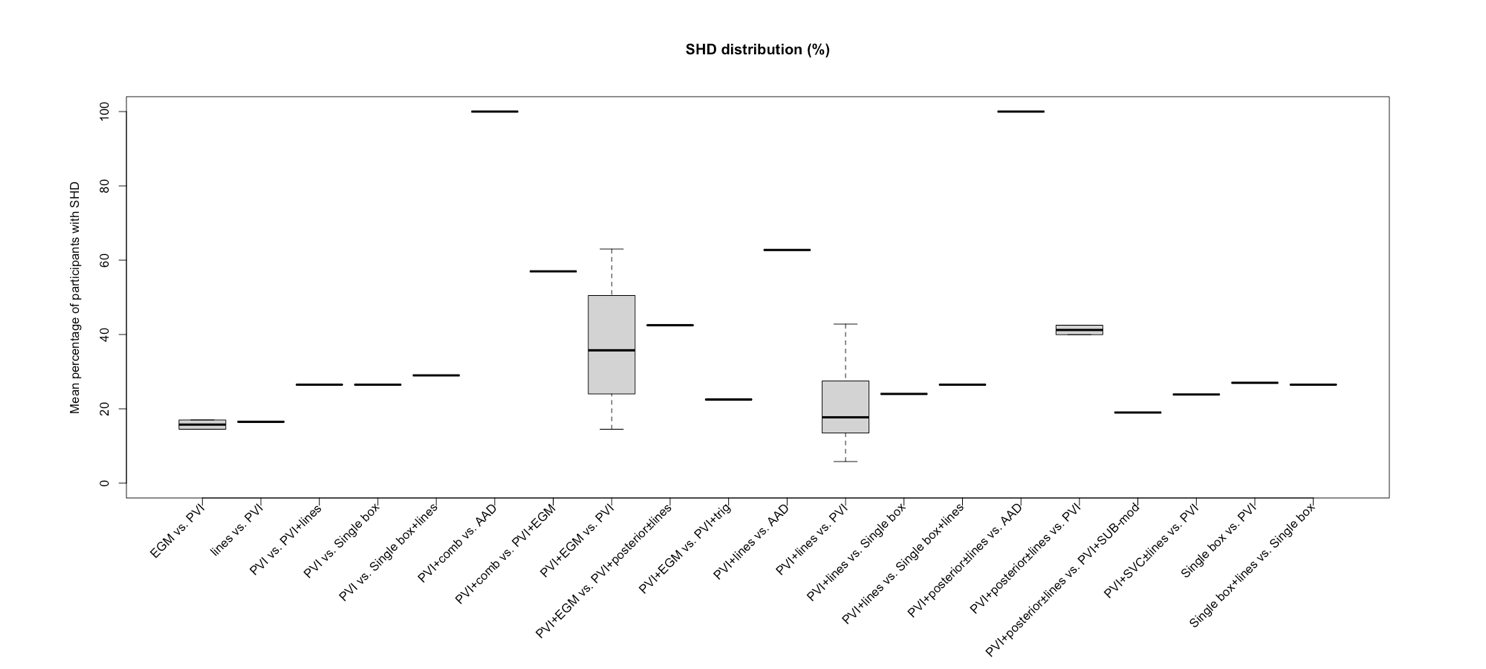


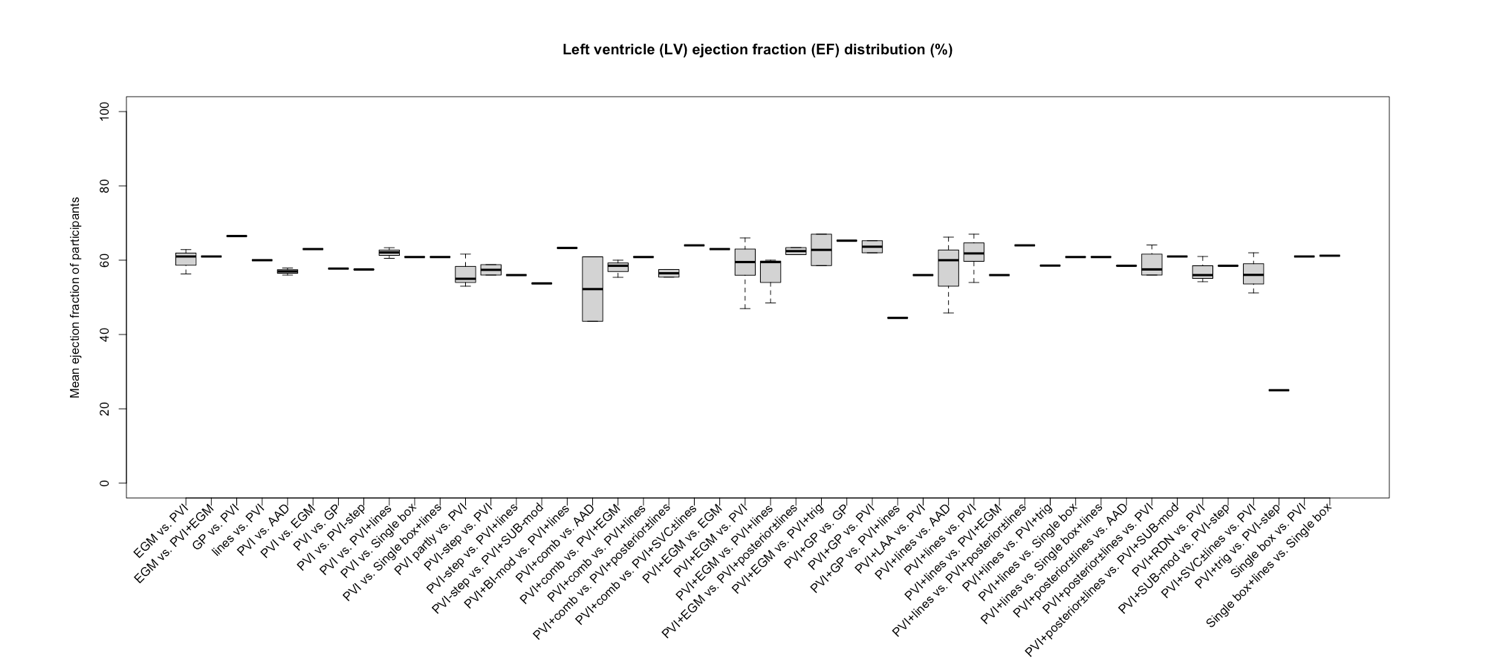


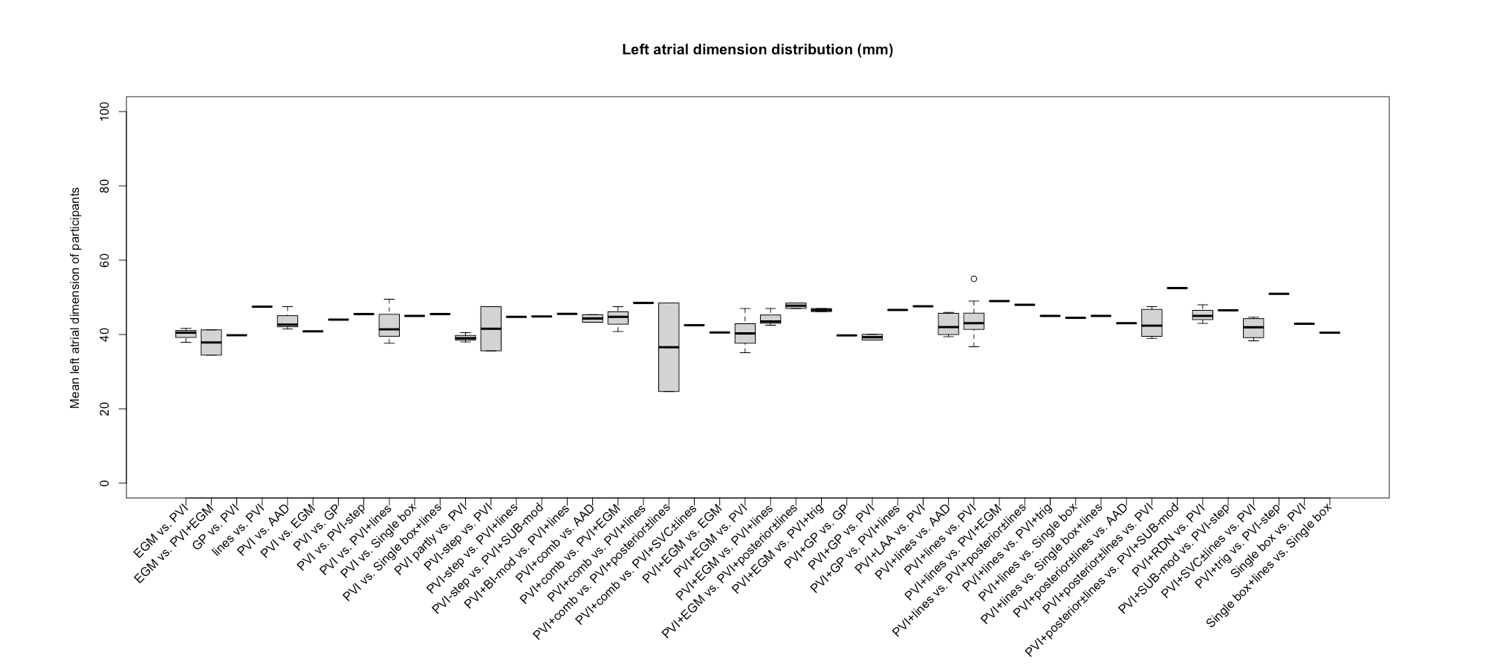

Supplement: Supplementary file 6 — Additional file 6. Evaluation of transitivity and additional transitivity boxplots for all comparisons, including also comparisons with AADs: (age distribution, male distribution, hypertension distribution, SHD distribution, CAD distribution, left atrial dimension distribution, left ventricular ejection fraction distribution). [file 12916_2022_2385_MOESM6_ESM.docx]
