# Supplementary material for: Comparing efficacy and safety in catheter ablation strategies for atrial fibrillation: a network meta-analysis
Source: BMC Med. 2022 May 31;20:193. doi: 10.1186/s12916-022-02385-2 (PMC9153169; doi:10.1186/s12916-022-02385-2)
Supplement: Supplementary file 7 — Additional file 7. Additional results from pairwise and network meta-analysis. Tables S1-S3, Fig. S1. Table S1- [Relative risk ratios estimated from the network meta-analysis (lower triangle) and pairwise meta-analysis (upper triangle) comparing every pair of the 20 interventions with respect to efficacy.], Table S2- [Relative risk ratios estimated from the network meta-analysis (lower triangle) and pairwise meta-analysis (upper triangle) comparing every pair of the 17 interventions with respect to safety.], Table S3- [Relative risk ratios estimated from the network meta-analysis (lower triangle) and pairwise meta-analysis (upper triangle) comparing every pair of the 18 interventions with respect to procedural time.], Fig. S1- [P-scores for the two primary outcomes]. [file 12916_2022_2385_MOESM7_ESM.docx]

**Additional file 7. ADDITIONAL RESULTS FROM PAIRWISE AND NETWORK META-ANALYSIS**

We employed a random-effects model with common heterogeneity across comparisons, and we fitted the models for pairwise and network meta-analysis using the R library ‘netmeta’ (R version 4.0.2, netmeta version 6.6-6).

Additional results in this Appendix: league tables of RRs from NMA for each outcome (eTable2, eTable3, eTable4). and P-scores for ranking treatments for efficacy and safety (eFigure2).

**Table S1.** Relative risk ratios estimated from the network meta-analysis (lower triangle) and pairwise meta-analysis (upper triangle) comparing every pair of the 20 interventions with respect to efficacy.

| PVI | 1.14 (0.93; 1.40) | 1.23 (0.96; 1.57) | 1.84  (1.06; 3.20) | 1.18 (0.86; 1.63) | 1.67  (1.07; 2.61) | 0.85 (0.39; 1.87) | . | . | 1.59 (1.01; 2.50) | . | . | 0.98 (0.65; 1.48) | 0.88 (0.56; 1.39) | 1.10 (0.68; 1.77) | 1.20 (0.61; 2.37) | 0.75 (0.35; 1.61) | 0.71 (0.44; 1.14) | 0.59 (0.39; 0.88) |
| --- | --- | --- | --- | --- | --- | --- | --- | --- | --- | --- | --- | --- | --- | --- | --- | --- | --- | --- |
| 1.25 (1.05;  1.48) | PVI+  lines | 0.82 (0.59; 1.14) | 1.15  (0.62; 2.13) | 1.29 (0.44; 3.76) | . | . | 0.80 (0.41; 1.57) | 0.83 (0.41; 1.67) | . | 2.54 (0.93; 6.94) | . | 0.83 (0.40; 1.69) | . | 0.96 (0.49; 1.90) | 1.08 (0.54; 2.16) | . | . | . |
| 1.21 (0.99;  1.46) | 0.97 (0.78;  1.20) | PVI+  EGM | . | 0.86 (0.53; 1.37) | . | . | 0.90 (0.61; 1.33) | 1.02 (0.61; 1.70) | . | . | . | . | . | . | . | . | . | 0.35 (0.21; 0.58) |
| 1.60 (1.06;  2.42) | 1.29 (0.85;  1.95) | 1.33 (0.85;  2.07) | PVI+  GP | . | . | . | . | . | . | . | . | . | . | . | . | . | 0.49 (0.24; 0.99) | . |
| 1.23 (0.95;  1.59) | 0.98 (0.73;  1.31) | 1.02 (0.77;  1.34) | 0.76  (0.47;   1.24) | PVI+  Posterior±  lines | . | . | 1.06 (0.58; 1.94) | . | . | . | 0.56 (0.27; 1.14) | . | . | . | . | . | . | . |
| 1.67 (1.07;  2.61) | 1.34 (0.83;  2.16) | 1.38 (0.85;  2.25) | 1.04 (0.57  1.91) | 1.36 (0.81;  2.29) | PVI+  RDN | . | . | . | . | . | . | . | . | . | . | . | . | . |
| 0.85 (0.39;  1.87) | 0.68 (0.31;  1.53) | 0.71 (0.31;  1.59) | 0.53  (0.22;  1.29) | 0.70 (0.30;  1.59) | 0.51  (0.21;  1.26) | PVI+  LAA | . | . | . | . | . | . | . | . | . | . | . | . |
| 1.18 (0.86;  1.62) | 0.95 (0.68;  1.31) | 0.98 (0.73;   1.32) | 0.74 (0.44;  1.22) | 0.96 (0.68;  1.36) | 0.71  (0.41;  1.22) | 1.38  (0.59;  3.23) | PVI  +comb | . | 0.75 (0.33; 1.73) | . | . | . | . | . | . | . | . | . |
| 1.12 (0.73;  1.70) | 0.90 (0.59;  1.37) | 0.93 (0.61;    1.40) | 0.70 (0.39;  1.24) | 0.91 (0.57;  1.46) | 0.67  (0.36;  1.24) | 1.31  (0.54;  3.20) | 0.95  (0.58;  1.55) | PVI+  trig | . | . | . | 0.79 (0.41; 1.54) | . | . | . | . | . | . |
| 1.39 (0.93;  2.08) | 1.11 (0.72;  1.72) | 1.15 (0.74;  1.78) | 0.87 (0.49;  1.54) | 1.13 (0.71;  1.81) | 0.83  (0.45;    1.52) | 1.63 (0.67;  3.95) | 1.18 (0.74;  1.88) | 1.24 (0.70;  2.21) | PVI+  SVC±  lines | . | . | . | . | . | . | . | . | . |
| 3.16 (1.14;  8.78) | 2.54 (0.93;  6.94) | 2.62 (0.94;  7.34) | 1.97 (0.66;  5.86) | 2.58 (0.91;  7.36) | 1.90  (0.62;  5.78) | 3.71 (1.02; 13.47) | 2.68 (0.93;  7.72) | 2.83 (0.95;  8.43) | 2.28 (0.76;  6.81) | PVI+  BI-mod | . | . | . | . | . | . | . | . |
| 1.06 (0.64;  1.75) | 0.85 (0.50;  1.42) | 0.87 (0.52;  1.48) | 0.66 (0.34;  1.26) | 0.86 (0.52;  1.43) | 0.63  (0.32;  1.24) | 1.24 (0.49;  3.16) | 0.89 (0.50;  1.59) | 0.95 (0.51;  1.75) | 0.76 (0.40;  1.45) | 0.33 (0.11;  1.04) | PVI+  SUB-mod | 0.63 (0.35; 1.13) | . | . | . | . | . | . |
| 0.89 (0.65;  1.22) | 0.71 (0.51;  1.00) | 0.74 (0.52;  1.04) | 0.55 (0.33;  0.92) | 0.73 (0.50;  1.06) | 0.53 (0.31;  0.92) | 1.04 (0.45;  2.44) | 0.75 (0.49;  1.16) | 0.80 (0.51;  1.24) | 0.64 (0.38;  1.06) | 0.28 (0.10;  0.81) | 0.84 (0.52;  1.36) | PVI-  step | . | . | . | . | . | . |
| 0.88 (0.56;  1.39) | 0.71 (0.44;  1.15) | 0.73 (0.45;  1.20) | 0.55 (0.30;  1.01) | 0.72 (0.43;  1.21) | 0.53  (0.28;  1.00) | 1.04 (0.42;  2.57) | 0.75 (0.43;  1.30) | 0.79 (0.43;  1.46) | 0.64 (0.35;  1.17) | 0.28 (0.09;  0.85) | 0.84 (0.43;  1.65) | 0.99 (0.57;  1.72) | PVI partly | . | . | . | . | . |
| 1.13 (0.72;  1.76) | 0.91 (0.57;  1.44) | 0.94 (0.58;  1.51) | 0.71 (0.39;  1.29) | 0.92 (0.55;  1.54) | 0.68  (0.36;  1.27) | 1.33 (0.54;  3.28) | 0.96 (0.56;  1.64) | 1.01 (0.55;  1.86) | 0.81 (0.45;  1.48) | 0.36 (0.12;  1.08) | 1.07 (0.55;  2.10) | 1.27 (0.74;  2.19) | 1.28 (0.68;  2.41) | Single box | 1.12 (0.56; 2.23) | . | . | . |
| 1.27 (0.70;  2.29) | 1.02 (0.56;  1.84) | 1.05 (0.57;  1.94) | 0.79 (0.39;  1.61) | 1.03 (0.55;  1.96) | 0.76  (0.36;  1.59) | 1.49 (0.56;  3.98) | 1.08 (0.56;  2.08) | 1.14 (0.56;  2.32) | 0.91 (0.45;  1.86) | 0.40 (0.12;  1.29) | 1.20 (0.55;  2.60) | 1.43 (0.73;  2.77) | 1.43 (0.68;  3.01) | 1.12 (0.59;  2.13) | Single box+  lines | . | . | . |
| 0.75 (0.35;  1.61) | 0.60 (0.28;  1.31) | 0.62 (0.28;  1.36) | 0.47 (0.20;  1.11) | 0.61 (0.27;  1.37) | 0.45  (0.19;    1.09) | 0.88 (0.29;  2.63) | 0.64 (0.28;  1.45) | 0.67 (0.28;  1.60) | 0.54 (0.23;  1.28) | 0.24 (0.07;  0.85) | 0.71 (0.28;  1.77) | 0.84 (0.37;  1.92) | 0.85 (0.35;  2.05) | 0.66 (0.27;  1.60) | 0.59 (0.23;  1.55) | lines | . | . |
| 0.72 (0.45;  1.12) | 0.57 (0.36;  0.92) | 0.59 (0.36;  0.97) | 0.45 (0.26;  0.76) | 0.58 (0.35;  0.98) | 0.43  (0.23;  0.81) | 0.84 (0.34;  2.08) | 0.61 (0.35;  1.05) | 0.64 (0.35;  1.18) | 0.51 (0.28;  0.94) | 0.23 (0.07;  0.69) | 0.68 (0.34;  1.33) | 0.80 (0.46;  1.39) | 0.81 (0.43;  1.53) | 0.63 (0.34;  1.19) | 0.56 (0.27;  1.18) | 0.95 (0.39;  2.31) | GP | . |
| 0.54 (0.38;  0.77) | 0.43 (0.29;  0.64) | 0.45 (0.31;  0.65) | 0.34 (0.20;  0.58) | 0.44 (0.29;  0.68) | 0.32  (0.18;  0.57) | 0.63 (0.27;  1.50) | 0.46 (0.29;  0.72) | 0.48 (0.28;  0.82) | 0.39 (0.23;  0.66) | 0.17 (0.06;  0.50) | 0.51 (0.28;  0.94) | 0.61 (0.38;  0.97) | 0.61 (0.34;  1.08) | 0.48 (0.27;  0.84) | 0.42 (0.21;  0.84) | 0.72 (0.31;  1.67) | 0.75 (0.42;  1.34) | EGM |

NMA results (RR<1 favour the treatment in the column)

**Heterogeneity:** τ^2^= 0.084

**Table S2.** Relative risk ratios estimated from the network meta-analysis (lower triangle) and pairwise meta-analysis (upper triangle) comparing every pair of the 17 interventions with respect to safety.

| PVI | 0.89 (0.53;   1.49) | 1.18 (0.76;   1.85) | 0.34 (0.01;   8.13) | 1.06 (0.54;   2.08) | 1.04 (0.37;   2.89) | 3.07 (0.13;  73.31) | . | . | 0.66 (0.26;   1.67) | . | . | 0.48 (0.17;   1.38) | 1.15 (0.19;   6.94) | 0.14 (0.01;   2.31) | 2.34 (0.86;   6.38) |
| --- | --- | --- | --- | --- | --- | --- | --- | --- | --- | --- | --- | --- | --- | --- | --- |
| 0.84 (0.55;    1.28) | PVI+lines | 1.35 (0.73;   2.52) | 0.75 (0.17;   3.29) | 0.33 (0.04;   3.10) | . | . | . | 2.20 (0.21;  23.53) | . | 0.19 (0.01;   3.84) | . | 0.80 (0.22;   2.86) | . | . | . |
| 1.07 (0.73;    1.58) | 1.28 (0.80;    2.04) | PVI+EGM | . | 1.06 (0.16;   7.05) | . | . | 2.08 (0.68;   6.34) | 2.03 (0.36;  11.48) | . | . | . | . | . | . | 1.00 (0.15;   6.70) |
| 0.56 (0.14;    2.25) | 0.67 (0.18;    2.57) | 0.53 (0.13;    2.14) | PVI+GP | . | . | . | . | . | . | . | . | . | . | . | . |
| 1.22 (0.69;    2.16) | 1.46 (0.74;    2.86) | 1.14 (0.60;    2.16) | 2.17 (0.49;    9.54) | PVI+posterior±lines | . | . | 0.84 (0.26;   2.67) | . | . | . | 0.57 (0.18;   1.85) | . | . | . | . |
| 1.04 (0.37;    2.89) | 1.24 (0.41;    3.76) | 0.97 (0.32;    2.90) | 1.84 (0.33;   10.31) | 0.85 (0.26;    2.75) | PVI+RDN | . | . | . | . | . | . | . | . | . | . |
| 3.07 (0.13;   73.31) | 3.66 (0.15;   89.98) | 2.86 (0.12;   70.03) | 5.44 (0.17;  173.40) | 2.51 (0.10;   63.10) | 2.95 (0.11;   82.74) | PVI+LAA | . | . | . | . | . | . | . | . | . |
| 1.45 (0.62;    3.36) | 1.72 (0.70;    4.25) | 1.35 (0.59;    3.09) | 2.56 (0.52;   12.70) | 1.18 (0.51;    2.75) | 1.39 (0.37;    5.23) | 0.47 (0.02;   12.57) | PVI+comb | . | 1.00 (0.06;  15.77) | . | . | . | . | . | . |
| 0.83 (0.36;    1.94) | 1.00 (0.42;    2.37) | 0.78 (0.32;    1.87) | 1.48 (0.30;    7.22) | 0.68 (0.26;    1.78) | 0.80 (0.21;    3.02) | 0.27 (0.01;    7.26) | 0.58 (0.18;    1.83) | PVI+trig | . | . | . | 0.75 (0.42;   1.35) | . | . | . |
| 0.72 (0.30;    1.73) | 0.86 (0.32;    2.27) | 0.67 (0.26;    1.74) | 1.27 (0.25;    6.54) | 0.59 (0.21;    1.65) | 0.69 (0.18;    2.66) | 0.23 (0.01;    6.30) | 0.50 (0.16;    1.58) | 0.86 (0.26;    2.90) | PVI+SVC±lines | . | . | . | . | . | . |
| 0.16 (0.01;    3.32) | 0.19 (0.01;    3.84) | 0.15 (0.01;    3.11) | 0.28 (0.01;    7.60) | 0.13 (0.01;    2.84) | 0.15 (0.01;    3.77) | 0.05 (0.00;    4.20) | 0.11 (0.00;    2.55) | 0.19 (0.01;    4.36) | 0.22 (0.01;    5.23) | PVI+BI-mod | . | . | . | . | . |
| 0.99 (0.37;    2.69) | 1.18 (0.42;    3.36) | 0.93 (0.33;    2.61) | 1.76 (0.33;    9.48) | 0.81 (0.31;    2.10) | 0.95 (0.23;    3.98) | 0.32 (0.01;    9.01) | 0.69 (0.20;    2.32) | 1.19 (0.39;    3.63) | 1.38 (0.37;    5.18) | 6.22 (0.26;  149.73) | PVI+SUB-mod | 0.37 (0.10;   1.36) | . | . | . |
| 0.57 (0.28;    1.15) | 0.68 (0.32;    1.41) | 0.53 (0.25;    1.12) | 1.00 (0.22;    4.57) | 0.46 (0.20;    1.07) | 0.54 (0.16;    1.89) | 0.18 (0.01;    4.76) | 0.39 (0.14;    1.13) | 0.68 (0.39;    1.19) | 0.79 (0.26;    2.43) | 3.55 (0.16;   78.26) | 0.57 (0.21;    1.53) | PVI-step | . | . | . |
| 1.15 (0.19;    6.94) | 1.38 (0.22;    8.70) | 1.08 (0.17;    6.75) | 2.04 (0.21;   19.71) | 0.94 (0.14;    6.20) | 1.11 (0.14;    8.75) | 0.38 (0.01;   14.41) | 0.80 (0.11;    5.80) | 1.38 (0.19;   10.04) | 1.61 (0.22;   11.86) | 7.23 (0.21;  245.61) | 1.16 (0.15;    9.05) | 2.04 (0.30;   14.02) | PVI partly | . | . |
| 0.14 (0.01;    2.31) | 0.16 (0.01;    2.84) | 0.13 (0.01;    2.21) | 0.24 (0.01;    5.63) | 0.11 (0.01;    2.00) | 0.13 (0.01;    2.65) | 0.04 (0.00;    3.11) | 0.09 (0.00;    1.80) | 0.16 (0.01;    3.12) | 0.19 (0.01;    3.67) | 0.85 (0.01;   54.04) | 0.14 (0.01;    2.75) | 0.24 (0.01;    4.44) | 0.12 (0.00;    3.37) | Single box | . |
| 2.13 (0.80;    5.71) | 2.55 (0.88;    7.39) | 1.99 (0.70;    5.63) | 3.78 (0.70;   20.59) | 1.75 (0.56;    5.42) | 2.05 (0.50;    8.48) | 0.70 (0.03;   19.30) | 1.48 (0.41;    5.35) | 2.56 (0.70;    9.30) | 2.97 (0.80;   11.12) | 13.38 (0.55;  324.31) | 2.15 (0.53;    8.69) | 3.77 (1.13;   12.61) | 1.85 (0.24;   14.34) | 15.72 (0.78;  315.03) | EGM |

NMA results (RR<1 favour the treatment in the column)

**Heterogeneity:** τ^2^=0.00

**Table S3.** Relative risk ratios estimated from the network meta-analysis (lower triangle) and pairwise meta-analysis (upper triangle) comparing every pair of the 18 interventions with respect to procedural time.

| PVI | -0.36 (-0.58; -0.14) | -0.82 (-1.06; -0.59) | -0.35 (-0.97;  0.28) | -0.43 (-0.70; -0.15) | -0.63 (-1.35;  0.09) | -0.63 (-1.26; -0.01) | . | . | -0.19 (-0.50;  0.13) | . | . | -0.82 (-1.20; -0.44) | 0.68 ( 0.27;  1.09) | -0.09 (-0.69;  0.51) | -0.27 (-0.98;  0.45) | -0.25 (-0.93;  0.43) | -0.40 (-0.82;  0.02) |
| --- | --- | --- | --- | --- | --- | --- | --- | --- | --- | --- | --- | --- | --- | --- | --- | --- | --- |
| -0.32 (-0.50; -0.13) | PVI+  lines | -0.20 (-0.56;  0.16) | -0.65 (-1.26; -0.04) | -0.20 (-0.85;  0.45) | . | . | -0.82 (-1.45; -0.18) | 0.08 (-0.75;  0.92) | . | -1.07 (-1.70; -0.44) | . | -0.68 (-1.32; -0.05) | . | 0.17 (-0.58;  0.92) | 0.12 (-0.62;  0.85) | . | . |
| -0.76 (-0.95; -0.57) | -0.44 (-0.67; -0.22) | PVI+  EGM | . | 0.80 ( 0.22;  1.39) | . | . | 0.01 (-0.52;  0.54) | 0.41 (-0.19;  1.01) | . | . | . | . | . | . | . | . | 0.38 (-0.13;  0.90) |
| -0.67 (-1.11; -0.22) | -0.35 (-0.79;  0.10) | 0.09 (-0.38;  0.57) | PVI+  GP | . | . | . | . | . | . | . | . | . | . | . | . | . | . |
| -0.33 (-0.57; -0.10) | -0.02 (-0.29;  0.26) | 0.43 ( 0.15;  0.70) | 0.33 (-0.16;  0.83) | PVI+  posterior±  lines | . | . | -0.42 (-1.12;  0.27) | . | . | . | -0.05 (-0.70;  0.60) | . | . | . | . | . | . |
| -0.63 (-1.35;  0.09) | -0.32 (-1.06;  0.43) | 0.13 (-0.62;  0.87) | 0.03 (-0.81;  0.88) | -0.30 (-1.06;  0.46) | PVI+  RDN | . | . | . | . | . | . | . | . | . | . | . | . |
| -0.63 (-1.26; -0.01) | -0.32 (-0.96;  0.33) | 0.13 (-0.52;  0.78) | 0.03 (-0.73;  0.80) | -0.30 (-0.96;  0.37) | -0.00 (-0.95;  0.95) | PVI+  LAA | . | . | . | . | . | . | . | . | . | . | . |
| -0.82 (-1.15; -0.49) | -0.50 (-0.85; -0.16) | -0.06 (-0.40;  0.28) | -0.15 (-0.70;  0.39) | -0.49 (-0.85; -0.12) | -0.19 (-0.98;  0.61) | -0.19 (-0.89;  0.52) | PVI+  comb | . | 0.43 (-0.19;  1.06) | . | . | . | . | . | . | . | . |
| -0.46 (-0.90; -0.02) | -0.14 (-0.59;  0.31) | 0.30 (-0.14;  0.74) | 0.21 (-0.41;  0.83) | -0.13 (-0.61;  0.36) | 0.17 (-0.67;  1.02) | 0.17 (-0.59;  0.94) | 0.36 (-0.17;  0.89) | PVI+  trig | . | . | . | -0.23 (-0.86;  0.39) | . | . | . | . | . |
| -0.23 (-0.52;  0.06) | 0.09 (-0.25;  0.42) | 0.53 ( 0.19;  0.87) | 0.44 (-0.09;  0.97) | 0.11 (-0.26;  0.47) | 0.40 (-0.37;  1.18) | 0.40 (-0.28;  1.09) | 0.59 ( 0.20;  0.98) | 0.23 (-0.30;  0.76) | PVI+  SVC±  lines | . | . | . | . | . | . | . | . |
| -1.38 (-2.04; -0.73) | -1.07 (-1.70; -0.44) | -0.62 (-1.29;  0.05) | -0.72 (-1.49;  0.05) | -1.05 (-1.74; -0.36) | -0.75 (-1.73;  0.22) | -0.75 (-1.66;  0.15) | -0.56 (-1.28;  0.15) | -0.92 (-1.70; -0.15) | -1.16 (-1.87; -0.44) | PVI+  BI-mod | . | . | . | . | . | . | . |
| -0.16 (-0.59;  0.26) | 0.15 (-0.29;  0.60) | 0.60 ( 0.14;  1.05) | 0.50 (-0.11;  1.11) | 0.17 (-0.26;  0.60) | 0.47 (-0.37;  1.31) | 0.47 (-0.28;  1.22) | 0.66 ( 0.13;  1.18) | 0.30 (-0.27;  0.86) | 0.06 (-0.45;  0.58) | 1.22 ( 0.45;  1.99) | PVI+  SUB-mod | -0.81 (-1.24; -0.37) | . | . | . | . | . |
| -0.87 (-1.16; -0.59) | -0.56 (-0.87; -0.24) | -0.11 (-0.44;  0.21) | -0.21 (-0.73;  0.31) | -0.54 (-0.89; -0.19) | -0.24 (-1.02;  0.54) | -0.24 (-0.93;  0.44) | -0.05 (-0.48;  0.37) | -0.41 (-0.86;  0.03) | -0.65 (-1.05; -0.24) | 0.51 (-0.19;  1.21) | -0.71 (-1.09; -0.33) | PVI-step | . | . | . | . | . |
| 0.68 ( 0.27;  1.09) | 1.00 ( 0.55;  1.44) | 1.44 ( 0.99;  1.89) | 1.35 ( 0.74;  1.95) | 1.01 ( 0.55;  1.48) | 1.31 ( 0.49;  2.14) | 1.31 ( 0.57;  2.06) | 1.50 ( 0.98;  2.03) | 1.14 ( 0.54;  1.74) | 0.91 ( 0.41;  1.41) | 2.06 ( 1.29;  2.84) | 0.84 ( 0.26;  1.43) | 1.55 ( 1.06;  2.05) | PVI partly | . | . | . | . |
| -0.08 (-0.64;  0.47) | 0.23 (-0.33;  0.80) | 0.67 ( 0.09;  1.26) | 0.58 (-0.12;  1.29) | 0.25 (-0.35;  0.85) | 0.55 (-0.36;  1.46) | 0.55 (-0.29;  1.38) | 0.74 ( 0.10;  1.38) | 0.38 (-0.33;  1.08) | 0.14 (-0.48;  0.77) | 1.30 ( 0.45;  2.15) | 0.08 (-0.62;  0.77) | 0.79 ( 0.17;  1.41) | -0.76 (-1.45; -0.08) | Single box | -0.05 (-0.81;  0.71) | . | . |
| -0.21 (-0.84;  0.42) | 0.11 (-0.52;  0.75) | 0.55 (-0.10;  1.21) | 0.46 (-0.30;  1.22) | 0.13 (-0.54;  0.80) | 0.43 (-0.53;  1.38) | 0.43 (-0.46;  1.31) | 0.61 (-0.09;  1.32) | 0.25 (-0.51;  1.02) | 0.02 (-0.67;  0.71) | 1.18 ( 0.28;  2.07) | -0.04 (-0.80;  0.71) | 0.67 (-0.02;  1.36) | -0.89 (-1.64; -0.14) | -0.12 (-0.84;  0.60) | Single box+  lines | . | . |
| -0.25 (-0.93;  0.43) | 0.07 (-0.63;  0.77) | 0.51 (-0.19;  1.21) | 0.42 (-0.39;  1.23) | 0.08 (-0.63;  0.80) | 0.38 (-0.60;  1.37) | 0.38 (-0.54;  1.30) | 0.57 (-0.18;  1.32) | 0.21 (-0.60;  1.02) | -0.02 (-0.76;  0.71) | 1.13 ( 0.19;  2.08) | -0.09 (-0.88;  0.71) | 0.62 (-0.11;  1.36) | -0.93 (-1.72; -0.14) | -0.17 (-1.04;  0.71) | -0.04 (-0.97;  0.88) | lines | . |
| -0.38 (-0.77;  0.01) | -0.06 (-0.48;  0.36) | 0.38 (-0.02;  0.78) | 0.29 (-0.30;  0.88) | -0.04 (-0.49;  0.40) | 0.26 (-0.56;  1.07) | 0.26 (-0.48;  0.99) | 0.44 (-0.06;  0.94) | 0.08 (-0.50;  0.66) | -0.15 (-0.63;  0.33) | 1.01 ( 0.25;  1.76) | -0.21 (-0.78;  0.36) | 0.50 ( 0.02;  0.98) | -1.06 (-1.62; -0.50) | -0.29 (-0.97;  0.38) | -0.17 (-0.91;  0.57) | -0.13 (-0.91;  0.65) | EGM |

MD (hours scale) <0 favours the treatment in the column

**Heterogeneity:** τ^2^=0.092

**Figure S1**: P-scores for the two primary outcomes

**
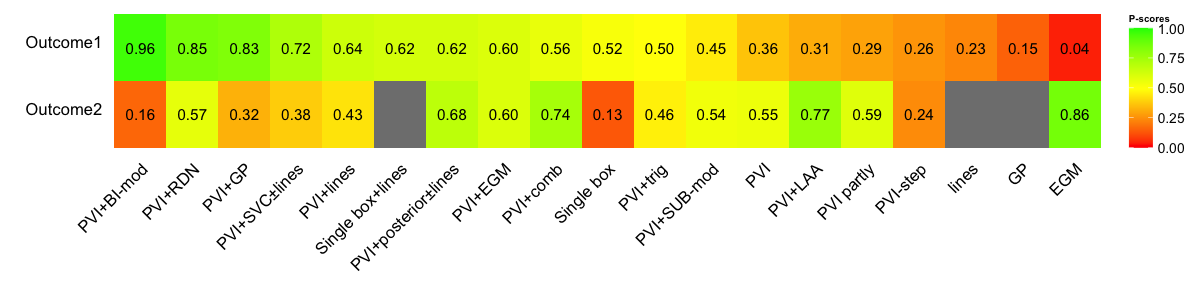
**

Abbreviations: Outcome 1: recurrence, Outcome 2: Safety.

The interpretation of the ranking of strategies concerning the safety outcome (outcome 2), according to the p-scores, must be treated with caution due to the high uncertainty
