# Supplementary material for: Comparing efficacy and safety in catheter ablation strategies for atrial fibrillation: a network meta-analysis
Source: BMC Med. 2022 May 31;20:193. doi: 10.1186/s12916-022-02385-2 (PMC9153169; doi:10.1186/s12916-022-02385-2)
Supplement: Supplementary file 8 — Additional file 8. Results from component network meta-analysis. Figures S1-S4. Figure S1- [Network plots from CNMA model for efficacy (a), safety (b) and procedural time (c). Each treatment is represented as a node and an edge exists between two nodes if direct trial evidence is available. The size of each node is proportional to the number of patients involved in each treatment across all trials, while the size of the edges is proportional to the number of studies available in the corresponding comparison]. Figure S2- [Component network forest plots of relative risk ratios for efficacy]. Figure S3- [Component network forest plots of relative risk ratios for safety]. Figure S4- [Component network forest plots of relative risk ratios for procedural time]. [file 12916_2022_2385_MOESM8_ESM.docx]

**Additional file 8. RESULTS FROM COMPONENT NETWORK META-ANALYSIS**

Component network meta-analysis (CNMA) uses the information that some of the treatments included in the network are a combination of others into the model. Additive component network meta-analysis separates effects for each component within the intervention and assumes that the effects of combined treatments are the sum of the single component effects.

eFigure4 reports the component network diagrams for the three outcomes of interest, which contains additional links of strategies composed of treatment combinations and their single components. eFigure5, eFigure6, eFigure7 report the forest plots of both NMA and CNMA results for comparison, respectively for efficacy, safety, and procedural time. For each outcome, the biggest difference concerning the standard NMA model was found in the comparison of EGM vs. PVI. For example, for efficacy, the additive CNMA model estimates a risk ratio of 0.97 (CI: 0.80,1.17) whereas the standard NMA model has a significant RR of 1.87 (CI:1.31,2.68). Changes in RR in the additive CNMA and standard NMA models were also observed for the following comparisons: GP vs. PVI, lines vs. PVI, PVI+GP vs. PVI, however any of these differences were statistically significant. Overall, we can conclude that the standard NMA and the CNMA model led to similar results, suggesting that no specific singular component is driving the total effect of treatments used in combination.

**Figure S1.** Network plots from CNMA model for efficacy (a), safety (b) and procedural time (c). Each treatment is represented as a node and an edge exists between two nodes if direct trial evidence is available. The size of each node is proportional to the number of patients involved in each treatment across all trials, while the size of the edges is proportional to the number of studies available in the corresponding comparison.

| 1. 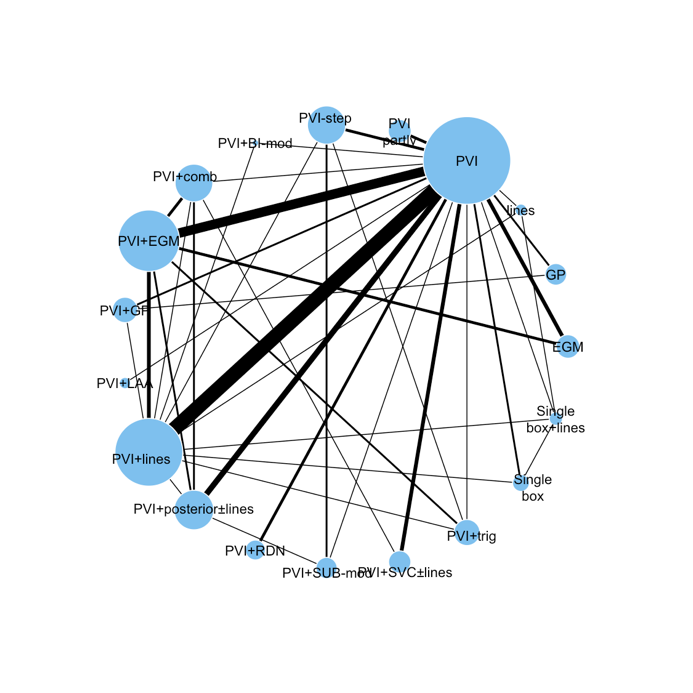efficacy | 1. 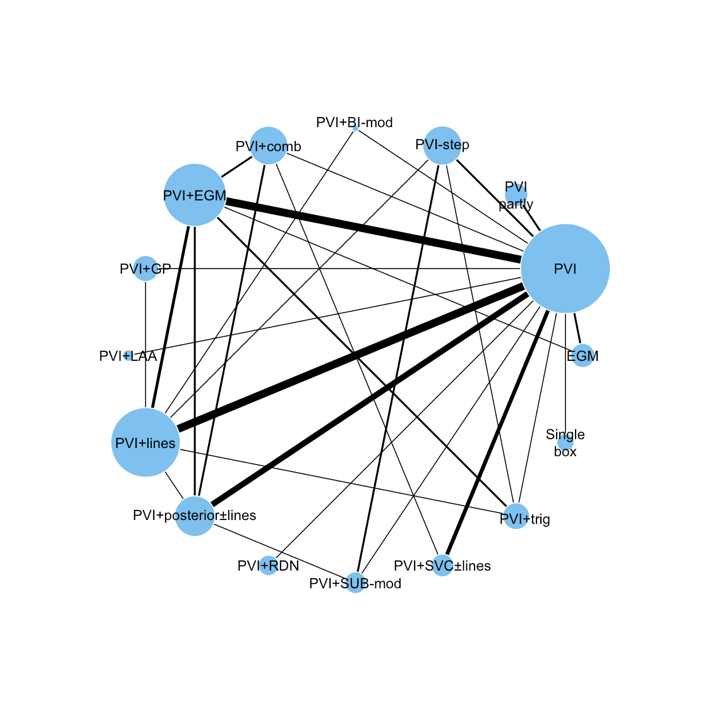safety |
| --- | --- |
| 1. **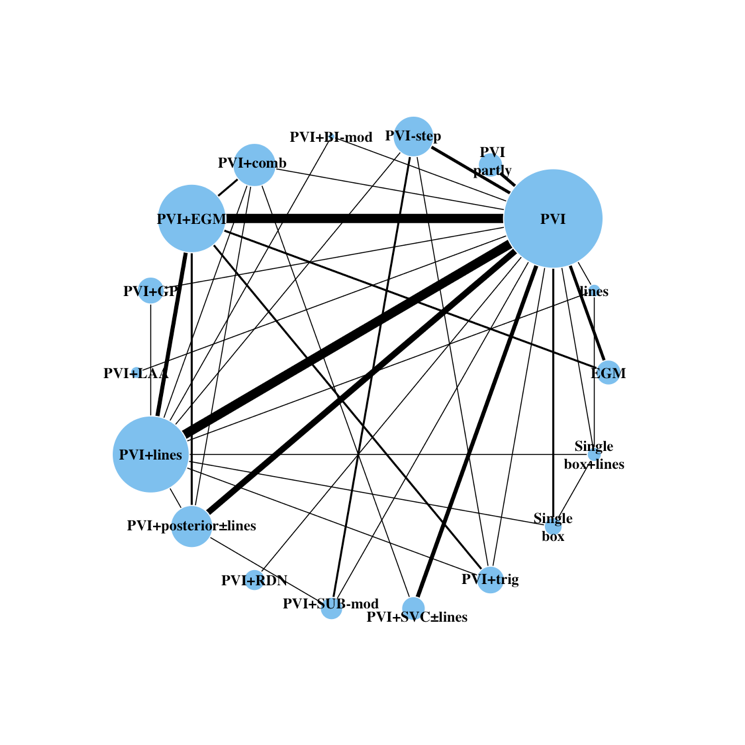**procedural   time | |

**Figure S2.** Component network forest plots of relative risk ratios for efficacy.

**
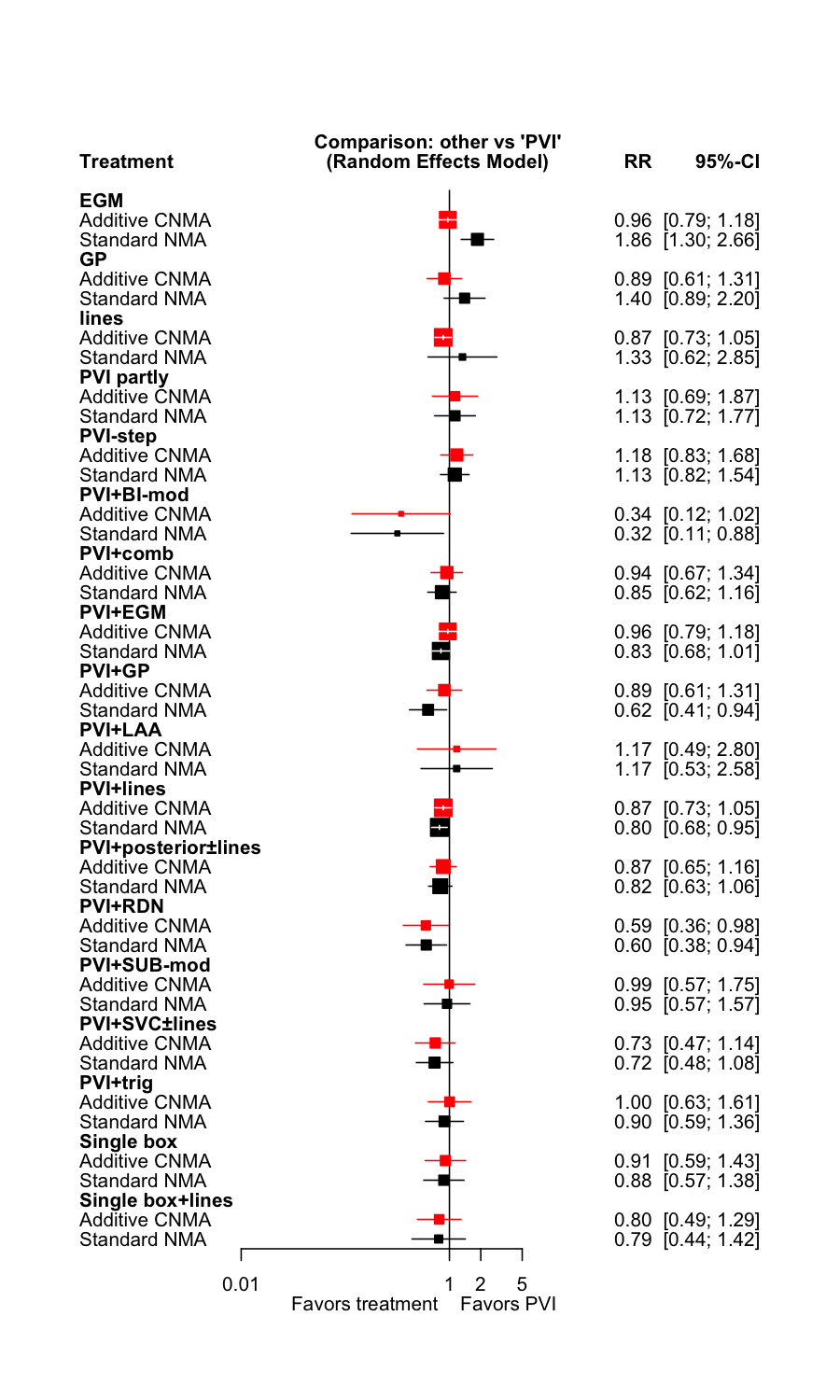
**

**Heterogeneity:** $\tau^{2}=0.12$

**Figure S3.** Component network forest plots of relative risk ratios for safety.

**
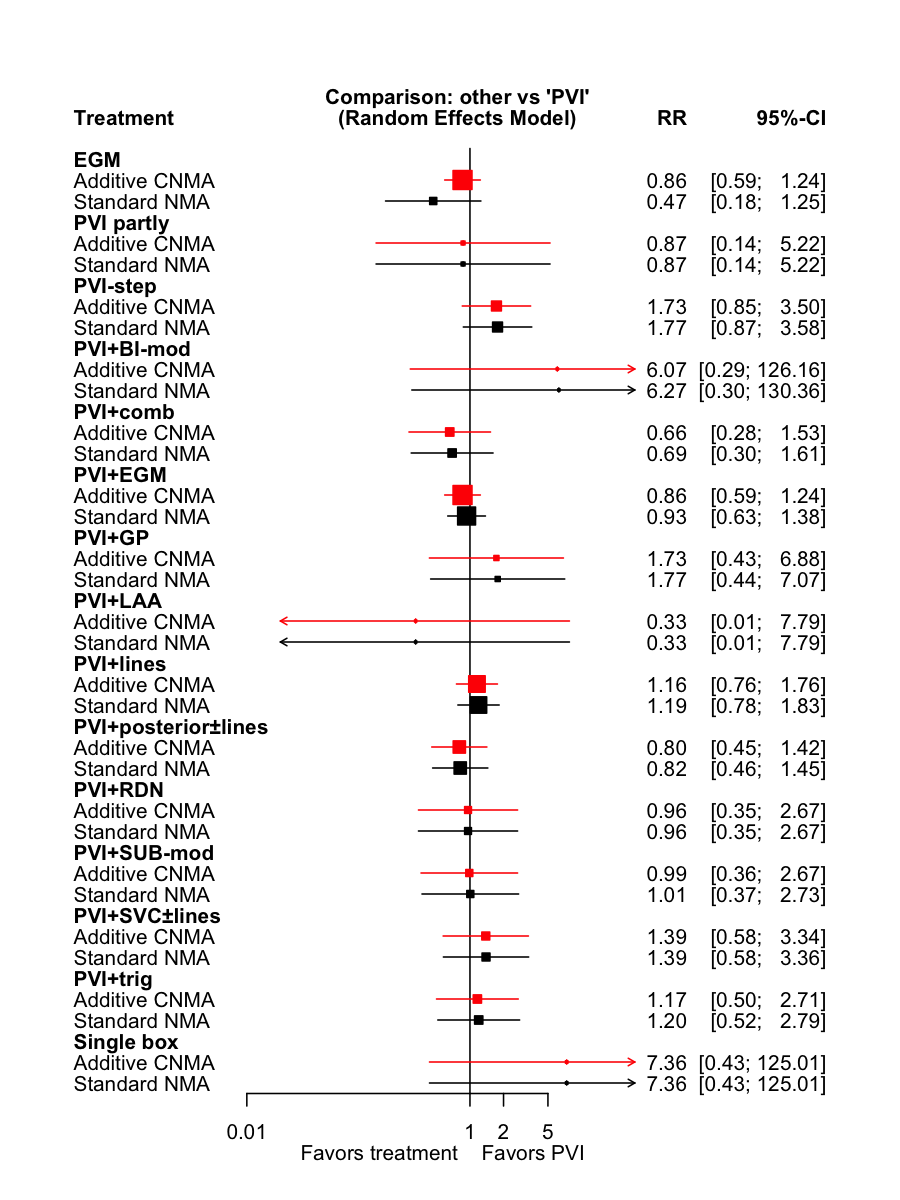
**

**Heterogeneity:** $\tau^{2}=0$.00

**Figure S4.** Component network forest plots of relative risk ratios for procedural time.

**
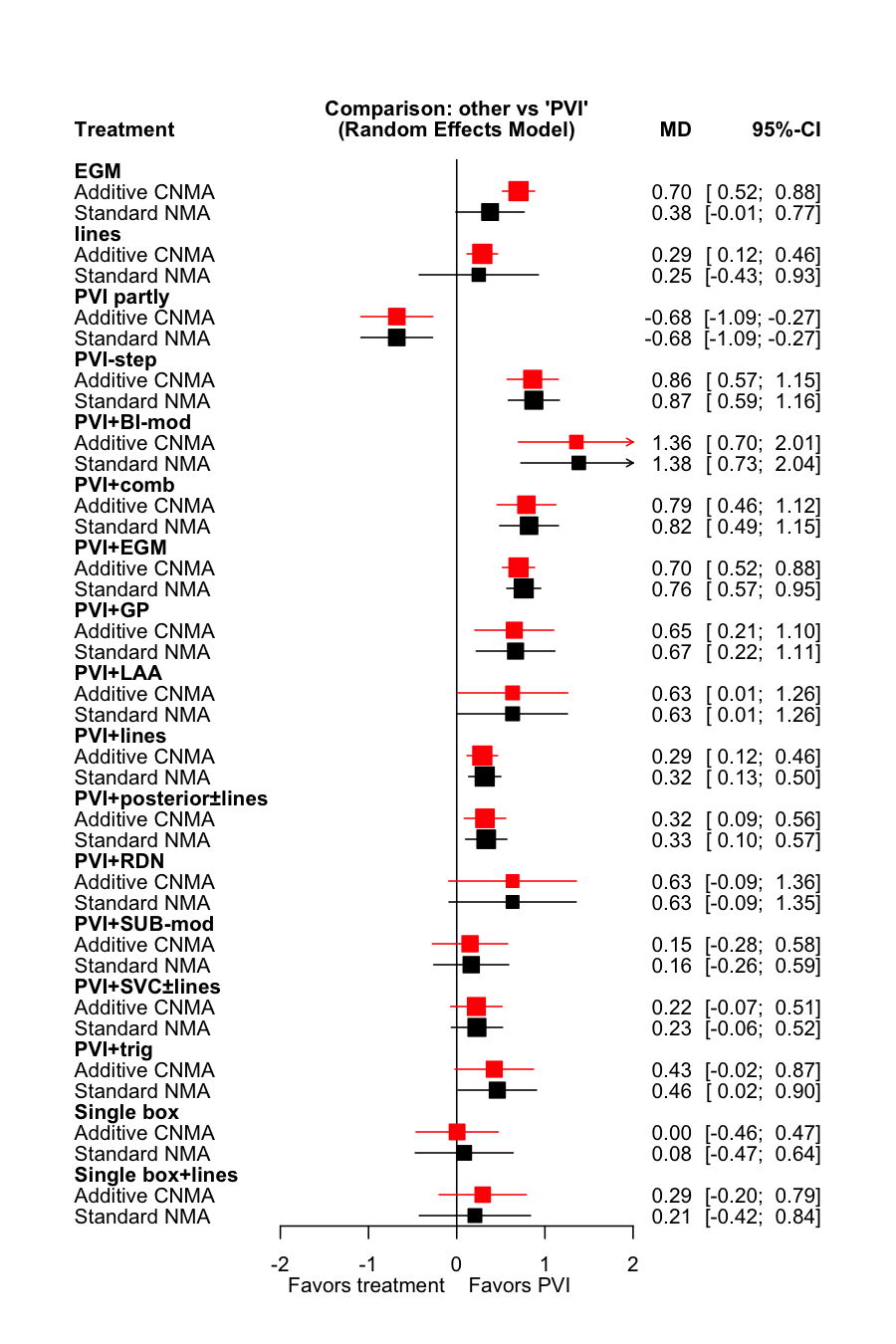
**

$\mathbf{Heterogeneity:}\tau^{2}=$0.09
