# Supplementary material for: Comparing efficacy and safety in catheter ablation strategies for atrial fibrillation: a network meta-analysis
Source: BMC Med. 2022 May 31;20:193. doi: 10.1186/s12916-022-02385-2 (PMC9153169; doi:10.1186/s12916-022-02385-2)
Supplement: Supplementary file 9 — Additional file 9. Evaluation of inconsistency. Tables S1-S2, Figures S1-S3. Table S1- [Design-by-treatment interaction test, with global p-value, Q statistic and degrees of freedom for each outcome]. Table S2- [Results of the inconsistency net-split approach for all outcomes. For each comparison the direct and indirect estimates are provided along with the respective z-values and p-values of differences. P-values<0.10 indicate significant disagreement between direct and indirect evidence (in red)]. Figure S1- [Forest plots of the net-split approach separating direct and indirect evidence for efficacy]. Figure S2- [Forest plots of the net-split approach separating direct and indirect evidence for safety]. Figure S3- [Forest plots of the net-split approach separating direct and indirect evidence for procedural time]. [file 12916_2022_2385_MOESM9_ESM.docx]

**Appendix 9.** **EVALUATION OF INCONSISTENCY**

Consistency means that direct evidence in a network for the effect size between two treatments A and treatment B **does not statistically differ from the indirect evidence for that same comparison**. The presence of inconsistency in the network has been evaluated with the global design-by-treatment interaction model and with the net-split approach.

The design-by-treatment interaction model is a general model for the inconsistency that provides a single inference test, based on the χ^2^ test, about the plausibility of assuming consistency for the entire network. However, inconsistency models may have low power and may sometimes fail to detect inconsistency.

The net-split approach splits the network estimates into the contribution of direct and indirect evidence, which allows us to check for inconsistency in specific comparisons in our network.

**Table S1.** Design-by-treatment interaction test, with global p-value, Q statistic and degrees of freedom for each outcome.

| **Outcome** | **p-value** | **Q (df)** |
| --- | --- | --- |
| Efficacy | 0.8411 | 16.32 (23) |
| Safety | 0.9610 | 8.95 (18) |
| Procedural time | 0.8547 | 14.34 (21) |

**Table S2.** Results of the inconsistency net-split approach for all outcomes. For each comparison the direct and indirect estimates are provided along with the respective z-values and p-values of differences. P-values<0.10 indicate significant disagreement between direct and indirect evidence (in red).

|  | **Comparisons** | **NMA** | **direct** | **indirect** | **z** | **p-value** |
| --- | --- | --- | --- | --- | --- | --- |
| Efficacy | EGM vs PVI  EGM vs PVI+EGM  GP vs PVI  GP vs PVI+GP  PVI-step vs PVI  PVI+EGM vs PVI  PVI+GP vs PVI  PVI+lines vs PVI  PVI+posterior±lines vs PVI  PVI+SVC±lines vs PVI  Single box vs PVI  Single box+lines vs PVI  PVI-step vs PVI+lines  PVI-step vs PVI+SUB-mod  PVI-step vs PVI+trig  PVI+comb vs PVI+EGM  PVI+comb vs PVI+lines  PVI+comb vs PVI+posterior±lines  PVI+comb vs PVI+SVC±lines  PVI+EGM vs PVI+lines  PVI+EGM vs PVI+posterior±lines  PVI+EGM vs PVI+trig  PVI+GP vs PVI+lines  PVI+lines vs PVI+posterior±lines  PVI+lines vs PVI+trig  PVI+lines vs Single box  PVI+lines vs Single box+lines  PVI+posterior±lines vs PVI+SUB-mod  Single box vs Single box+lines | 1.8588  2.2415  1.3985  2.2414  1.1251  0.8289  0.6239  0.8018  0.8161  0.7200  0.8842  0.7885  1.4032  1.1875  1.2564  1.0228  1.0573  1.0389  1.1775  1.0337  1.0157  0.9256  0.7781  0.9826  0.8954  0.9069  1.0169  0.8612  1.1214 | 1.6969  2.8640  1.4061  2.0476  1.0217  0.8141  0.5426  0.8749  0.8458  0.6302  0.9098  0.8333  1.2083  1.5914  1.2587  1.1138  1.2500  0.9412  0.7500  1.2171  0.8558  1.0181  0.8714  1.2857  0.8250  0.9643  1.0800  0.5588  1.1200 | 2.6900  1.6744  1.3178  2.5327  1.2848  0.8530  0.7415  0.6438  0.7657  1.2149  0.7250  0.6693  1.4634  0.6634  1.2546  0.9123  1.0051  1.0917  1.4459  0.9116  1.1140  0.7772  0.7087  0.9618  0.9376  0.8614  0.8600  1.3405  1.1301 | -1.0001  1.4004  0.0781  -0.3830  -0.7034  -0.2302  -0.7406  1.5747  0.3627  -1.2796  0.3312  0.3163  -0.4641  1.6911  0.0072  0.6505  0.5569  -0.3943  -1.2796  1.3012  -0.8811  0.6157  0.4846  0.5103  -0.2846  0.2397  0.3312  -1.6911  -0.0095 | 0.3173  0.1614  0.9325  0.7013  0.4818  0.8179  0.4589  0.1153  0.7168  0.2007  0.7405  0.7517  0.6426  **0.0908**  0.9942  0.5153  0.5776  0.6933  0.2007  0.1932  0.3782  0.5381  0.6280  0.6099  0.7759  0.8106  0.7405  **0.0908**  0.9925 |
| Safety | EGM vs PVI  EGM vs PVI+EGM  PVI-step vs PVI  PVI+EGM vs PVI  PVI+GP vs PVI  PVI+lines vs PVI  PVI+posterior±lines vs PVI  PVI+SVC±lines vs PVI  PVI-step vs PVI+lines  PVI-step vs PVI+SUB-mod  PVI-step vs PVI+trig  PVI+comb vs PVI+EGM  PVI+comb vs PVI+posterior±lines  PVI+comb vs PVI+SVC±lines  PVI+EGM vs PVI+lines  PVI+EGM vs PVI+posterior±lines  PVI+EGM vs PVI+trig  PVI+GP vs PVI+lines  PVI+lines vs PVI+posterior±lines  PVI+lines vs PVI+trig  PVI+posterior±lines vs PVI+SUB-mod | 0..4685  0.5022  1.7672  0.9329  1.7726  1.1931  0.8182  1.3930  1.4812  1.7548  1.4748  0.7418  0.8458  0.4968  0.7819  1.1402  0.7786  1.4857  1.4582  0.9957  0.8125 | 0.4273  1.0000  2.0711  0.8450  2.9130  1.1249  0.9409  1.5067  1.2500  2.7216  1.3333  0.4810  1.1897  1.0000  0.7385  1.0568  2.0301  1.3333  0.3333  2.2000  0.5714 | 4.7064  0.3744  1.5513  1.2635  1.5763  1.3458  0.5669  0.6442  1.6131  0.9789  4.0646  1.2673  0.5710  0.4275  0.8401  1.1513  0.5613  2.4640  1.6918  0.8807  1.5888 | -0.9177  0.8479  0.3991  -0.8733  0.3420  -0.3877  0.7792  0.5479  -0.3200  1.0013  -1.1223  -1.1380  0.8497  0.5479  -0.2703  -0.0833  1.2558  -0.3420  -1.3607  0.7046  -1.0013 | 0.3588  0.3965  0.6898  0.3825  0.7323  0.6983  0.4358  0.5837  0.7490  0.3167  0.2618  0.2551  0.3955  0.5837  0.7870  0.9336  0.2092  0.7323  0.1736  0.4810  0.3167 |
| Procedural  time | EGM vs PVI  EGM vs PVI+EGM  PVI-step vs PVI 3  PVI+EGM vs PVI  PVI+GP vs PVI  PVI+lines vs PVI  PVI+posterior±lines vs PVI  PVI+SVC±lines vs PVI  Single box vs PVI  Single box+lines vs PVI  PVI-step vs PVI+lines  PVI-step vs PVI+SUB-mod  PVI-step vs PVI+trig  PVI+comb vs PVI+EGM  PVI+comb vs PVI+lines  PVI+comb vs PVI+posterior±lines  PVI+comb vs PVI+SVC±lines  PVI+EGM vs PVI+lines  PVI+EGM vs PVI+posterior±lines  PVI+EGM vs PVI+trig  PVI+GP vs PVI+lines  PVI+lines vs PVI+posterior±lines  PVI+lines vs PVI+trig  PVI+lines vs Single box  PVI+lines vs Single box+lines  PVI+posterior±lines vs PVI+SUB-mod  Single box vs Single box+lines | 0.3780  -0.3819  0.8746  0.7599  0.6670  0.3178  0.3344  0.2291  0.0850  0.2063  0.5568  0.7103  0.4145  0.0607  0.5028  0.4862  0.5914  0.4421  0.4255  0.2997  0.3492  -0.0166  -0.1424  0.2328  0.1115  0.1700  0.1213 | 0.4010  -0.3817  0.8216  0.8228  0.3467  0.3589  0.4265  0.1887  0.0880  0.2667  0.6833  0.8074  0.2350  -0.0056  0.8167  0.4233  0.4333  0.2004  0.8039  0.4098  0.6500  -0.2012  0.0833  0.1667  0.1167  -0.0500  -0.0500 | 0.2509  -0.3823  0.9495  0.6326  0.9964  0.2337  0.0968  0.4454  0.0660  -0.0091  0.5161  0.4136  0.6009  0.1058  0.3732  0.5107  0.6901  0.6031  0.3163  0.1659  0.0002  0.0238  -0.2355  0.3197  0.0969  0.3437  -0.7706 | 0.2737  0.0016  -0.4274  0.9154  -1.4285  0.6295  1.2382  -0.6326  0.0268  0.3547  0.4487  0.8814  -0.8000  -0.3175  1.1467  -0.2092  -0.6326  -1.7156  1.4433  0.5367  1.4285  -0.6124  0.6292  -0.2632  0.0268  -0.8814  0.5874 | 0.7843  0.9987  0.6691  0.3600  0.1531  0.5290  0.2156  0.5270  0.9786  0.7228  0.6537  0.3781  0.4237  0.7509  0.2515  0.8343  0.5270  **0.0862**  0.1489  0.5914  0.1531  0.5402  0.5292  0.7924  0.9786  0.3781  0.5569 |

**Figure S1.** Forest plots of the net-split approach separating direct and indirect evidence for efficacy.

**
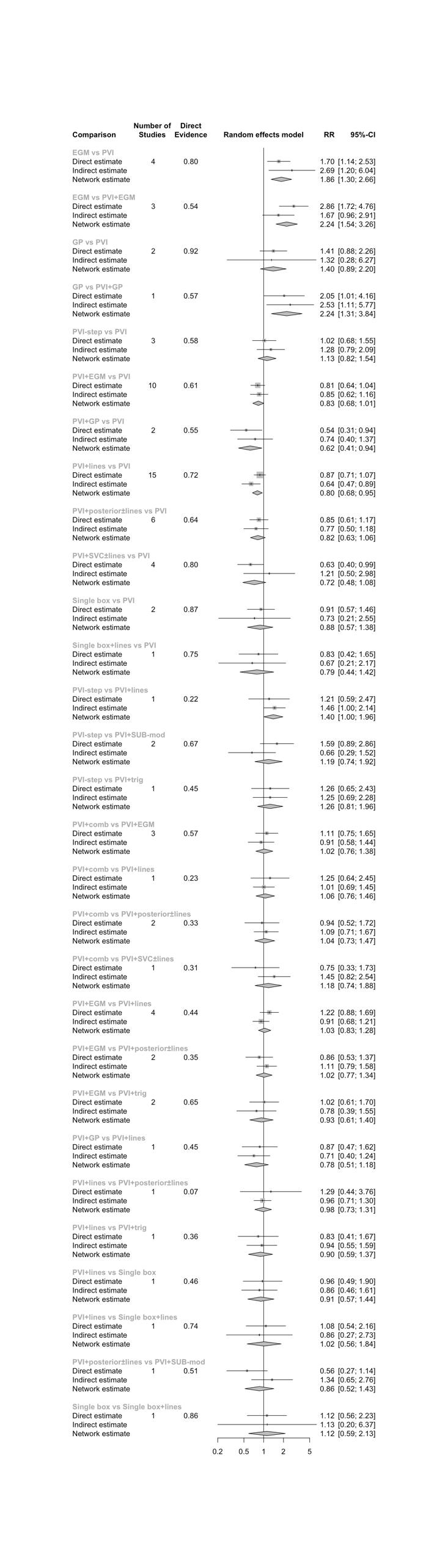
**

**Figure S2.** Forest plots of the net-split approach separating direct and indirect evidence for safety.


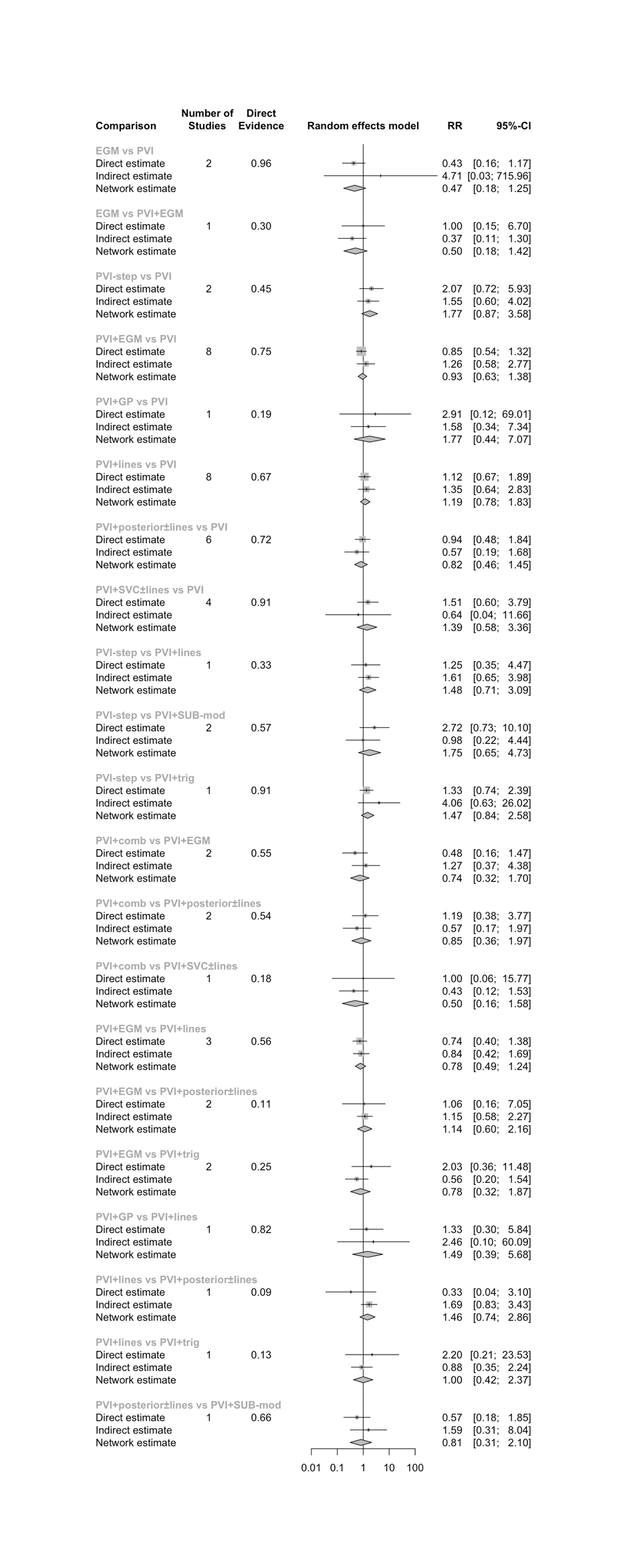


**Figure S3.** Forest plots of the net-split approach separating direct and indirect evidence for procedural time.

**
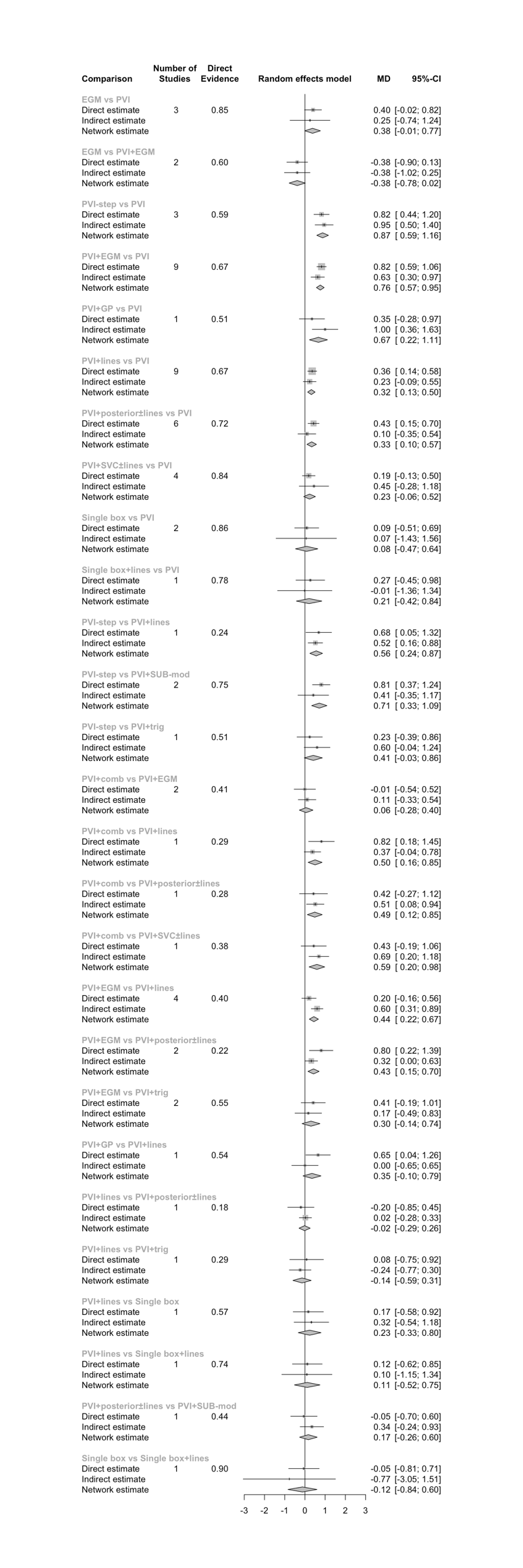
**
