# Supplementary material for: Comparing efficacy and safety in catheter ablation strategies for atrial fibrillation: a network meta-analysis
Source: BMC Med. 2022 May 31;20:193. doi: 10.1186/s12916-022-02385-2 (PMC9153169; doi:10.1186/s12916-022-02385-2)
Supplement: Supplementary file 10 — Additional file 10. Investigation of small-study effects. Figures S1-S3. Figure S1- [Comparison-adjusted funnel plot for efficacy]. Figure S2- [Comparison-adjusted funnel plot for safety]. Figure S3- [Comparison-adjusted funnel plot for procedural time]. [file 12916_2022_2385_MOESM10_ESM.docx]

**Additional file 10. INVESTIGATION OF SMALL-STUDY EFFECTS**

Comparison-adjusted funnel plots were used to investigate the presence of small-study effects, meaning important differences in treatment effect estimates between more precise and less precise studies. eFigure1, eFigure3 and eFigure3 depict the comparison-adjusted funnel plots for each outcome. The horizontal axis represents the difference between the study effect and the summary effect for each comparison, while on the vertical axis is the standard error of the observed effect size, in reversed order so that precise studies will lie at the top of the plot. Potential asymmetry indicates concerns for small-study effects.

We used comparison-adjusted funnel plots for all active strategies against control (PVI), which appeared quite symmetric around the line of no effect, suggesting the absence of small-study effects.

**Figure S1.** Comparison-adjusted funnel plot for efficacy.


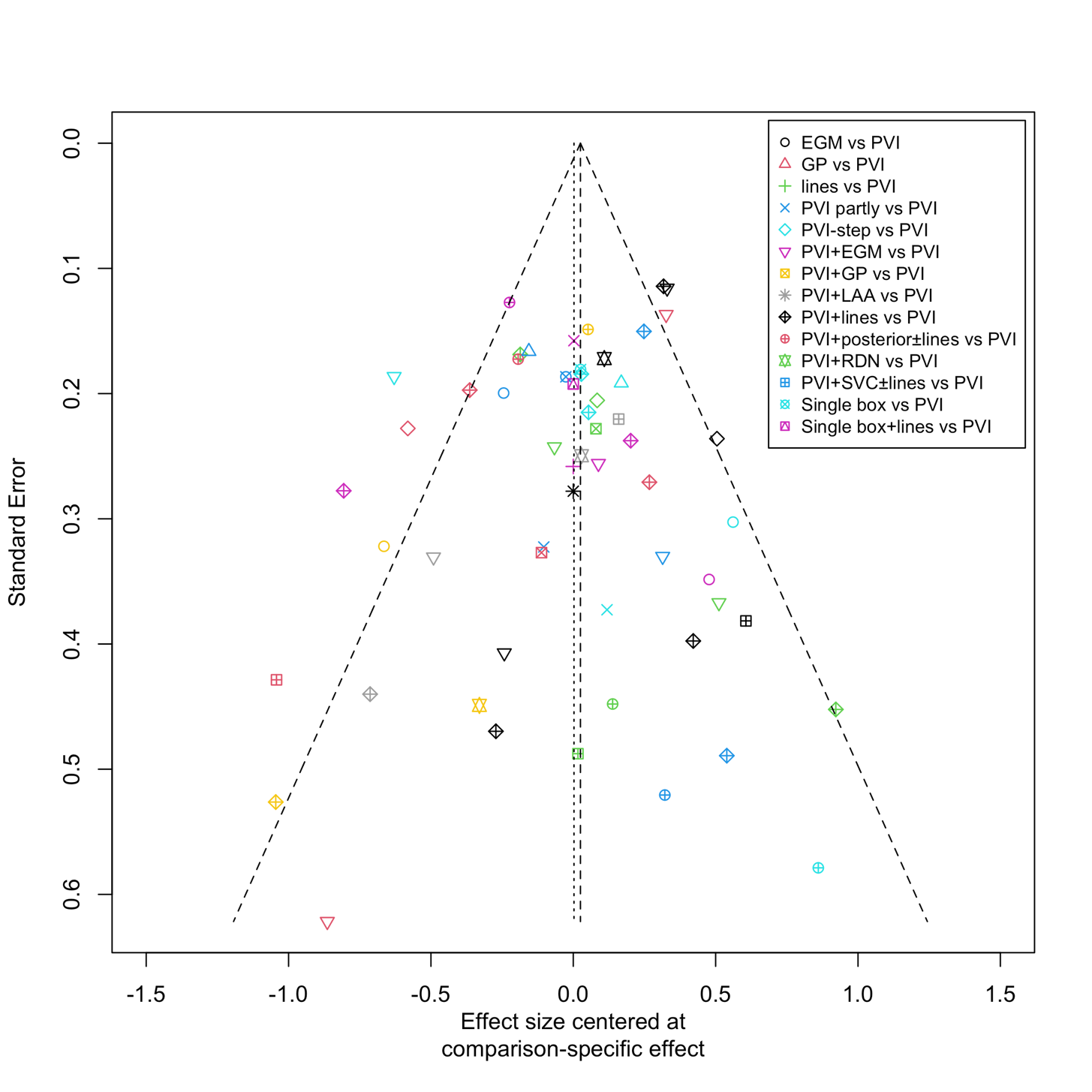


**Figure S2.** Comparison-adjusted funnel plot for safety.

**
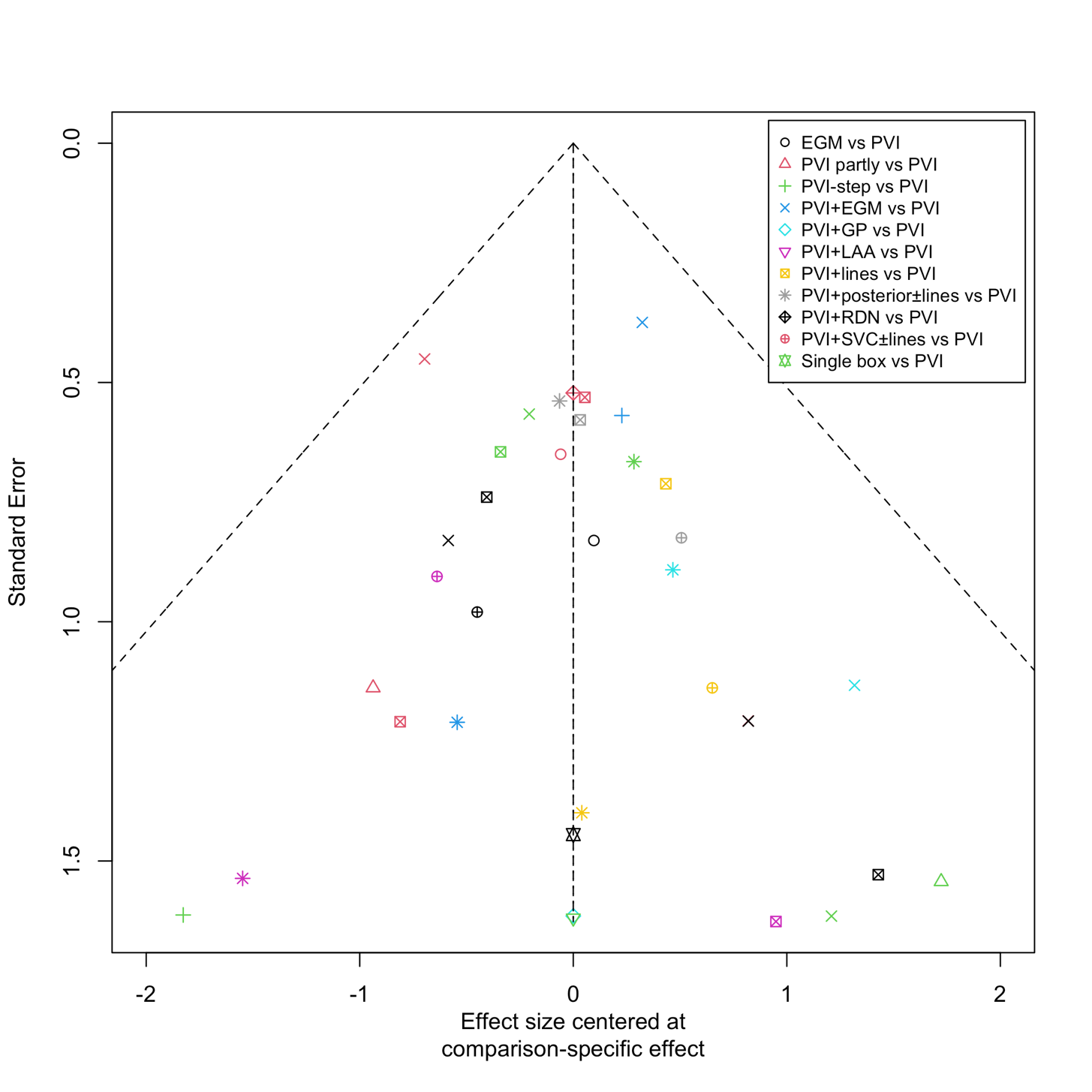
**

**Figure S3.** Comparison-adjusted funnel plot for procedural time.

**
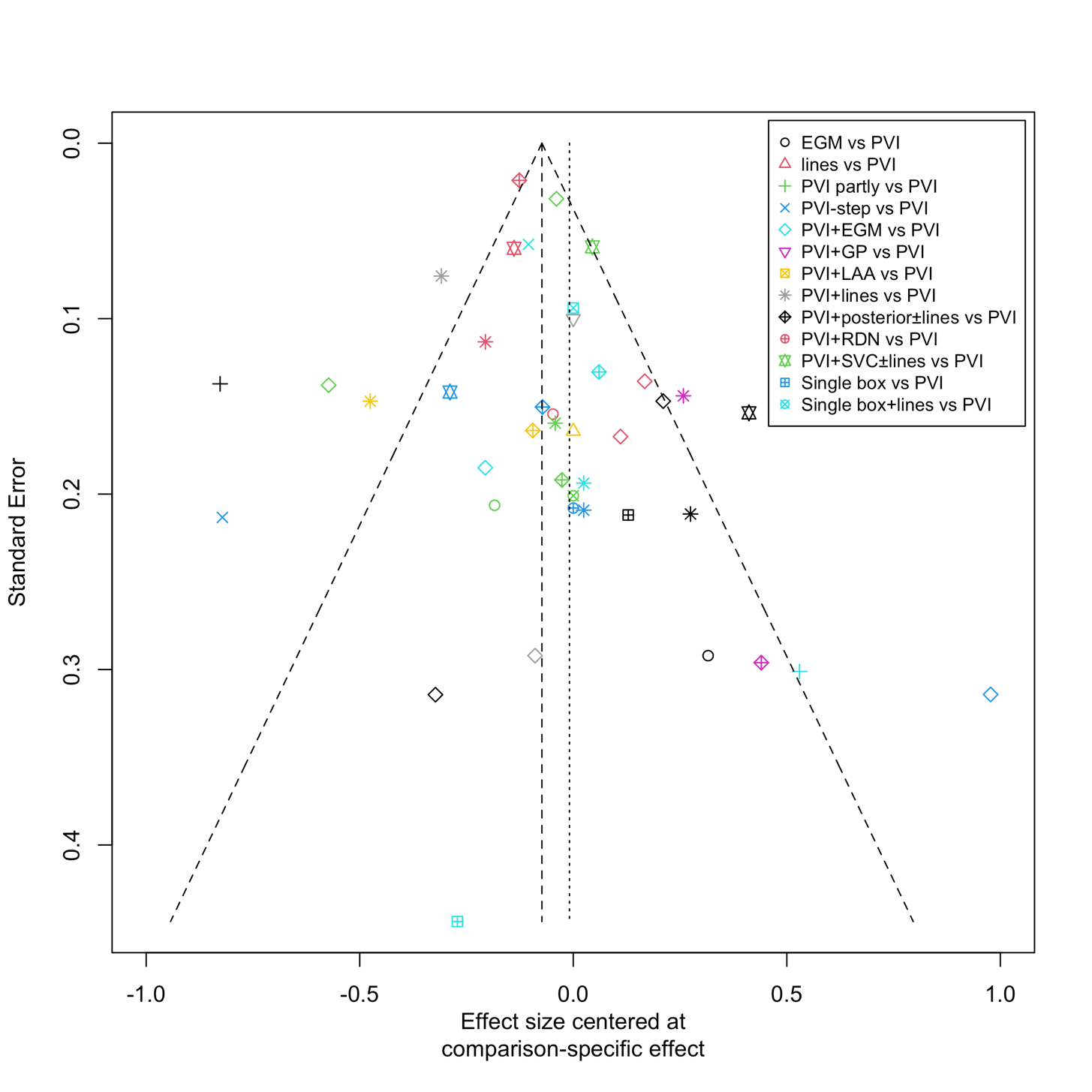
**
