# Supplementary material for: Comparing efficacy and safety in catheter ablation strategies for atrial fibrillation: a network meta-analysis
Source: BMC Med. 2022 May 31;20:193. doi: 10.1186/s12916-022-02385-2 (PMC9153169; doi:10.1186/s12916-022-02385-2)
Supplement: Supplementary file 11 — Additional file 11. Subgroup analyses. 1 Depending on AF detection device. 2 Depending on AAD or reablation allowance during the follow-up. 3 Depending on follow-up duration. 4 Depending on publication year. 5. Subgroup analysis on the type of AF included in the original studies (PAF, non-PAF, and mixed). 6 Subgroup analysis depending on the blanking period (cut-off 8 weeks). [file 12916_2022_2385_MOESM11_ESM.docx]

**Additional file 11. SUBGROUP ANALYSES**

**1 Depending on AF detection device**

| **Detection devices with recording times ≥7 (21 RCTs)** | **Detection devices with recording times <7 (46 RCTs)** |
| --- | --- |
| τ^2^= 0.066  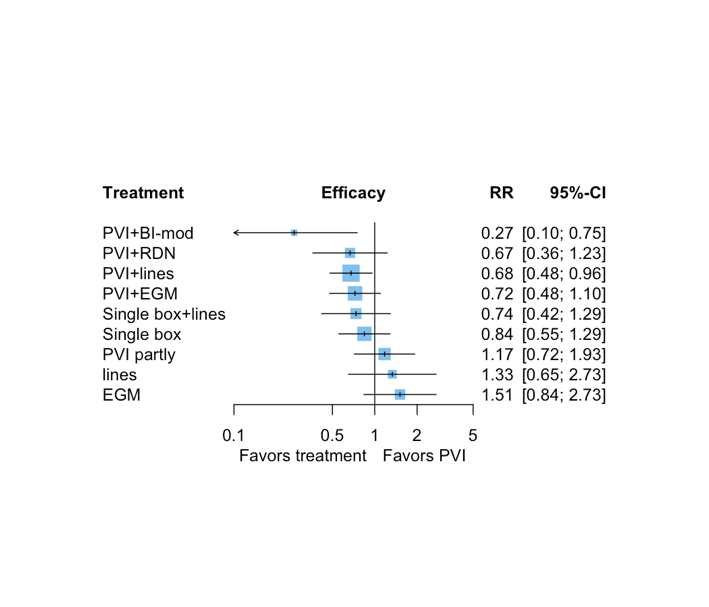Disconnected components excluded:  - GP *vs* PVI+GP  - PVI+posterior+lines *vs* PVI+SUB-mod | τ^2^=0.123  **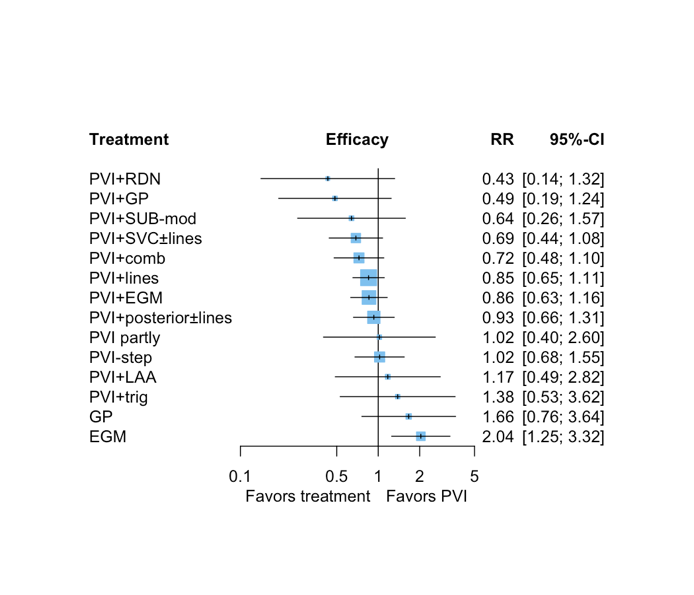** |
| τ^2^= 0.0825 | τ^2^= 0.000  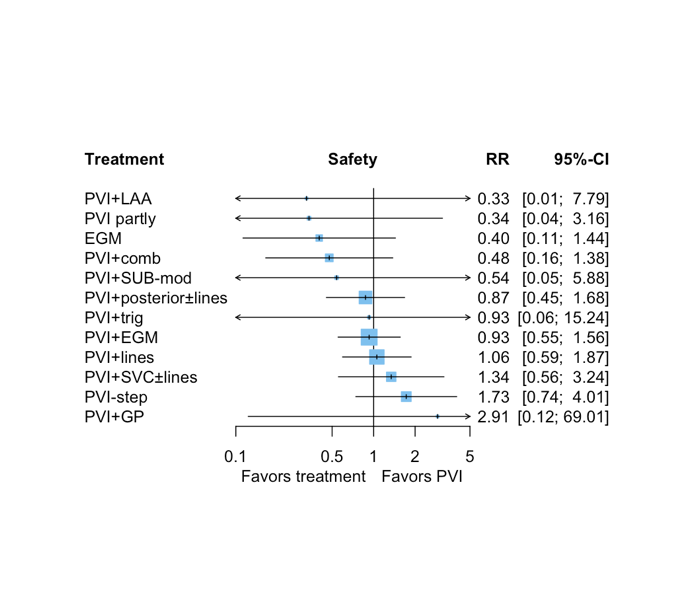 |


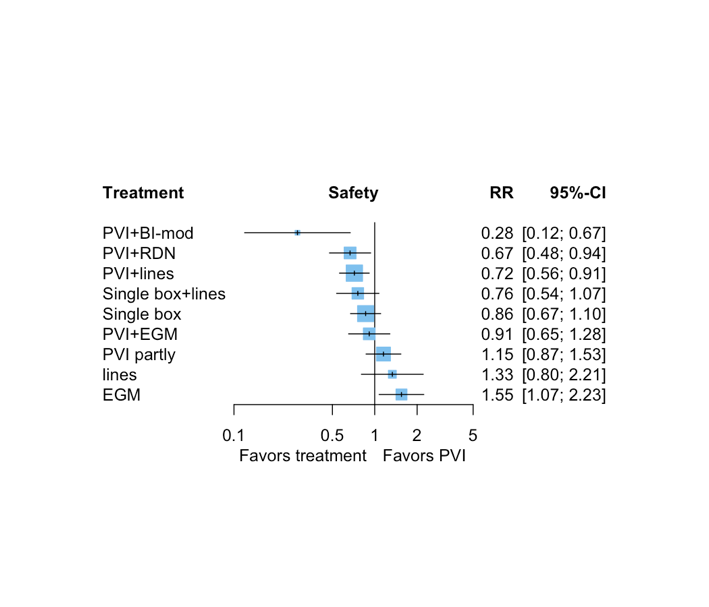


**2 Depending on AAD or reablation allowance during the follow-up**

| **Not allowing for ADD (48 RCTs)** | **Allowing for ADD (19 RCTs)** |
| --- | --- |
| τ^2^= 0.084  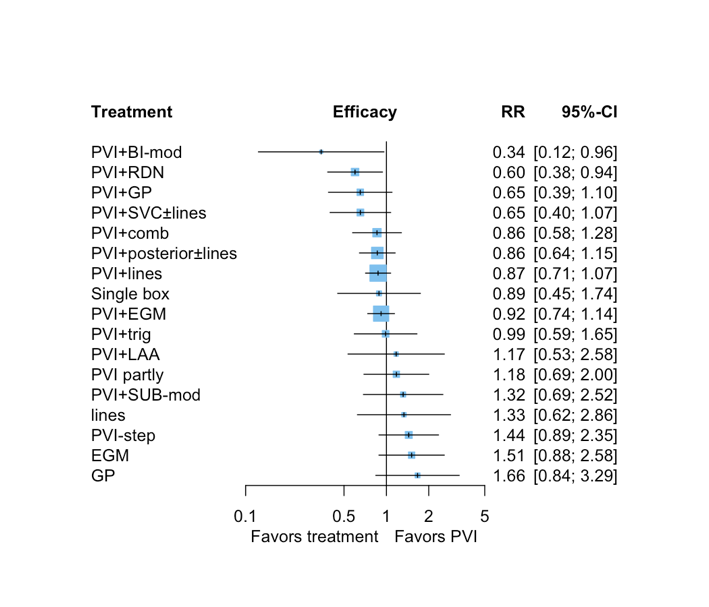 | τ^2^=0.1645  **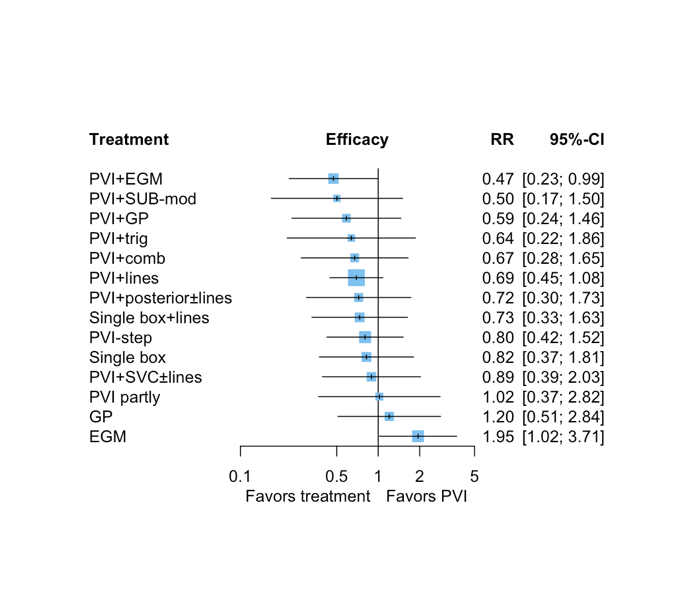** |
| τ^2^=0  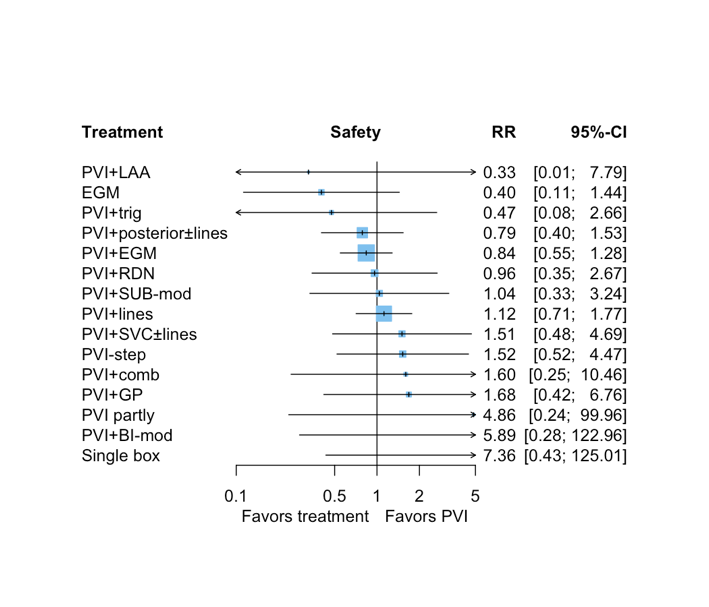 | τ^2^=0.1802  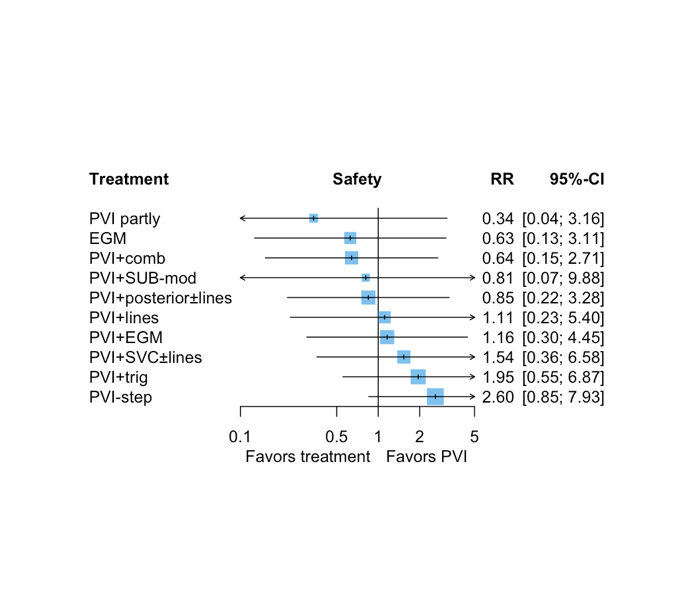 |

**3 Depending on follow-up duration**

| **Follow-up** $\boldsymbol{\geq}$ **12 months (64 studies)** | **Follow-up < 12 months (3 studies)** |
| --- | --- |
| τ^2^= 0.0915  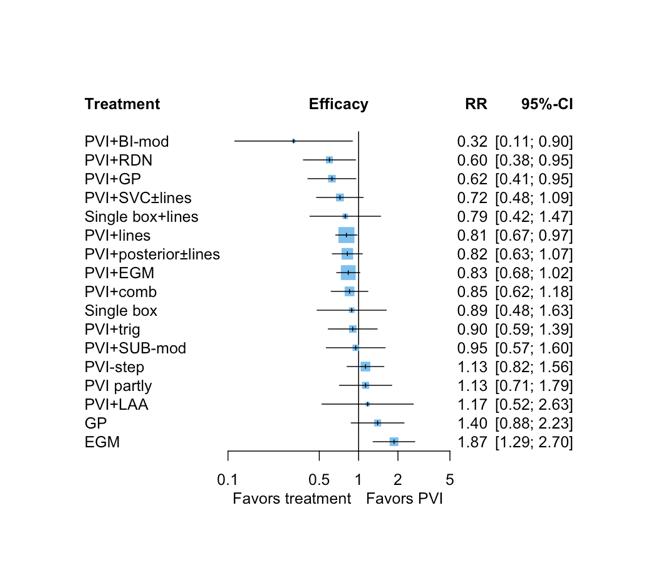 | τ^2^=0.3221  **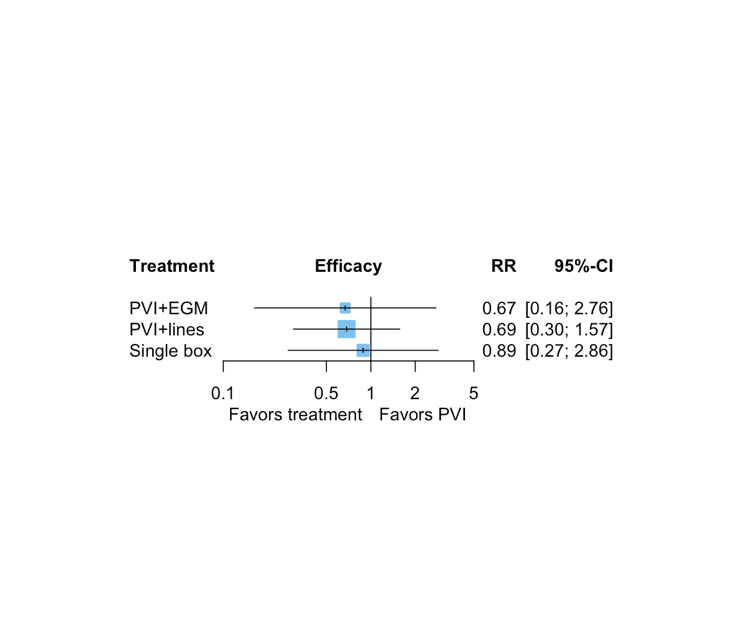** |
| τ^2^=0  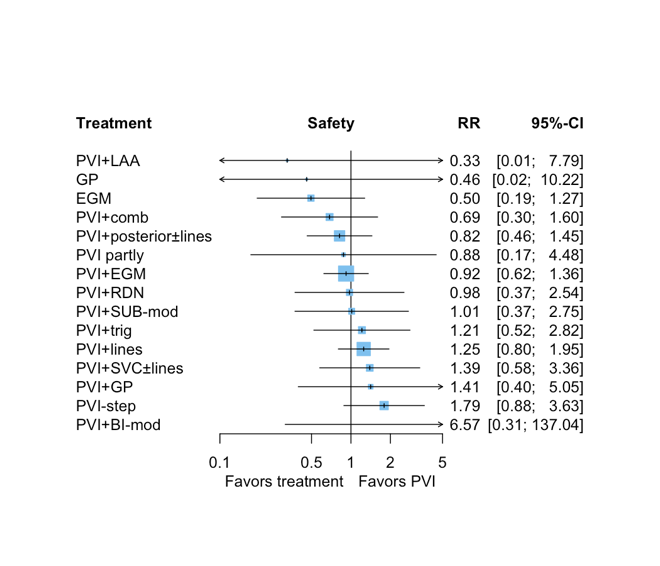 | τ^2^=0  **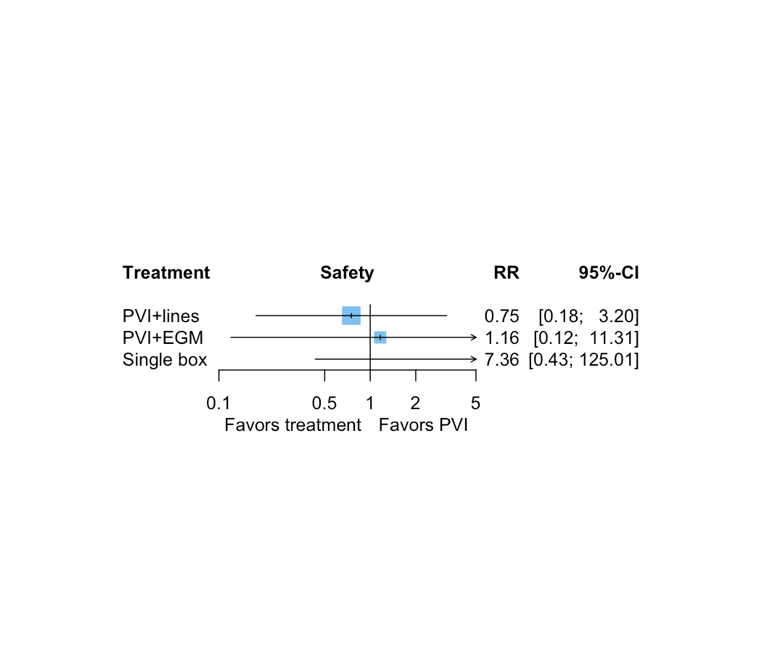** |

**4 Depending on publication year**

| **Publication year** $\boldsymbol{\geq}$ **2011 (45 studies)** | **Publication year < 2011 (22studies)** |
| --- | --- |
| τ^2^=0.0683  **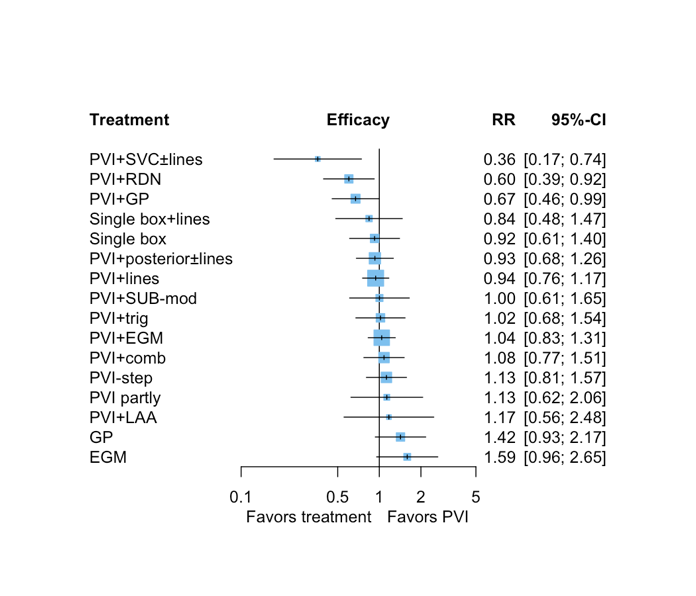** | τ^2^=0.0827  **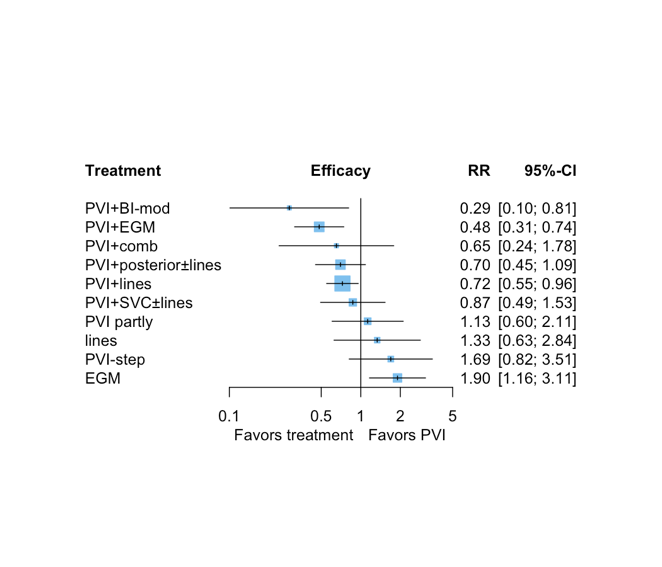** |
| τ^2^=0  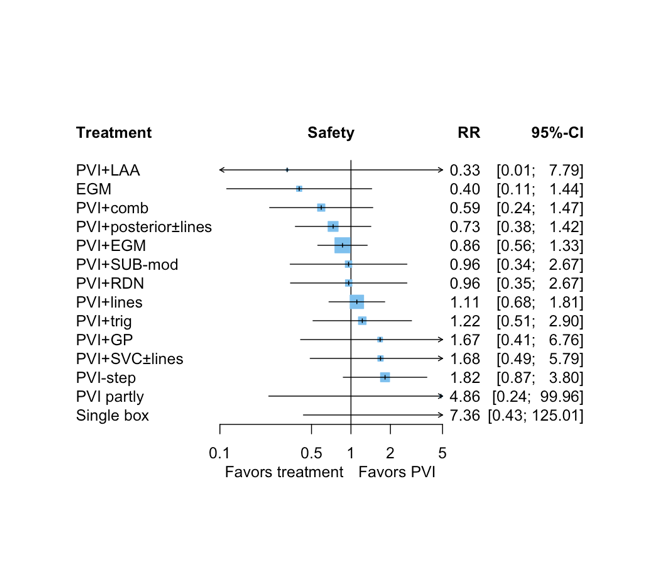 | τ^2^=0.000  **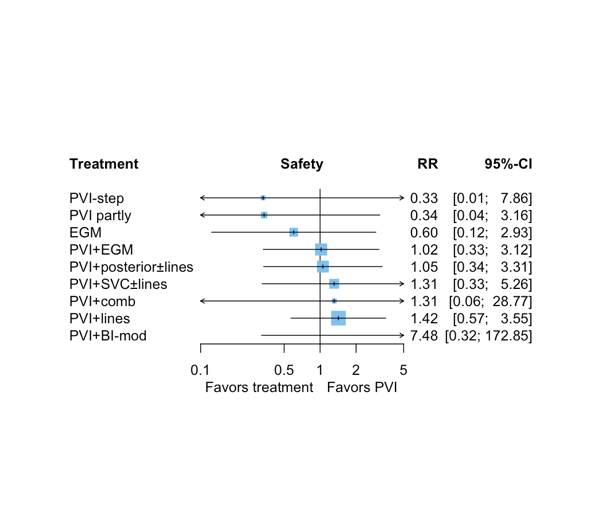** |

**PAF (25 RCTs)**

**5. Subgroup analysis on the type of AF included in the original studies (PAF, non-PAF, and mixed)**

Non PAF (24 RCTs)

| τ^2^= 0.1041  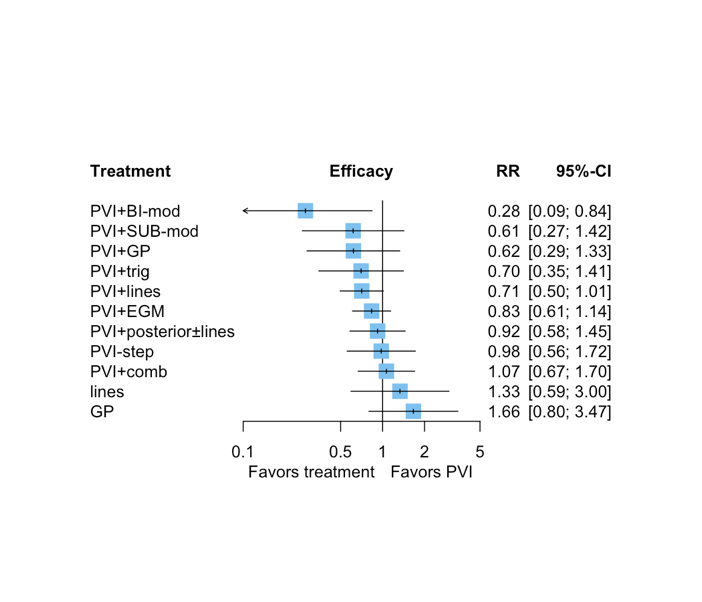 | τ^2^= 0  **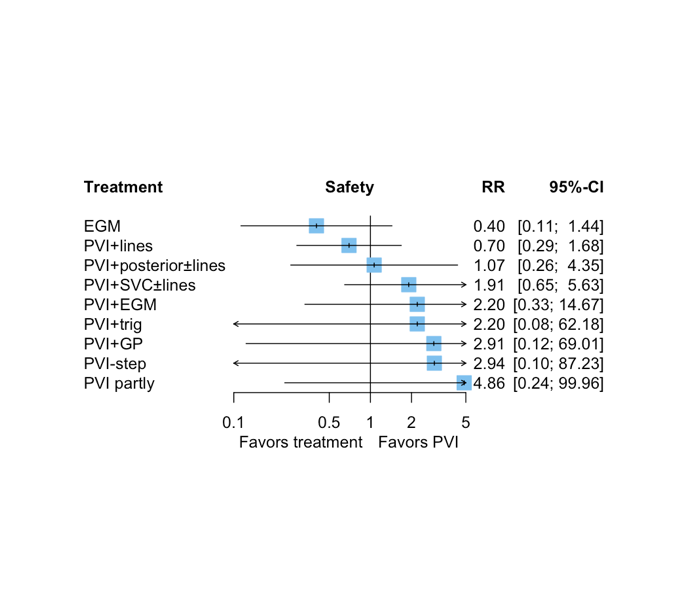** |
| --- | --- |

| **τ^2^= 0.2015**  **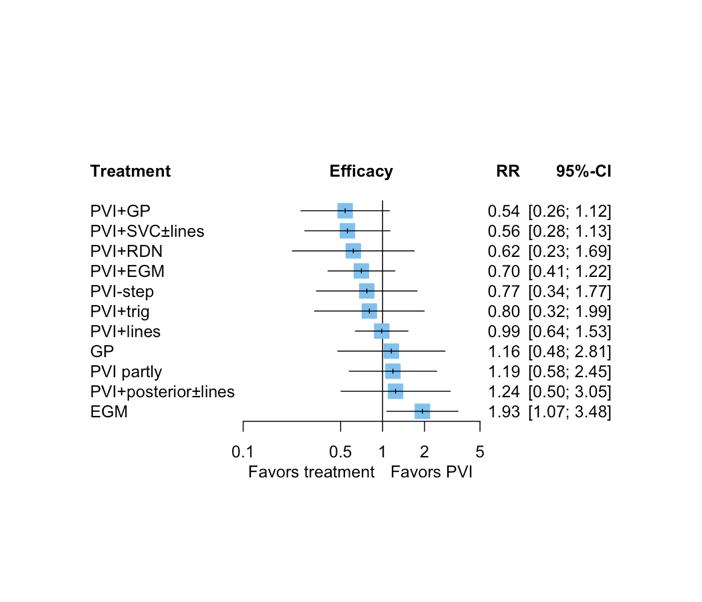** | **τ^2^= 0**  **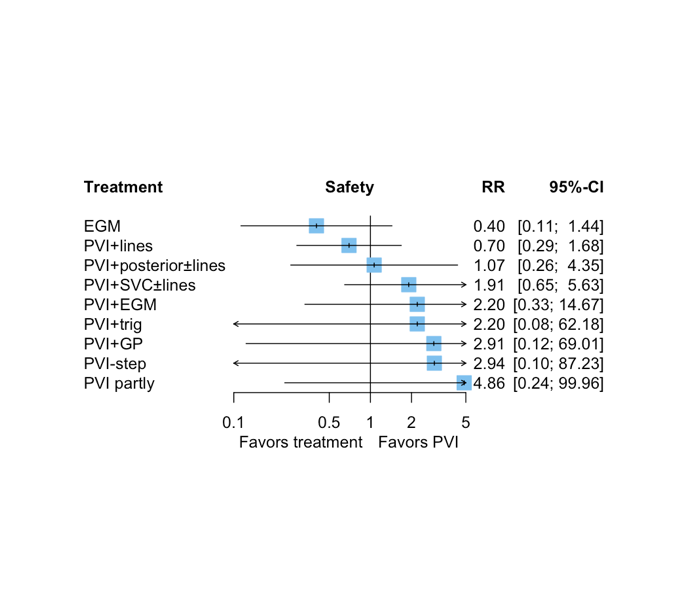** |
| --- | --- |

**MIXED (18 RCTs)**

| τ^2^=0.01132  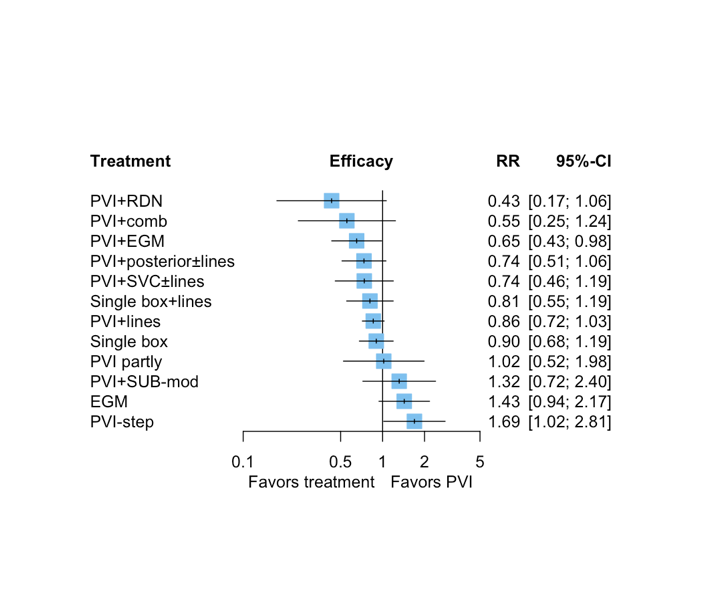 | τ^2^= 0  **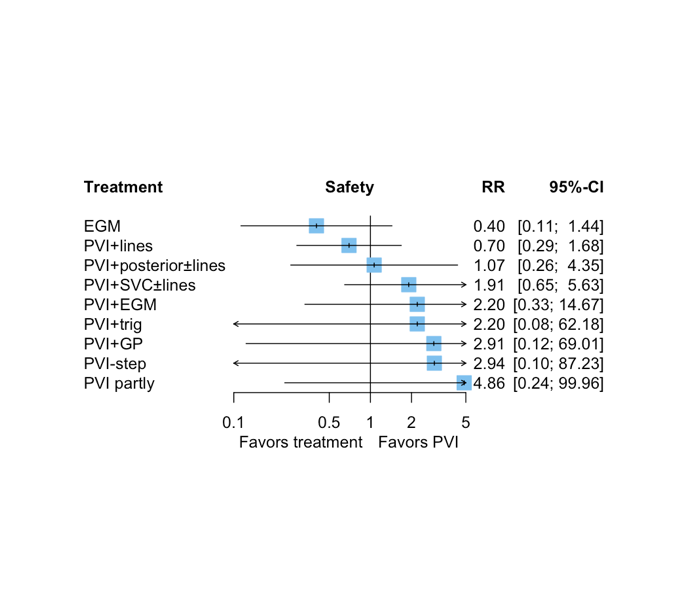** |
| --- | --- |

**6 Subgroup analysis depending on the blanking period (cut-off** $\boldsymbol{\geq}$ **8 weeks)**

**Blanking < 8 weeks (13 RCTs) Blanking** $\boldsymbol{\geq}$**8 weeks (53 RCTs)**

| τ^2^= 0  **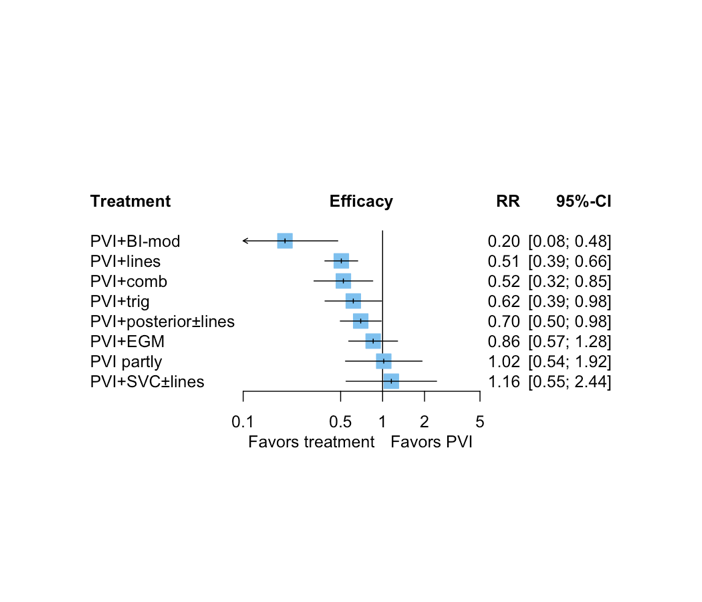** | τ^2^= 0.066  **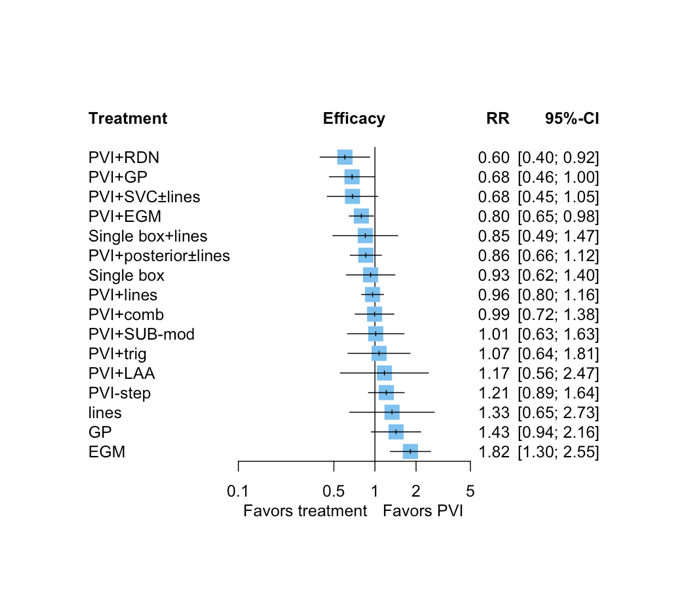** |
| --- | --- |
| τ^2^= 0  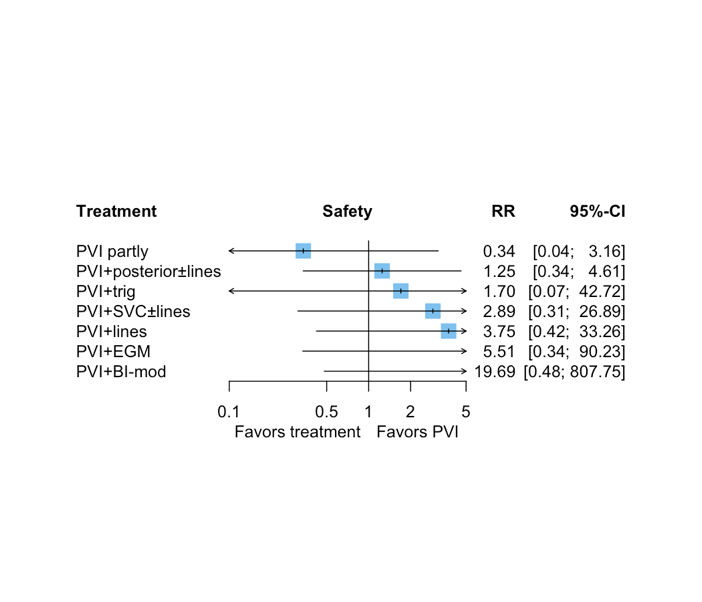 | τ^2^= 0  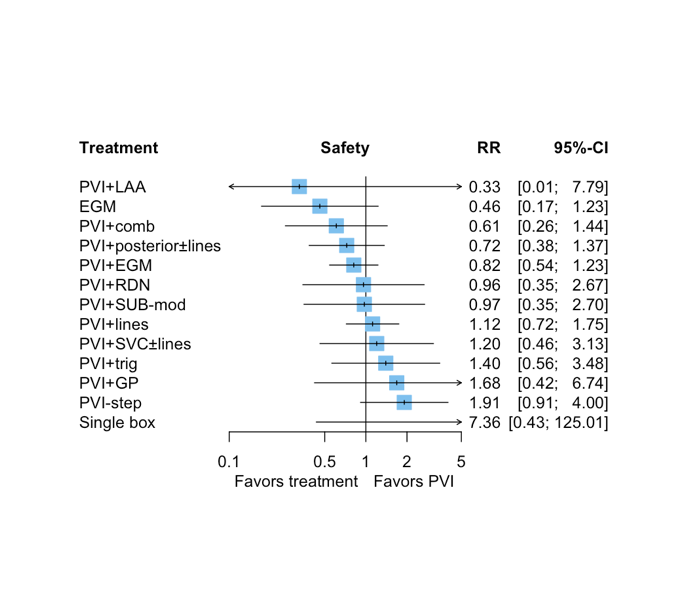 |
