# Supplementary material for: Comparing efficacy and safety in catheter ablation strategies for atrial fibrillation: a network meta-analysis
Source: BMC Med. 2022 May 31;20:193. doi: 10.1186/s12916-022-02385-2 (PMC9153169; doi:10.1186/s12916-022-02385-2)
Supplement: Supplementary file 12 — Additional file 12. Meta-regression. Table S1- [Meta-regression coefficients, alongside Credible Intervals and percentage reduction in heterogeneity for efficacy outcome]. [file 12916_2022_2385_MOESM12_ESM.docx]

**Additional file 12. META-REGRESSION**

We fitted network meta-regression models to investigate the impact of some prespecified variables that may act as effect modifiers on the primary outcome efficacy.

We used a random-effects meta-regression model with common consistent coefficients. Statistical details for the specific model used are described in the following.

For each study $i=1, \ldots, N$ let $r_{ik}$ be the number of events in arm $k=1, \ldots, K$and $n_{ik}$ the sample size in arm $k$. As we have binary outcome data, we have:

$r_{ik}\sim Binom\left( \pi_{i,k},n_{ik} \right)$ (Binomial likelihood),

where the probability $\pi_{i,k}$ is parametrized as

$$\mathrm{logit}\left( \pi_{i,k} \right)=\mu_{i} if k=1$$

$$\mathrm{logit}\left( \pi_{i,k} \right)=\mu_{i}+\theta_{i,k} if k\geq2$$

$$\theta_{i,k}=\delta_{i,1k}+\beta_{t_{i1},t_{ik}}x_{i}$$

where $\beta_{t_{i1},t_{ik}}=\beta_{1,t_{ik}}-\beta_{1,t_{i1}}$ is the difference in the relative treatment effect of $t_{i,k} vs t_{i,1}$ per increase of one unit in the covariate $x_{i}$

In a random‐effects model, $\delta_{i,1k}$ (with *k* ≥ 2) represents the trial‐specific relative treatment effect when the covariate is 0 and is assumed to follow a normal distribution

$$\delta_{i,1k}\sim N\left( d_{t_{i1},t_{i,k}},\tau^{2} \right)$$

with $d_{t_{i1},t_{i,k}}=d_{1,t_{i,k}}-d_{1,t_{i,1}}$ being the mean relative treatment effect of $t_{i,k} vs t_{i,1}$ when the covariate is 0.

Then, as we use a common regression coefficient model, we assume

$$\beta_{t_{i1},t_{ik}}=\beta$$

For each covariate analysed, the model is estimated within a Bayesian framework (R library gemtc version 0.8.7) using the following priors:

$$\mu_{i}\sim N\left( 0,1000 \right),d_{1,t_{i,k}}\sim N\left( 0,1000 \right),\beta\sim N\left( 0,1000 \right)$$

$$\tau\sim U\left( 0,5 \right)$$

We included as covariates in separate network meta-regression models the following variables: age, percentage of males, publication year, presence of hypertension, coronary artery disease (CAD), structural heart disease (SHD), left atrial dimensions, duration of follow-up, and usage of AF detection device. The impact of each covariate was assessed in independent univariate analyses. Continuous covariates were cantered at the mean (subtracting the mean covariate value from each covariate). Not all the studies reported all the covariates of interest, so if a study was missing a covariate, it was omitted from that regression model. Only effect modifiers which non-missing outcomes for at least 10 studies were retained. Results are reported in eTable 7. For each meta-regression analysis, regression coefficients are reported alongside their CI: all CIs contain zero, meaning that no significative coefficient was present in the analysis. This suggests that none of the effect modifiers analyzed seem to impact our NMA results. For some covariates, the estimation of heterogeneity varied (considerably for some variables and moderately for others) with respect to the main analysis, indicating that accounting for possible effect modifiers could partially explain the heterogeneity.

**Table S1.** Meta-regression coefficients, alongside Credible Intervals and percentage reduction in heterogeneity for efficacy outcome.

| **Covariate** | **N studies** | | |  |  | | | **Coefficient** | | **95% CrI** | **%**$\boldsymbol{\tau}^{\boldsymbol{2}}$ **reduction** |
| --- | --- | --- | --- | --- | --- | --- | --- | --- | --- | --- | --- |
| Age | 77 |  |  | | | 0.2510 | | | (-0.3106, 0.81220) | | **44.48**% |
| % males | 46 | | |  |  | | 0.3297 | | (-0.2245, 0.86381) | | **22.1**% |
| Publication year | 65 | | |  |  | | 0.5792 | | (-0.0540, 1.21221) | | 0% |
| Hypertension | 40 | | |  |  | | **-1.0071** | | **(-1.9707, -0.02440)** | | 6.52% |
| CAD | 23 | | |  |  | | -0.3986 | | (-1.1735, 0.40820) | | **80.86%** |
| SHD | 66 | | |  |  | | -0.4637 | | (-0.9516, 0.02871) | | 6.95% |
| Left atrial dimension | 66 | | |  |  | | -0.1510 | | (-0.6531, 0.36752) | | 0% |
| Duration of follow-up | 66 | | |  |  | | 0.2017 | | (-0.2788, 0.693354) | | 0.70% |
| AF detection device |  | | |  |  | | -0.2091 | | (-0.7751, 0.34178) | | 0% |
