# Supplementary material for: Comparing efficacy and safety in catheter ablation strategies for atrial fibrillation: a network meta-analysis
Source: BMC Med. 2022 May 31;20:193. doi: 10.1186/s12916-022-02385-2 (PMC9153169; doi:10.1186/s12916-022-02385-2)

Additional file 13. SENSITIVITY ANALYSES

1 Excluding high risk of bias RCTs (57 RCTs left)

τ^2=0.086

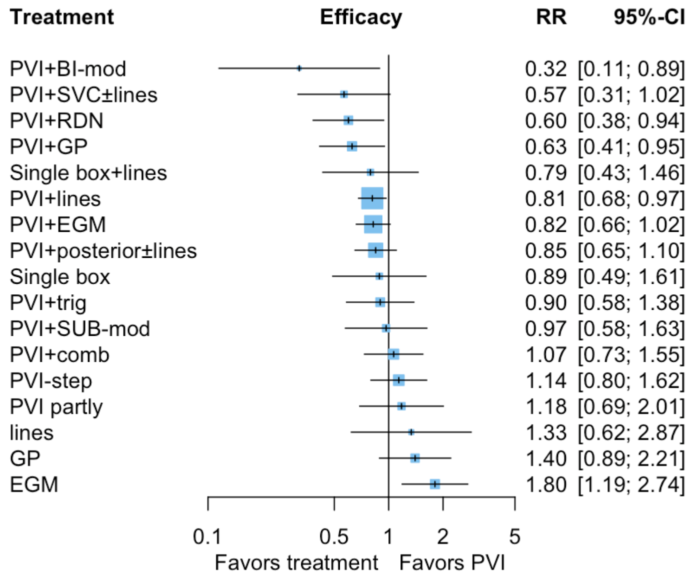

τ^2=0

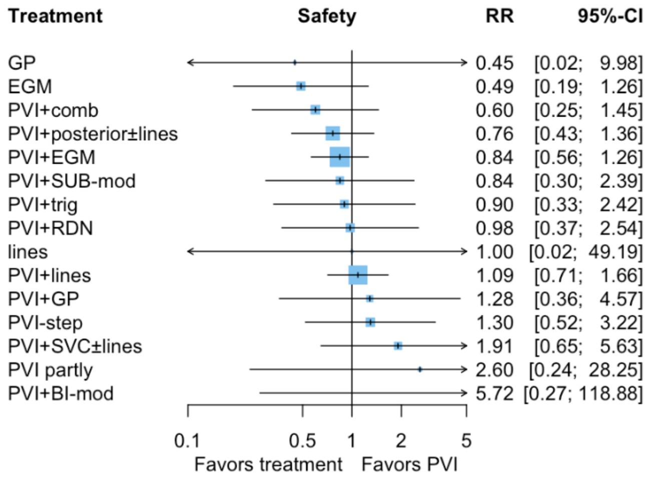

2 Excluding RCTs with Renal Denervation (RDN) treatment (64 RCTs left)

τ^2=0.087

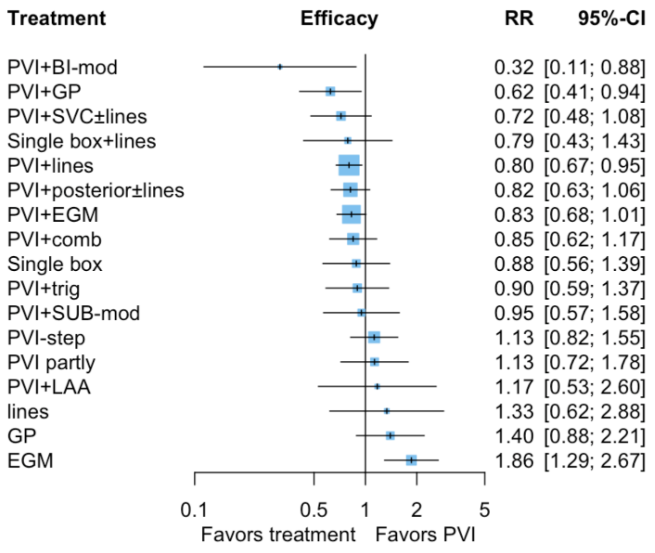

$\tau^2 = 0.048$

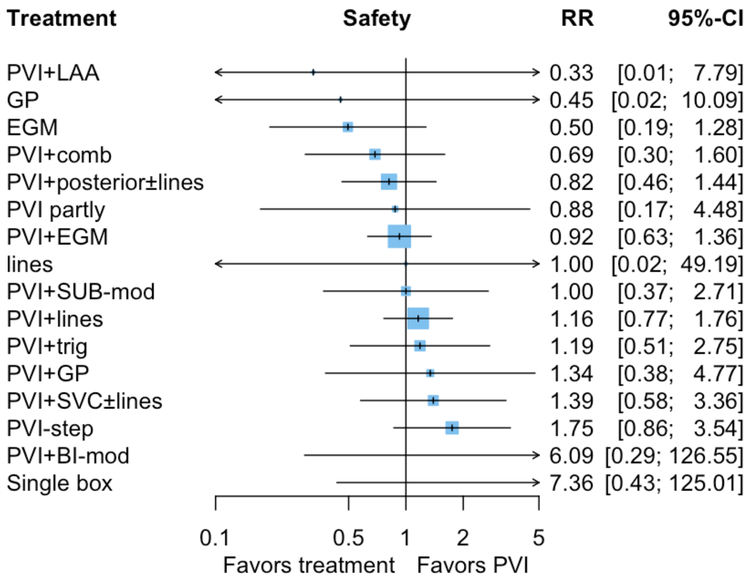

Treatment
Safety
RR
95%-CI
PVI+LAA 0.33 [0.01; 7.79]
GP 0.45 [0.02; 10.09]
EGM 0.50 [0.19; 1.28]
PVI+comb 0.69 [0.30; 1.60]
PVI+posterior±lines 0.82 [0.46; 1.44]
PVI partly 0.88 [0.17; 4.48]
PVI+EGM 0.92 [0.63; 1.36]
lines 1.00 [0.02; 49.19]
PVI+SUB-mod 1.00 [0.37; 2.71]
PVI+lines 1.16 [0.77; 1.76]
PVI+trig 1.19 [0.51; 2.75]
PVI+GP 1.34 [0.38; 4.77]
PVI+SVC±lines 1.39 [0.58; 3.36]
PVI-step 1.75 [0.86; 3.54]
PVI+BI-mod 6.09 [0.29; 126.55]
Single box 7.36 [0.43; 125.01]
0.1
0.5
1
2
5
Favors treatment
Favors PVI

3 Excluding RCTs with only PAF patients (42 RCTs left)

τ^2=0.080

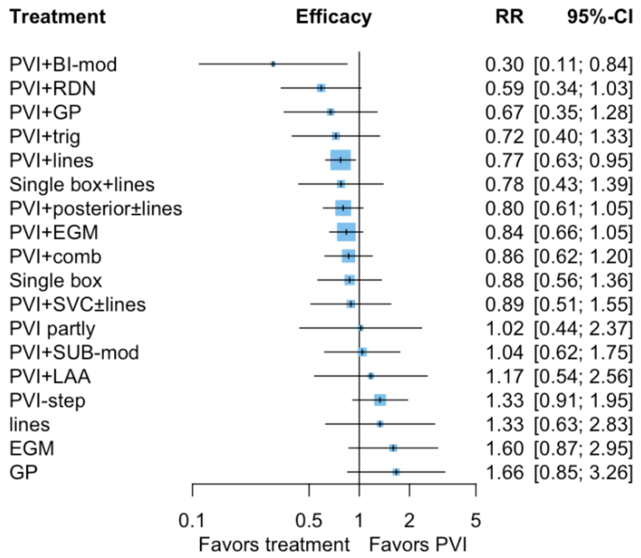

τ^2=0

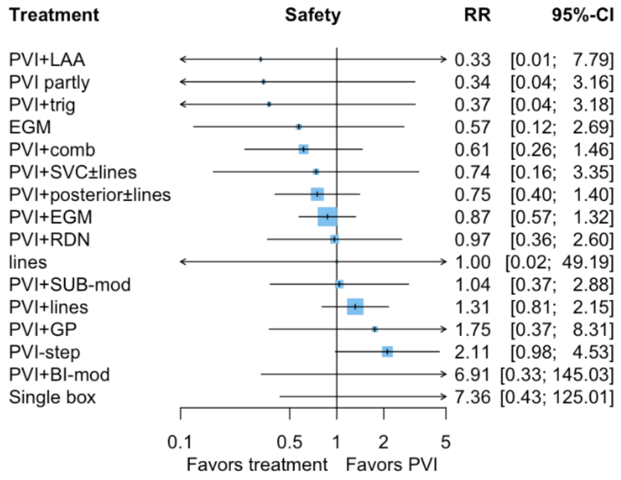

4 Excluding catheter 8mm, 8mm plus 3.5mm irrigated, 8mm and 4mm irrigated (55 RCTs left)

τ^2=0.09

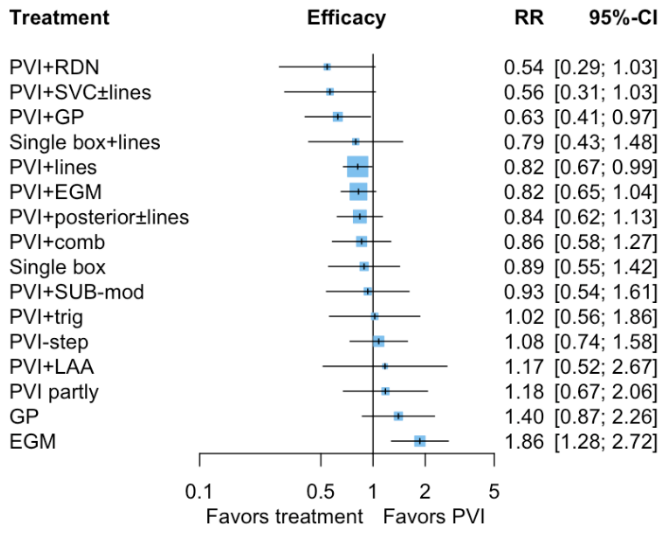

τ^2=0

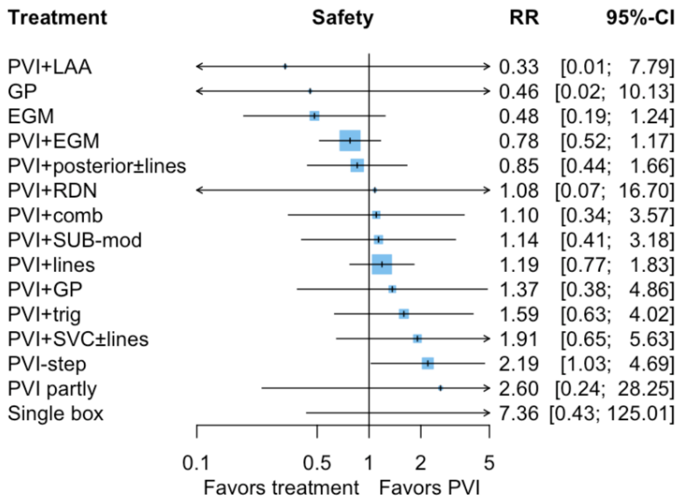

5 INCLUDING RCTs with antiarrhythmic drugs (AADs) as control arm (78 total RCTs)

Table S1 Risk of Bias assessment with domains (additional 11 RCTs of the AADs sensitivity analysis).

Study	D1	D2	D3	D4	D5	Overall
	Risk of bias domains					
Di Biase et al. a	+	-	+	+	+	-
Cosedis Nielsen et al.	+	X	+	+	+	X
Jones et al.	-	X	-	+	+	X
Krittayaphong et al.	-	X	+	+	+	X
Oral et al. a	-	-	+	+	+	-
Packer et al.	-	X	-	+	+	X
Pappone et al. c	+	-	+	+	+	-
Prabhu et al.	+	X	-	+	+	X
Sohara et al.	-	X	+	+	+	X
Stabile et al.	+	X	+	+	+	X
Wazni et al.	+	X	+	+	+	X

Domains:
D1: Bias arising from the randomization process.
D2: Bias due to deviations from intended intervention.
D3: Bias due to missing outcome data.
D4: Bias in measurement of the outcome.
D5: Bias in selection of the reported result.

Judgement
X High
- Some concerns
+ Low

Table S2. Characteristics of the additional 11 RCTs included in sensitivity network meta-analysis, including also RCTs with AADs as comparison arm.

Study	Year of publication	blanking period (weeks)	follow up period (months)	Total number (n)	Strategies	Number of patients available for analysis of efficacy	Age (mean ± SD)	Sex (% male)	PAF (%)	Hypertension (%)	CAD (%)	CHF (%)	SHD (%)	LVEF % (mean±SD)	LAD mm(mean±SD)
Cosedis Nielsen et al.[89]	2012	12	24	294	PVI+lines	146	56±9	100 (68)	146 (100)	43 (29)	6 (4)	15 (10)	NR	NR	40±6
					AADs	148	54±10	106 (72)	148 (100)	53 (36)	2 (1)	20 (14)	NR	NR	40±5
Di Biase et al. [90]	2016	12	24	203	PVI+combination (lines and egm)	102	62±10	77 (75)	0	46 (45)	63 (62)	102(100)	102(100)	29±5	47±4
					AADs	101	60±11	74 (73)	0	48 (48)	66 (65)	101(100)	101(100)	30±8	48±5
Jones et al. [91]	2013	8	12	62	PVI+lines	25	64±10	21 (81)	0	NR	25 (100)	25 (100)	25 (100)	22±8	50±6
					AADs	26	62±9	24 (92)	0	NR	25 (100)	25 (100)	25 (100)	25±7	46±7
Krittayaphong et al. [92]	2003	NR	12	30	PVI+lines	14	55±10	11 (73)	10 (60)	4 (27)	1 (6.7%)	NR	NR	64±10	40±8
					AADs	15	49±15	8 (53)	11 (73)	7 (47)	1 (6.7%)	NR	NR	62±9	39±7
Oral et al. [93]	2006	NR	12	146	PVI+lines	77	55±9	67 (87)	0	NR	3 (4)	NR	6 (8)	55±7	45±6
					AADs	69	58±8	62 (90)	0	NR	3 (6)	NR	6 (9)	56±7	45±5
Packer et al. [94]	2013	12	12	163	PVI	163	57±9	125 (77)	127 (78)	67 (41)	13 (11)	11 (7)	NR	60±6	40±5
					AADs	82	56±9	64 (78)	64 (78)	45 (37)	8 (10)	5 (6)	NR	61±6	41±6
Pappone et al. [95]	2006	6	12	198	PVI+lines	99	55 ± 10	69 (70)	99 (100)	55 (56)	2 (2)	NR	NR	60±8	40±6
					AADs	99	57 ± 10	64 (65)	99 (100)	56 (57)	2 (2)	NR	NR	61±6	38±6
Prabhu et al. [96]	2017	4	6	68	PVI+posterior box±lines	33	59±11	31 (94)	0	13 (39)	NR	33 (100)	33 (100)	35±10	48±6
					AADs	33	62±9	29 (88)	0	12 (36)	NR	33 (100)	33 (100)	35±9	47±8

Sohara et al. [97]	2016	12	9	153	PVI	100	59±10	80 (80)	100 (100)	51 (51)	3 (3)	NR	NR	67±6	38±6
					AADs	43	61±10	35 (81)	43 (100)	24 (56)	2 (5)	NR	NR	67±7	38±5
Stabile et al. [98]	2006	4	12	137	PVI+lines	68	62.2 ±9	37 (54)	42 (61)	36 (52.9)	3 (4.4)	NR	43 (63)	59±7	46±5
					AADs	69	62.3 ±11	44 (64)	50 (73)	34 (49.3)	3 (4.4)	NR	43 (62)	58±6	45±6
Wazni et al. [99]	2005	NR	12	70	PVI	32	53±8	NR	32 (97)	NR	NR	NR	NR	53±5	41±8
					AADs	35	54±8	NR	35 (95)	NR	NR	NR	NR	54±6	42±7

Network forest plots for efficacy and safety vs PVI (including AADs as control arm)

τ^2=0.087

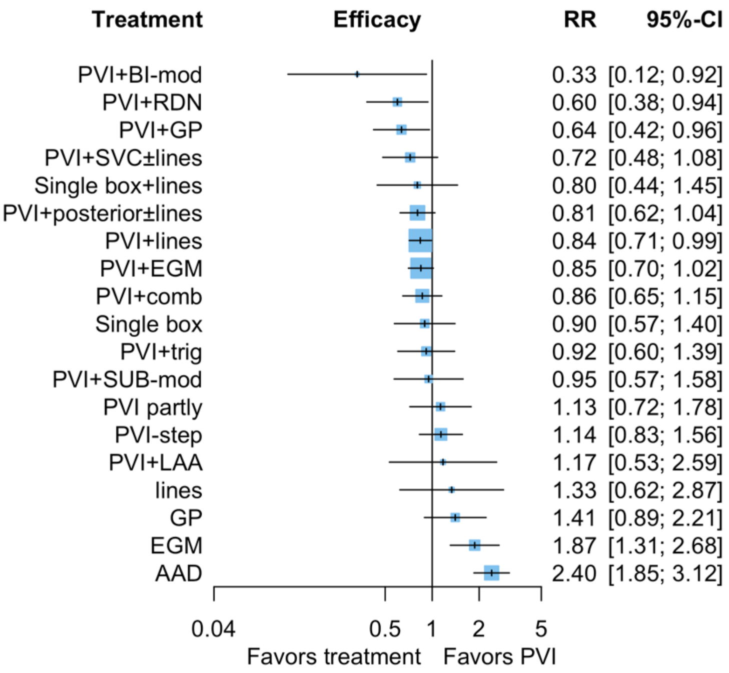

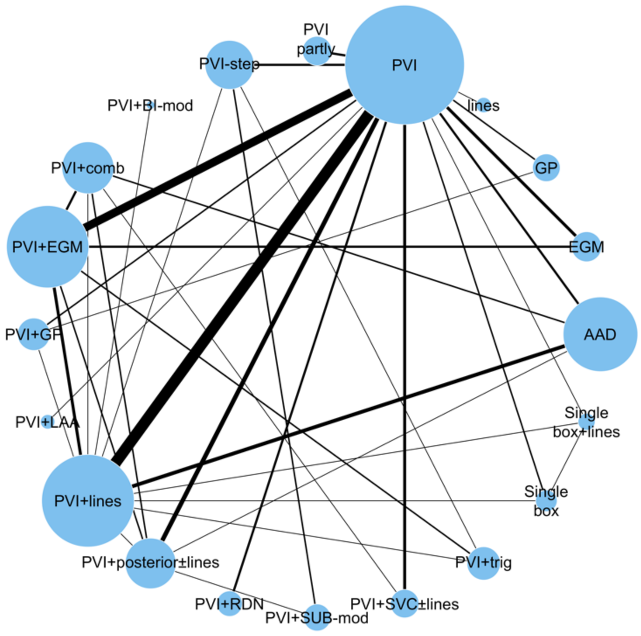

$\tau^2=0$

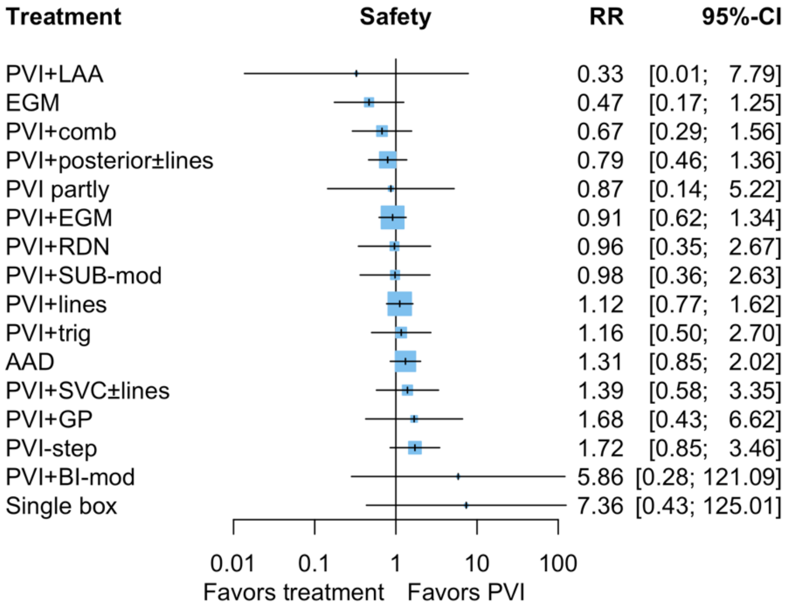
Treatment
Safety
RR
95%-CI
PVI+LAA 0.33 [0.01; 7.79]
EGM 0.47 [0.17; 1.25]
PVI+comb 0.67 [0.29; 1.56]
PVI+posterior±lines 0.79 [0.46; 1.36]
PVI partly 0.87 [0.14; 5.22]
PVI+EGM 0.91 [0.62; 1.34]
PVI+RDN 0.96 [0.35; 2.67]
PVI+SUB-mod 0.98 [0.36; 2.63]
PVI+lines 1.12 [0.77; 1.62]
PVI+trig 1.16 [0.50; 2.70]
AAD 1.31 [0.85; 2.02]
PVI+SVC±lines 1.39 [0.58; 3.35]
PVI+GP 1.68 [0.43; 6.62]
PVI-step 1.72 [0.85; 3.46]
PVI+BI-mod 5.86 [0.28; 121.09]
Single box 7.36 [0.43; 125.01]
0.01
0.1
1
10
100
Favors treatment
Favors PVI

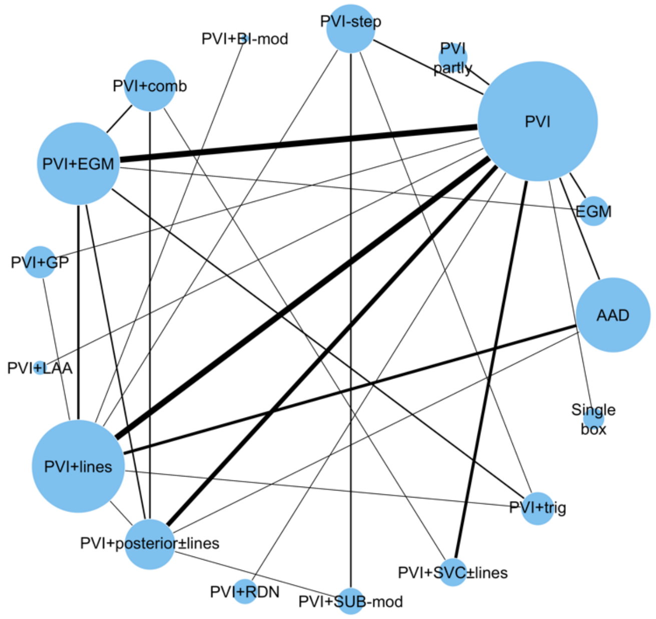
PVI-step
PVI+BI-mod
PVI partly
PVI+comb
PVI
PVI+EGM
EGM
PVI+GP
AAD
PVI+LAA
Single box
PVI+lines
PVI+trig
PVI+posterior±lines
PVI+SVC±lines
PVI+RDN
PVI+SUB-mod

Network Forest plots for efficacy and safety vs AAD

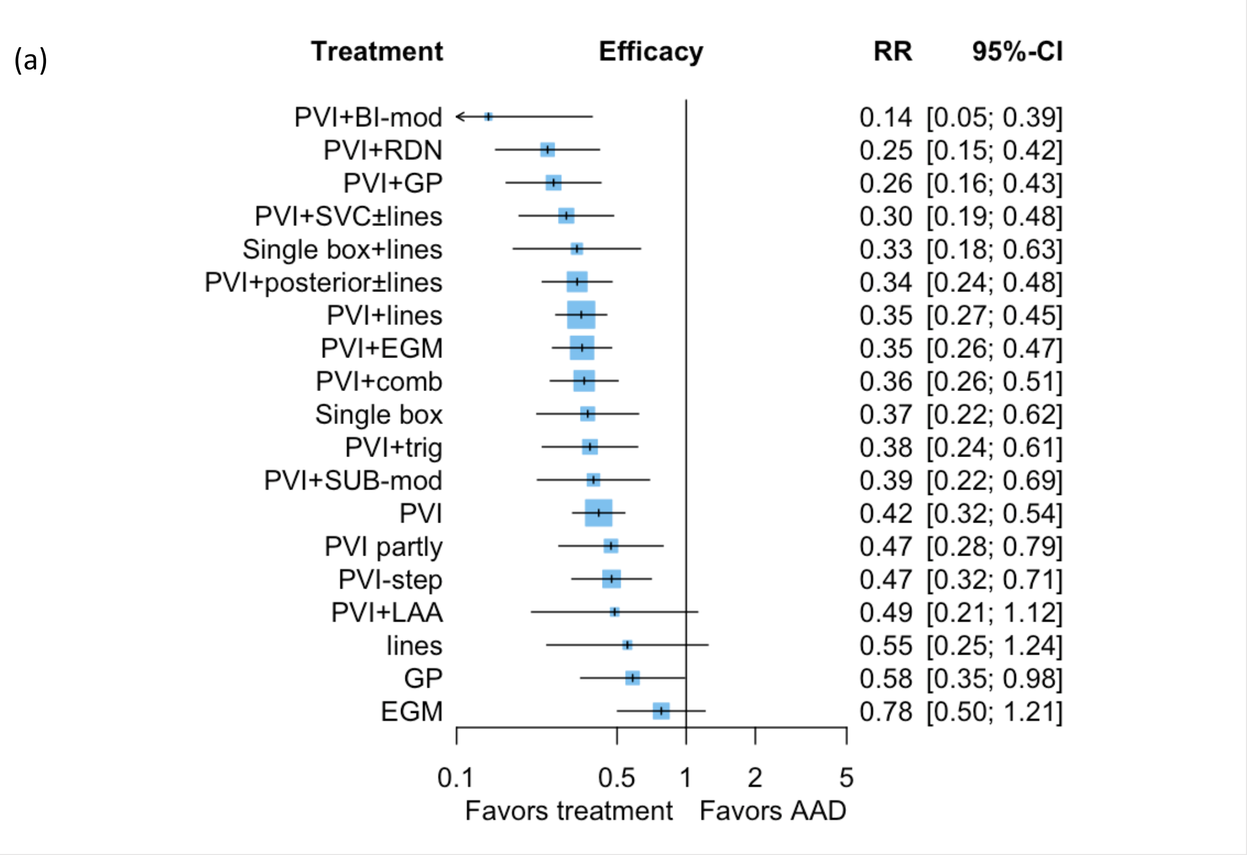

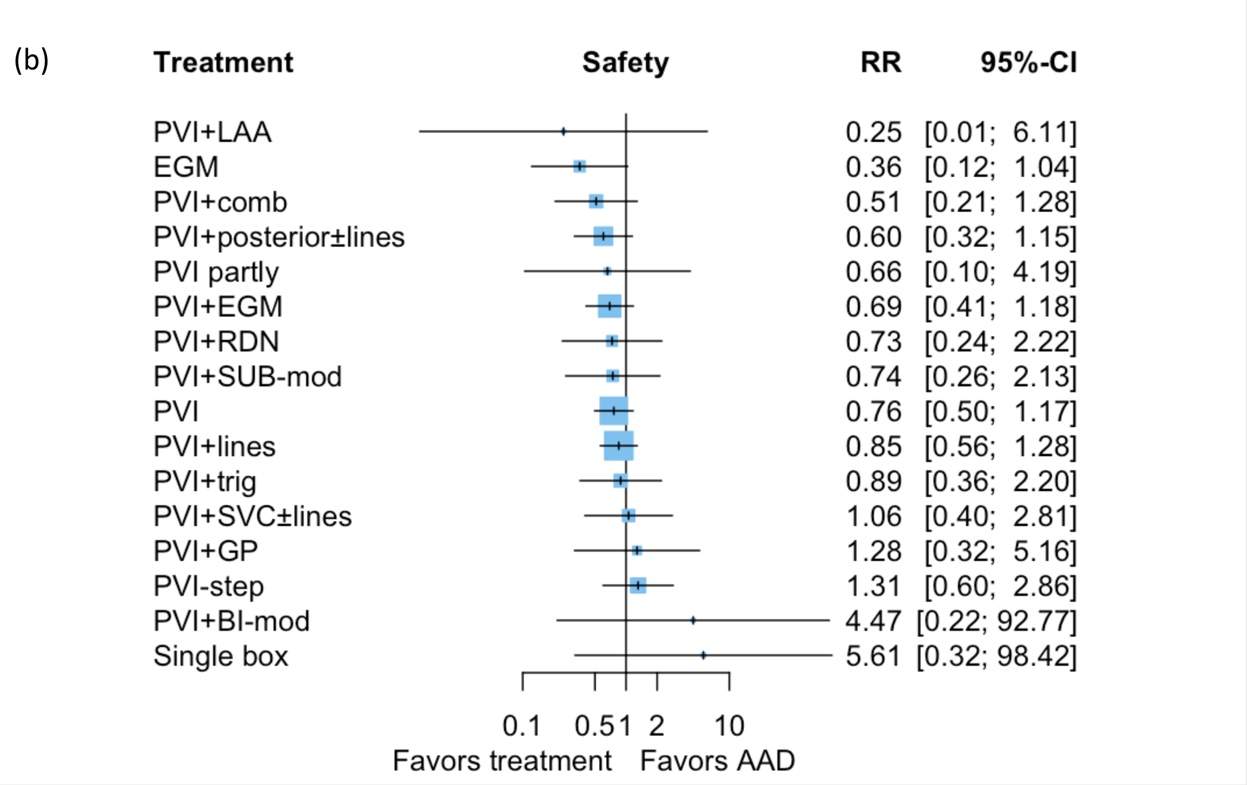

6. Sensitivity analysis with reduced categories

Interventions included in the NMA	Abbreviations of Interventions included in NMA	New categories
Electrocardiogram based ablation	EGM	Non-PVI
Ganglia Plexi ablation	GP	Non-PVI
Non-PVI lines ablation	lines	Non-PVI
Pulmonary vein isolation	PVI	PVI
PVI and Bi-atrial modification	PVI + BI-mod	PVI and additional lines/substrate modification
PVI and combination of additional lines ablation and electrocardiogram-based ablation	PVI + comb	PVI and combination of line and egm approach
PVI and electrocardiogram-based ablation	PVI + EGM	PVI and egm approach
PVI and ganglia plexi ablation	PVI + GP	PVI and autonomic modulation

**PVI and left atrial auricle isolation**	**PVI + LAA**	**PVI and additional lines/substrate modification**
**PVI and additional lines ablation**	**PVI + lines**	**PVI and additional lines/substrate modification**
**PVI, posterior box isolation ± additional lines ablation**	**PVI + posterior ± lines**	**PVI and additional lines/substrate modification**
**PVI and Renal denervation**	**PVI + RDN**	**PVI and autonomic modulation**
**PVI and substrate modification**	**PVI + SUB-mod**	**PVI and additional lines/substrate modification**
**PVI, superior vena cava isolation ± additional line ablation**	**PVI + SVC ± lines**	**PVI and additional lines/substrate modification**
**PVI and stepwise approach**	**PVI + step**	**PVI and combination of line and egm approach**
**Isolation of some pulmonary veins**	**PVI partly**	**Non-PVI**
**PVI and trigger ablation**	**PVI + trig**	**PVI and egm approach**
**Single box isolation**	**Single box**	**PVI**
**Single box isolation and additional lines**	**Single box + lines**	**PVI and additional lines/substrate modification**

N= 60 RCTs (7RCTs excluded due to identical treatments)

Efficacy

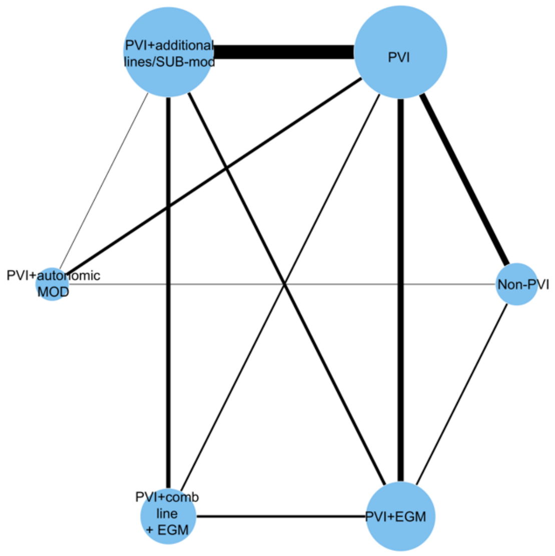

Heterogeneity **τ^2= 0.07469706**

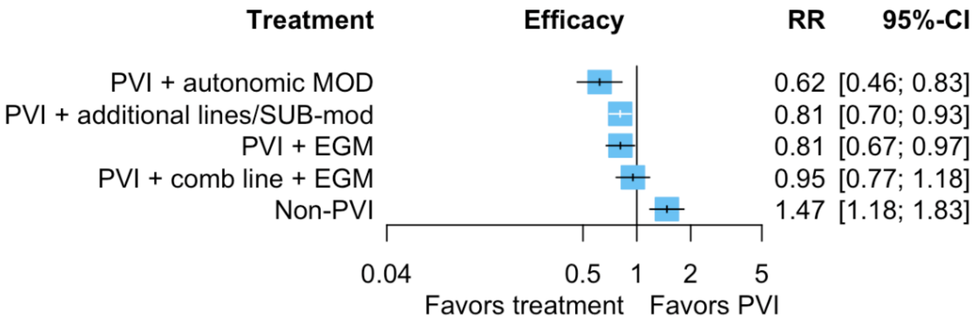

Safety:

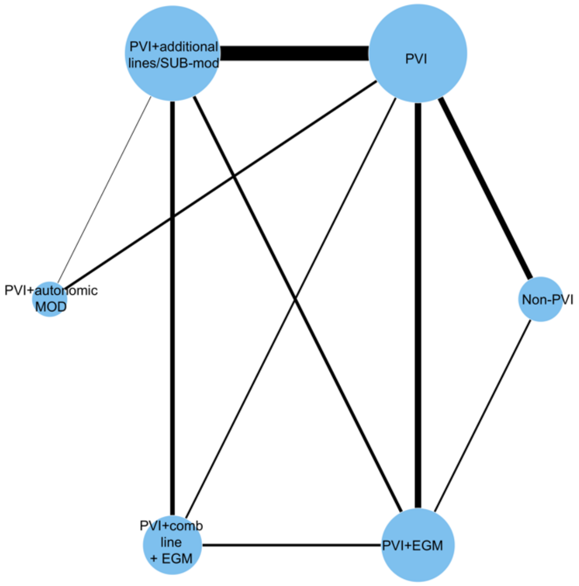

Heterogeneity τ^2=0

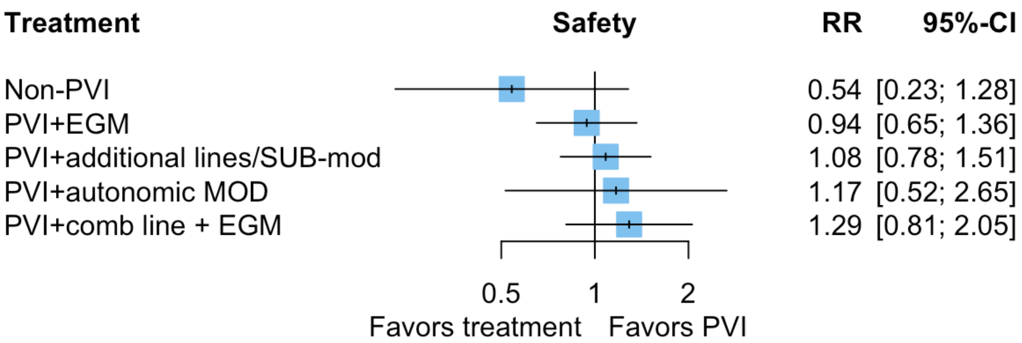

Procedural time:

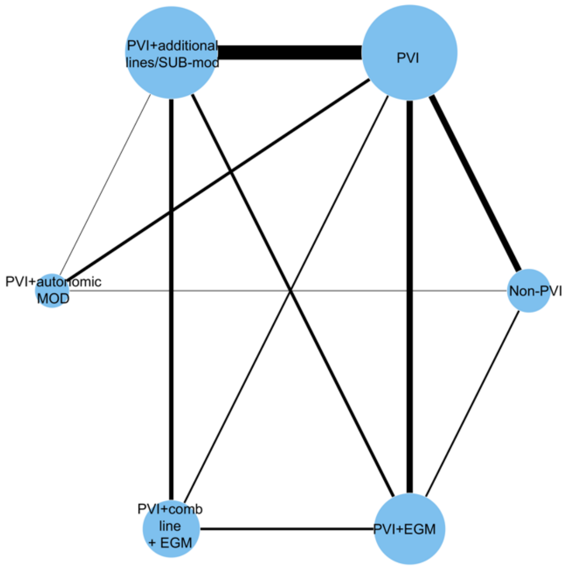

Heterogeneity τ^2=0.1031984

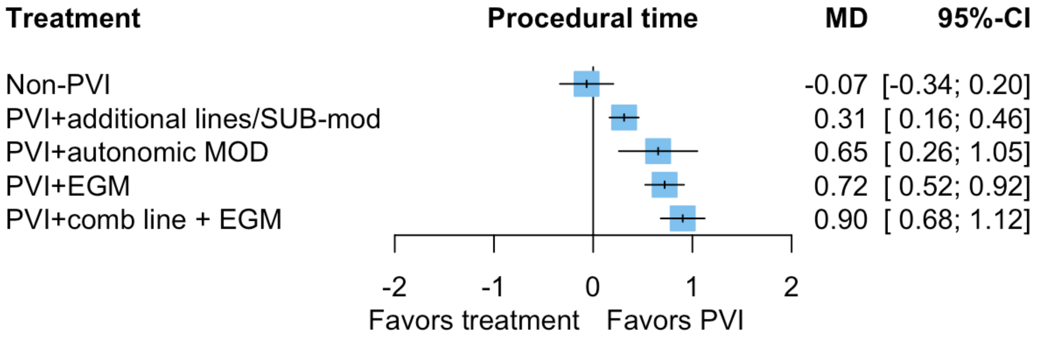

Supplement: Supplementary file 13 — Additional file 13. Sensitivity analyses. 1 Excluding high risk of bias RCTs (57 RCTs left). 2 Excluding RCTs with Renal Denervation (RDN) treatment (64 RCTs left). 3 Excluding RCTs with only PAF patients (42 RCTs left). 4 Excluding catheter 8mm, 8mm plus 3.5mm irrigated, 8mm and 4mm irrigated (55 RCTs left). 5 INCLUDING RCTs with antiarrhythmic drugs (AADs) as control arm (78 total RCTs). 6. Sensitivity analysis with reduced categories. [file 12916_2022_2385_MOESM13_ESM.pdf]
