# Supplementary material for: Comparing efficacy and safety in catheter ablation strategies for atrial fibrillation: a network meta-analysis
Source: BMC Med. 2022 May 31;20:193. doi: 10.1186/s12916-022-02385-2 (PMC9153169; doi:10.1186/s12916-022-02385-2)
Supplement: Supplementary file 14 — Additional file 14. Overall quality of the evidence with CINeMA assessment. Tables S1-S2. Table S1- [Confidence rating for efficacy using CINeMA]. Table S2- [Confidence rating for safety using CINeMA]. [file 12916_2022_2385_MOESM14_ESM.docx]

**Additional file 14. OVERALL QUALITY OF EVIDENCE WITH CINeMA ASSESSMENT**

Confidence in Network Meta-Analysis (CINeMA) tool (https://cinema.ispm. unibe.ch):

CINeMA is an online tool that allows for an automated evaluation of the quality of evidence of every comparison in the network. CINeMA considers six domains that may affect the level of confidence in the NMA results: (1) within‐study bias, (2) reporting bias, (3) indirectness, (4) imprecision, (5) heterogeneity, and (6) incoherence. For each relative treatment effect from NMA reviewers pick “no concerns,” “some concerns,” or “major concerns” in each of the six domains, which are then summarized into four confidence domains (“high,” “moderate,” “low,” or “very low”).

Specifically, the confidence in each NMA risk ratio, RR_XY_ (with two given drugs X and Y) was evaluated for all six domains. For each comparison and for each domain, the user is required to input his judgment to recommend whether there were 'major concerns', 'some concerns' or 'no concerns' for the particular comparison and domain. Thresholds and evaluation rules are typically decided through discussions.

For indirectness, judgments take into account the relative contributions of direct and indirect studies in the estimation. For imprecision, heterogeneity, and incoherence, the tool uses the concept of the minimally clinically important effect size and constructs the ‘range of equivalence’ between two interventions. Then, judgments about the three domains consider whether uncertainty intervals lie within this range.

1. Within‐trial bias: was estimated as the weighted average of the overall risk of bias of all the trials contributing information to the estimation of RRs.
2. Reporting bias (or 'publication bias'): was set to ‘low risk’ for all studies, as small-study effects assessment did not suggest the presence of publication bias and the risk of publication bias based on the knowledge of the clinical field did not raise any concerns.
3. Indirectness: Indirectness was summarized in each comparison as average for each relative effect estimate according to the percentage contribution of studies at each bias level.
4. Imprecision: we left the clinically important size of the effect (RR) to 1 as we had no clinical reason to assume otherwise.
5. Heterogeneity: was evaluated by monitoring agreement between confidence intervals (CIs) and prediction intervals (PIs) in terms of their ability to provide similar conclusions.
6. Incoherence: was evaluated by monitoring disagreement between confidence intervals (CIs) of the direct and indirect RR_XY_ and their overlap with the margin of equivalent effects.

We report the results of the evaluation of certainty of the evidence for the primary efficacy and safety outcomes in eTable 8 and eTable 9, as either no concern, some concerns, or major concerns for each domain assessed (within‐study bias, reporting bias, indirectness, imprecision, heterogeneity, and incoherence). For efficacy, we also report the contribution of medium or high risk of bias (RoB) comparisons to each network estimate and the contribution of indirectness.

Reasons for downgrading the evidence were related to the presence of concerns imprecision, within-study bias, indirectness and/or heterogeneity. However, the domains can often be considered jointly. For example, if some concerns are present in both indirectness and incoherence, we considered it sufficient to downgrade the evidence only by one level. This is because indirectness and incoherence are known to be related to one another. Assessment of indirectness of the research question includes considerations about transitivity, but on the other hand, transitivity concerns appear in the data in the form of statistical incoherence. Similarly, high heterogeneity increases imprecision, so these two domains are also interconnected. Evidence was downgraded by one level if “some concerns” were present in two or more related domains, whilst the presence of “major concerns” in a domain was considered enough to downgrade alone the evidence by one level. The two rules apply cumulatively and when jointly occurring can downgrade evidence by two or more levels.

**Table S1.** Confidence rating for efficacy using CINeMA

| **Comparison** | **Number of studies** | **Within-study bias** | **Reporting bias** | **Indirectness** | **Imprecision** | **Heterogeneity** | **Incoherence** | **Confidence rating** | **Reason(s) for downgrading** |
| --- | --- | --- | --- | --- | --- | --- | --- | --- | --- |
| **EGM:PVI** | 4 | Some concerns | Low risk | Some concerns | No concerns | Major concerns | No concerns | Low | [“Within-study bias”/”indirectness”]  ["Heterogeneity”] |
| **EGM:PVI_EGM** | 3 | Some concerns | Low risk | Some concerns | No concerns | No concerns | No concerns | Moderate | [“Within-study bias”/”indirectness”] |
| **GP:PVI** | 2 | Some concerns | Low risk | Some concerns | Major concerns | No concerns | No concerns | Low | [“Within-study bias”/”indirectness”]  Imprecision"] |
| **GP:PVI_GP** | 1 | Some concerns | Low risk | Some concerns | No concerns | Some concerns | No concerns | Moderate | [“Within-study bias”,“Heterogeneity,”Indirectness”] |
| **PVI:PVI_EGM** | 9 | Some concerns | Low risk | Some concerns | Some concerns | Some concerns | No concerns | Low | [“Within-study bias”,“Heterogeneity,”Indirectness”] |
| **PVI:PVI_GP** | 2 | Some concerns | Low risk | Some concerns | No concerns | Major concerns | No concerns | Low | ["Heterogeneity", “Within-study bias”] |
| **PVI:PVI_LAA** | 1 | Some concerns | Low risk | Some concerns | Major concerns | No concerns | No concerns | Low | ["Imprecision”]  [“Within-study bias”/”indirectness”] |
| **PVI:PVI_RDN** | 3 | Some concerns | Low risk | Major concerns | No concerns | Major concerns | No concerns | Low | ["Indirectness"] [Heterogeneity”] |
| **PVI:PVI_SVC_lines** | 4 | Some concerns | Low risk | No concerns | Major concerns | No concerns | No concerns | Moderate | ["Imprecision"] |
| **PVI:PVI_lines** | 15 | Some concerns | Low risk | Some concerns | No concerns | Major concerns | No concerns | Low | ["Heterogeneity"]  [“Within-study bias”/”indirectness”] |
| **PVI:PVI_partly** | 3 | Some concerns | Low risk | Some concerns | Major concerns | No concerns | No concerns | Low | ["Imprecision"]  [“Within-study bias”/”indirectness”] |
| **PVI:PVI_posterior_lines** | 6 | Some concerns | Low risk | Some concerns | Major concerns | No concerns | No concerns | Low | ["Imprecision"]  [“Within-study bias”/”indirectness”] |
| **PVI:PVI_step** | 3 | Some concerns | Low risk | Some concerns | Major concerns | No concerns | No concerns | Low | ["Imprecision"]  [“Within-study bias”/”indirectness”] |
| **PVI:Singlebox** | 2 | Some concerns | Low risk | Some concerns | Major concerns | No concerns | No concerns | Low | ["Imprecision"]  [“Within-study bias”/”indirectness”] |
| **PVI:Singlebox_lines** | 1 | Some concerns | Low risk | Some concerns | Major concerns | No concerns | No concerns | Low | ["Imprecision"]  [“Within-study bias”/”indirectness”] |
| **lines:PVI** | 1 | Some concerns | Low risk | Some concerns | Major concerns | No concerns | No concerns | Low | ["Imprecision"]  [“Within-study bias”/”indirectness”] |
| **PVI_BI_mod:PVI_lines** | 1 | Some concerns | Low risk | Some concerns | Major concerns | No concerns | No concerns | Low | ["Imprecision"]  [“Within-study bias”/”indirectness”] |
| **PVI_comb:PVI_EGM** | 3 | Some concerns | Low risk | Some concerns | Major concerns | No concerns | No concerns | Low | ["Imprecision"]  [“Within-study bias”/”indirectness”] |
| **PVI_EGM:PVI_lines** | 4 | Some concerns | Low risk | Some concerns | Major concerns | No concerns | No concerns | Low | ["Imprecision"]  [“Within-study bias”/”indirectness”] |
| **PVI_EGM:PVI_posterior_lines** | 2 | Some concerns | Low risk | Some concerns | Major concerns | No concerns | No concerns | Low | ["Imprecision"]  [“Within-study bias”/”indirectness”] |
| **PVI_EGM:PVI_trig** | 2 | Some concerns | Low risk | Some concerns | Major concerns | No concerns | No concerns | Low | ["Imprecision"]  [“Within-study bias”/”indirectness”] |
| **PVI_GP:PVI_lines** | 1 | Some concerns | Low risk | Some concerns | Major concerns | No concerns | No concerns | Low | ["Imprecision"]  [“Within-study bias”/”indirectness”] |
| **PVI_posterior_lines:PVI_SUB_mod** | 1 | Some concerns | Low risk | No concerns | Major concerns | No concerns | No concerns | Moderate | ["Imprecision"] |
| **PVI_step:PVI_SUB_mod** | 2 | Some concerns | Low risk | Major concerns | Major concerns | No concerns | No concerns | Low | ["Imprecision"] [“Indirectness”] |
| **PVI_comb:PVI_SVC_lines** | 1 | Some concerns | Low risk | No concerns | Major concerns | No concerns | No concerns | Moderate | ["Imprecision"] |
| **PVI_comb:PVI_lines** | 1 | Some concerns | Low risk | Some concerns | Major concerns | No concerns | No concerns | Low | ["Imprecision"]  [“Within-study bias”/”indirectness”] |
| **PVI_comb:PVI_posterior_lines** | 2 | Some concerns | Low risk | Some concerns | Major concerns | No concerns | No concerns | Low | ["Imprecision"]  [“Within-study bias”/”indirectness”] |
| **PVI_lines:PVI_posterior_lines** | 1 | Some concerns | Low risk | Some concerns | Major concerns | No concerns | No concerns | Low | ["Imprecision"]  [“Within-study bias”/”indirectness”] |
| **PVI_lines:PVI_step** | 1 | Some concerns | Low risk | Some concerns | No concerns | Major concerns | No concerns | Moderate | ["Heterogeneity"]  [“Within-study bias”/”indirectness”] |
| **PVI_lines:PVI_trig** | 1 | Some concerns | Low risk | Some concerns | Major concerns | No concerns | No concerns | Low | ["Imprecision"]  [“Within-study bias”/”indirectness”] |
| **PVI_lines:Singlebox** | 1 | Some concerns | Low risk | Some concerns | Major concerns | No concerns | No concerns | Low | ["Imprecision"]  [“Within-study bias”/”indirectness”] |
| **PVI_lines:Singlebox_lines** | 1 | Some concerns | Low risk | Some concerns | Major concerns | No concerns | No concerns | Low | ["Imprecision"]  [“Within-study bias”/”indirectness”] |
| **PVI_step:PVI_trig** | 1 | Some concerns | Low risk | Some concerns | Major concerns | No concerns | No concerns | Low | ["Imprecision"]  [“Within-study bias”/”indirectness”] |
| **Singlebox:Singlebox_lines** | 1 | Some concerns | Low risk | Some concerns | Major concerns | No concerns | No concerns | Low | ["Imprecision"]  [“Within-study bias”/”indirectness”] |
| **EGM:GP** | 0 | Some concerns | Low risk | Some concerns | Major concerns | No concerns | No concerns | Low | ["Imprecision"]  [“Within-study bias”/”indirectness”] |
| **EGM:PVI_BI_mod** | 0 | Some concerns | Low risk | Some concerns | No concerns | No concerns | No concerns | Moderate | [“Within-study bias”/”indirectness”] |
| **EGM:PVI_GP** | 0 | Some concerns | Low risk | Some concerns | No concerns | No concerns | No concerns | Moderate | [“Within-study bias”/”indirectness”] |
| **EGM:PVI_LAA** | 0 | Some concerns | Low risk | Some concerns | Major concerns | No concerns | No concerns | Low | ["Imprecision"]  [“Within-study bias”/”indirectness”] |
| **EGM:PVI_RDN** | 0 | Some concerns | Low risk | Some concerns | No concerns | No concerns | No concerns | Moderate | [“Within-study bias”/”indirectness”] |
| **EGM:PVI_SUB_mod** | 0 | Some concerns | Low risk | Some concerns | No concerns | Major concerns | No concerns | Low | ["Heterogeneity"]  [“Within-study bias”/”indirectness”] |
| **EGM:PVI_SVC_lines** | 0 | Some concerns | Low risk | Some concerns | No concerns | No concerns | No concerns | Moderate | [“Within-study bias”/”indirectness”] |
| **EGM:PVI_comb** | 0 | Some concerns | Low risk | Some concerns | No concerns | No concerns | No concerns | Moderate | [“Within-study bias”/”indirectness”] |
| **EGM:PVI_lines** | 0 | Some concerns | Low risk | Some concerns | No concerns | No concerns | No concerns | Moderate | [“Within-study bias”/”indirectness”] |
| **EGM:PVI_partly** | 0 | Some concerns | Low risk | Some concerns | Major concerns | No concerns | No concerns | Low | ["Imprecision"]  [“Within-study bias”/”indirectness”] |
| **EGM:PVI_posterior_lines** | 0 | Some concerns | Low risk | Some concerns | No concerns | No concerns | No concerns | Moderate | [“Within-study bias”/”indirectness”] |
| **EGM:PVI_step** | 0 | Some concerns | Low risk | Some concerns | No concerns | Major concerns | No concerns | Low | ["Heterogeneity"]  [“Within-study bias”/”indirectness”] |
| **EGM:PVI_trig** | 0 | Some concerns | Low risk | Some concerns | No concerns | Major concerns | No concerns | Low | ["Heterogeneity"]  [“Within-study bias”/”indirectness”] |
| **EGM:Singlebox** | 0 | Some concerns | Low risk | Some concerns | No concerns | Major concerns | No concerns | Low | ["Heterogeneity"]  [“Within-study bias”/”indirectness”] |
| **EGM:Singlebox_lines** | 0 | Some concerns | Low risk | Some concerns | No concerns | Major concerns | No concerns | Low | ["Heterogeneity"]  [“Within-study bias”/”indirectness”] |
| **EGM:lines** | 0 | Some concerns | Low risk | Some concerns | Major concerns | No concerns | No concerns | Low | ["Imprecision"]  [“Within-study bias”/”indirectness”] |
| **GP:PVI_BI_mod** | 0 | Some concerns | Low risk | Some concerns | No concerns | No concerns | No concerns | Moderate | [“Within-study bias”/”indirectness”] |
| **GP:PVI_EGM** | 0 | Some concerns | Low risk | Some concerns | No concerns | Major concerns | No concerns | Low | ["Heterogeneity"]  [“Within-study bias”/”indirectness”] |
| **GP:PVI_LAA** | 0 | Some concerns | Low risk | Some concerns | Major concerns | No concerns | No concerns | Low | ["Heterogeneity"]  [“Within-study bias”/”indirectness”] |
| **GP:PVI_RDN** | 0 | Some concerns | Low risk | Some concerns | No concerns | Some concerns | No concerns | Moderate | [“Within-study bias”/”indirectness”/ Heterogeneity] |
| **GP:PVI_SUB_mod** | 0 | Some concerns | Low risk | Some concerns | Major concerns | No concerns | No concerns | Low | ["Imprecision"]  [“Within-study bias”/”indirectness”] |
| **GP:PVI_SVC_lines** | 0 | Some concerns | Low risk | Some concerns | No concerns | Major concerns | No concerns | Low | ["heterogeneity"]  [“Within-study bias”/”indirectness”] |
| **GP:PVI_comb** | 0 | Some concerns | Low risk | Some concerns | Major concerns | No concerns | No concerns | Low | ["Imprecision"]  [“Within-study bias”/”indirectness”] |
| **GP:PVI_lines** | 0 | Some concerns | Low risk | Some concerns | No concerns | Major concerns | No concerns | Low | ["heterogeneity "]  [“Within-study bias”/”indirectness”] |
| **GP:PVI_partly** | 0 | Some concerns | Low risk | Some concerns | Major concerns | No concerns | No concerns | Low | ["Imprecision"]  [“Within-study bias”/”indirectness”] |
| **GP:PVI_posterior_lines** | 0 | Some concerns | Low risk | Some concerns | No concerns | Major concerns | No concerns | Low | ["heterogeneity "]  [“Within-study bias”/”indirectness”] |
| **GP:PVI_step** | 0 | Some concerns | Low risk | Some concerns | Major concerns | No concerns | No concerns | Low | ["Imprecision"]  [“Within-study bias”/”indirectness”] |
| **GP:PVI_trig** | 0 | Some concerns | Low risk | Some concerns | Major concerns | No concerns | No concerns | Low | ["Imprecision"]  [“Within-study bias”/”indirectness”] |
| **GP:Singlebox** | 0 | Some concerns | Low risk | Some concerns | Major concerns | No concerns | No concerns | Low | ["Imprecision"]  [“Within-study bias”/”indirectness”] |
| **GP:Singlebox_lines** | 0 | Some concerns | Low risk | Some concerns | Major concerns | No concerns | No concerns | Low | ["Imprecision"]  [“Within-study bias”/”indirectness”] |
| **GP:lines** | 0 | Some concerns | Low risk | Some concerns | Major concerns | No concerns | No concerns | Low | ["Imprecision"]  [“Within-study bias”/”indirectness”] |
| **PVI:PVI_BI_mod** | 0 | Some concerns | Low risk | Some concerns | No concerns | Major concerns | No concerns | Low | ["Heterogeneity"]  [“Within-study bias”/”indirectness”] |
| **PVI:PVI_SUB_mod** | 0 | Some concerns | Low risk | Some concerns | Major concerns | No concerns | No concerns | Low | ["Imprecision"]  [“Within-study bias”/”indirectness”] |
| **PVI:PVI_comb** | 0 | Some concerns | Low risk | Some concerns | Major concerns | No concerns | No concerns | Low | ["Imprecision"]  [“Within-study bias”/”indirectness”] |
| **PVI:PVI_trig** | 0 | Some concerns | Low risk | Some concerns | Major concerns | No concerns | No concerns | Low | ["Imprecision"]  [“Within-study bias”/”indirectness”] |
| **PVI_BI_mod:PVI_EGM** | 0 | Some concerns | Low risk | Some concerns | Major concerns | No concerns | No concerns | Low | ["Imprecision"]  [“Within-study bias”/”indirectness”] |
| **PVI_BI_mod:PVI_GP** | 0 | Some concerns | Low risk | Some concerns | Major concerns | No concerns | No concerns | Low | ["Imprecision"]  [“Within-study bias”/”indirectness”] |
| **PVI_BI_mod:PVI_LAA** | 0 | Some concerns | Low risk | Some concerns | No concerns | Major concerns | No concerns | Low | ["Heterogeneity"]  [“Within-study bias”/”indirectness”] |
| **PVI_BI_mod:PVI_RDN** | 0 | Some concerns | Low risk | Some concerns | Major concerns | No concerns | No concerns | Low | ["Imprecision"]  [“Within-study bias”/”indirectness”] |
| **PVI_BI_mod:PVI_SUB_mod** | 0 | Some concerns | Low risk | Some concerns | Some concerns | Some concerns | No concerns | Low | [“Within-study bias”,“Heterogeneity,”Indirectness”, Imprecision] |
| **PVI_BI_mod:PVI_SVC_lines** | 0 | Some concerns | Low risk | Some concerns | Major concerns | No concerns | No concerns | Low | ["Imprecision"]  [“Within-study bias”/”indirectness”] |
| **PVI_BI_mod:PVI_comb** | 0 | Some concerns | Low risk | Some concerns | Major concerns | No concerns | No concerns | Low | ["Imprecision"]  [“Within-study bias”/”indirectness”] |
| **PVI_BI_mod:PVI_partly** | 0 | Some concerns | Low risk | Some concerns | No concerns | Some concerns | No concerns | Moderate | [“Indirectness”/"Heterogeneity"/ Within-study] |
| **PVI_BI_mod:PVI_posterior_lines** | 0 | Some concerns | Low risk | Some concerns | Major concerns | No concerns | No concerns | Low | ["Imprecision"]  [“Within-study bias”/”indirectness”] |
| **PVI_BI_mod:PVI_step** | 0 | Some concerns | Low risk | Some concerns | No concerns | No concerns | No concerns | Moderate | [“Indirectness”/Within-study] |
| **PVI_BI_mod:PVI_trig** | 0 | Some concerns | Low risk | Some concerns | Major concerns | No concerns | No concerns | Low | ["Imprecision"]  [“Within-study bias”/”indirectness”] |
| **PVI_BI_mod:Singlebox** | 0 | Some concerns | Low risk | Some concerns | Major concerns | No concerns | No concerns | Low | ["Imprecision"]  [“Within-study bias”/”indirectness”] |
| **PVI_BI_mod:Singlebox_lines** | 0 | Some concerns | Low risk | Some concerns | Major concerns | No concerns | No concerns | Low | ["Imprecision"]  [“Within-study bias”/”indirectness”] |
| **lines:PVI_BI_mod** | 0 | Some concerns | Low risk | Some concerns | No concerns | No concerns | No concerns | Low | ["Imprecision"]  [“Within-study bias”/”indirectness”] |
| **PVI_EGM:PVI_GP** | 0 | Some concerns | Low risk | Some concerns | Major concerns | No concerns | No concerns | Low | ["Imprecision"]  [“Within-study bias”/”indirectness”] |
| **PVI_EGM:PVI_LAA** | 0 | Some concerns | Low risk | Some concerns | Major concerns | No concerns | No concerns | Low | ["Imprecision"]  [“Within-study bias”/”indirectness”] |
| **PVI_EGM:PVI_RDN** | 0 | Some concerns | Low risk | Some concerns | Major concerns | No concerns | No concerns | Low | ["Imprecision"]  [“Within-study bias”/”indirectness”] |
| **PVI_EGM:PVI_SUB_mod** | 0 | Some concerns | Low risk | Some concerns | Major concerns | No concerns | No concerns | Low | ["Imprecision"]  [“Within-study bias”/”indirectness”] |
| **PVI_EGM:PVI_SVC_lines** | 0 | Some concerns | Low risk | Some concerns | Major concerns | No concerns | No concerns | Low | ["Imprecision"]  [“Within-study bias”/”indirectness”] |
| **PVI_EGM:PVI_partly** | 0 | Some concerns | Low risk | Some concerns | Major concerns | No concerns | No concerns | Low | ["Imprecision"]  [“Within-study bias”/”indirectness”] |
| **PVI_EGM:PVI_step** | 0 | Some concerns | Low risk | Some concerns | Major concerns | No concerns | No concerns | Low | ["Imprecision"]  [“Within-study bias”/”indirectness”] |
| **PVI_EGM:Singlebox** | 0 | Some concerns | Low risk | Some concerns | Major concerns | No concerns | No concerns | Low | ["Imprecision"]  [“Within-study bias”/”indirectness”] |
| **PVI_EGM:Singlebox_lines** | 0 | Some concerns | Low risk | Some concerns | Major concerns | No concerns | No concerns | Low | ["Imprecision"]  [“Within-study bias”/”indirectness”] |
| **lines:PVI_EGM** | 0 | Some concerns | Low risk | Some concerns | Major concerns | No concerns | No concerns | Low | ["Imprecision"]  [“Within-study bias”/”indirectness”] |
| **PVI_GP:PVI_LAA** | 0 | Some concerns | Low risk | Some concerns | Major concerns | No concerns | No concerns | Low | ["Imprecision"]  [“Within-study bias”/”indirectness”] |
| **PVI_GP:PVI_RDN** | 0 | Some concerns | Low risk | Some concerns | Major concerns | No concerns | No concerns | Low | ["Imprecision"]  [“Within-study bias”/”indirectness”] |
| **PVI_GP:PVI_SUB_mod** | 0 | Some concerns | Low risk | Some concerns | Major concerns | No concerns | No concerns | Low | ["Imprecision"]  [“Within-study bias”/”indirectness”] |
| **PVI_GP:PVI_SVC_lines** | 0 | Some concerns | Low risk | Some concerns | Major concerns | No concerns | No concerns | Low | ["Imprecision"]  [“Within-study bias”/”indirectness”] |
| **PVI_comb:PVI_GP** | 0 | Some concerns | Low risk | Some concerns | Major concerns | No concerns | No concerns | Low | ["Imprecision"]  [“Within-study bias”/”indirectness”] |
| **PVI_GP:PVI_partly** | 0 | Some concerns | Low risk | Some concerns | Some concerns | Some concerns | No concerns | Low | ["Imprecision"]  [“Within-study bias”/”indirectness”] |
| **PVI_GP:PVI_posterior_lines** | 0 | Some concerns | Low risk | Some concerns | Major concerns | No concerns | No concerns | Low | ["Imprecision"]  [“Within-study bias”/”indirectness”] |
| **PVI_GP:PVI_step** | 0 | Some concerns | Low risk | Some concerns | No concerns | Major concerns | No concerns | Low | ["Imprecision"]  [“Within-study bias”/”indirectness”] |
| **PVI_GP:PVI_trig** | 0 | Some concerns | Low risk | Some concerns | Major concerns | No concerns | No concerns | Low | ["Imprecision"]  [“Within-study bias”/”indirectness”] |
| **PVI_GP:Singlebox** | 0 | Some concerns | Low risk | Some concerns | Major concerns | No concerns | No concerns | Low | ["Imprecision"]  [“Within-study bias”/”indirectness”] |
| **PVI_GP:Singlebox_lines** | 0 | Some concerns | Low risk | Some concerns | Major concerns | No concerns | No concerns | Low | ["Imprecision"]  [“Within-study bias”/”indirectness”] |
| **lines:PVI_GP** | 0 | Some concerns | Low risk | Some concerns | Major concerns | No concerns | No concerns | Low | ["Imprecision"]  [“Within-study bias”/”indirectness”] |
| **PVI_LAA:PVI_RDN** | 0 | Some concerns | Low risk | Some concerns | Major concerns | No concerns | No concerns | Low | ["Imprecision"]  [“Within-study bias”/”indirectness”] |
| **PVI_LAA:PVI_SUB_mod** | 0 | Some concerns | Low risk | Some concerns | Major concerns | No concerns | No concerns | Low | ["Imprecision"]  [“Within-study bias”/”indirectness”] |
| **PVI_LAA:PVI_SVC_lines** | 0 | Some concerns | Low risk | Some concerns | Major concerns | No concerns | No concerns | Low | ["Imprecision"]  [“Within-study bias”/”indirectness”] |
| **PVI_comb:PVI_LAA** | 0 | Some concerns | Low risk | Some concerns | Major concerns | No concerns | No concerns | Low | ["Imprecision"]  [“Within-study bias”/”indirectness”] |
| **PVI_LAA:PVI_lines** | 0 | Some concerns | Low risk | Some concerns | Major concerns | No concerns | No concerns | Low | ["Imprecision"]  [“Within-study bias”/”indirectness”] |
| **PVI_LAA:PVI_partly** | 0 | Some concerns | Low risk | Some concerns | Major concerns | No concerns | No concerns | Low | ["Imprecision"]  [“Within-study bias”/”indirectness”] |
| **PVI_LAA:PVI_posterior_lines** | 0 | Some concerns | Low risk | Some concerns | Major concerns | No concerns | No concerns | Low | ["Imprecision"]  [“Within-study bias”/”indirectness”] |
| **PVI_LAA:PVI_step** | 0 | Some concerns | Low risk | Some concerns | Major concerns | No concerns | No concerns | Low | ["Imprecision"]  [“Within-study bias”/”indirectness”] |
| **PVI_LAA:PVI_trig** | 0 | Some concerns | Low risk | Some concerns | Major concerns | No concerns | No concerns | Low | ["Imprecision"]  [“Within-study bias”/”indirectness”] |
| **PVI_LAA:Singlebox** | 0 | Some concerns | Low risk | Some concerns | Major concerns | No concerns | No concerns | Low | ["Imprecision"]  [“Within-study bias”/”indirectness”] |
| **PVI_LAA:Singlebox_lines** | 0 | Some concerns | Low risk | Some concerns | Major concerns | No concerns | No concerns | Low | ["Imprecision"]  [“Within-study bias”/”indirectness”] |
| **lines:PVI_LAA** | 0 | Some concerns | Low risk | Some concerns | Major concerns | No concerns | No concerns | Low | ["Imprecision"]  [“Within-study bias”/”indirectness”] |
| **PVI_RDN:PVI_SUB_mod** | 0 | Some concerns | Low risk | Major concerns | Major concerns | No concerns | No concerns | Low | [“Indirectness”]  [“Imprecision"] |
| **PVI_RDN:PVI_SVC_lines** | 0 | Some concerns | Low risk | Major concerns | Major concerns | No concerns | No concerns | Low | [“Indirectness”]  [“Imprecision"] |
| **PVI_comb:PVI_RDN** | 0 | Some concerns | Low risk | Some concerns | Major concerns | No concerns | No concerns | Low | ["Imprecision"]  [“Within-study bias”/”indirectness”] |
| **PVI_lines:PVI_RDN** | 0 | Some concerns | Low risk | Some concerns | Major concerns | No concerns | No concerns | Low | ["Imprecision"]  [“Within-study bias”/”indirectness”] |
| **PVI_partly:PVI_RDN** | 0 | Some concerns | Low risk | Some concerns | Some concerns | Some concerns | No concerns | Moderate | ["Imprecision" or [“Indirectness or ‘Heterogeneity’] |
| **PVI_posterior_lines:PVI_RDN** | 0 | Some concerns | Low risk | Some concerns | Major concerns | No concerns | No concerns | Low | ["Imprecision"]  [“Within-study bias”/”indirectness”] |
| **PVI_RDN:PVI_step** | 0 | Some concerns | Low risk | Major concerns | No concerns | Major concerns | No concerns | Low | ["Indirectness”], [“Heterogeneity"] |
| **PVI_RDN:PVI_trig** | 0 | Some concerns | Low risk | Some concerns | Major concerns | No concerns | No concerns | Low | ["Imprecision"]  [“Within-study bias”/”indirectness”] |
| **PVI_RDN:Singlebox** | 0 | Some concerns | Low risk | Some concerns | Major concerns | No concerns | No concerns | Low | ["Imprecision"]  [“Within-study bias”/”indirectness”] |
| **PVI_RDN:Singlebox_lines** | 0 | Some concerns | Low risk | Some concerns | Major concerns | No concerns | No concerns | Low | ["Imprecision"]  [“Within-study bias”/”indirectness”] |
| **lines:PVI_RDN** | 0 | Some concerns | Low risk | Some concerns | Major concerns | No concerns | No concerns | Low | ["Imprecision"]  [“Within-study bias”/”indirectness”] |
| **PVI_SUB_mod:PVI_SVC_lines** | 0 | Some concerns | Low risk | No concerns | Major concerns | No concerns | No concerns | Moderate | ["Imprecision"] |
| **PVI_comb:PVI_SUB_mod** | 0 | Some concerns | Low risk | Some concerns | Major concerns | No concerns | No concerns | Low | ["Imprecision"]  [“Within-study bias”/”indirectness”] |
| **PVI_lines:PVI_SUB_mod** | 0 | Some concerns | Low risk | Some concerns | Major concerns | No concerns | No concerns | Low | ["Imprecision"]  [“Within-study bias”/”indirectness”] |
| **PVI_partly:PVI_SUB_mod** | 0 | Some concerns | Low risk | Some concerns | Major concerns | No concerns | No concerns | Low | ["Imprecision"]  [“Within-study bias”/”indirectness”] |
| **PVI_SUB_mod:PVI_trig** | 0 | Some concerns | Low risk | Some concerns | Major concerns | No concerns | No concerns | Low | ["Imprecision"]  [“Within-study bias”/”indirectness”] |
| **PVI_SUB_mod:Singlebox** | 0 | Some concerns | Low risk | Some concerns | Major concerns | No concerns | No concerns | Low | ["Imprecision"]  [“Within-study bias”/”indirectness”] |
| **PVI_SUB_mod:Singlebox_lines** | 0 | Some concerns | Low risk | Some concerns | Major concerns | No concerns | No concerns | Low | ["Imprecision"]  [“Within-study bias”/”indirectness”] |
| **lines:PVI_SUB_mod** | 0 | Some concerns | Low risk | Some concerns | Major concerns | No concerns | No concerns | Low | ["Imprecision"]  [“Within-study bias”/”indirectness”] |
| **PVI_lines:PVI_SVC_lines** | 0 | Some concerns | Low risk | Some concerns | Major concerns | No concerns | No concerns | Low | ["Imprecision"]  [“Within-study bias”/”indirectness”] |
| **PVI_partly:PVI_SVC_lines** | 0 | Some concerns | Low risk | Some concerns | Major concerns | No concerns | No concerns | Low | ["Imprecision"]  [“Within-study bias”/”indirectness”] |
| **PVI_posterior_lines:PVI_SVC_lines** | 0 | Some concerns | Low risk | Some concerns | Major concerns | No concerns | No concerns | Low | ["Imprecision"]  [“Within-study bias”/”indirectness”] |
| **PVI_step:PVI_SVC_lines** | 0 | Some concerns | Low risk | Some concerns | Major concerns | No concerns | No concerns | Low | ["Imprecision"]  [“Within-study bias”/”indirectness”] |
| **PVI_SVC_lines:PVI_trig** | 0 | Some concerns | Low risk | Some concerns | Major concerns | No concerns | No concerns | Low | ["Imprecision"]  [“Within-study bias”/”indirectness”] |
| **PVI_SVC_lines:Singlebox** | 0 | Some concerns | Low risk | Some concerns | Major concerns | No concerns | No concerns | Low | ["Imprecision"]  [“Within-study bias”/”indirectness”] |
| **PVI_SVC_lines:Singlebox_lines** | 0 | Some concerns | Low risk | Some concerns | Major concerns | No concerns | No concerns | Low | ["Imprecision"]  [“Within-study bias”/”indirectness”] |
| **lines:PVI_SVC_lines** | 0 | Some concerns | Low risk | Some concerns | Major concerns | No concerns | No concerns | Low | ["Imprecision"]  [“Within-study bias”/”indirectness”] |
| **PVI_comb:PVI_partly** | 0 | Some concerns | Low risk | Some concerns | Major concerns | No concerns | No concerns | Low | ["Imprecision"]  [“Within-study bias”/”indirectness”] |
| **PVI_comb:PVI_step** | 0 | Some concerns | Low risk | Some concerns | Major concerns | No concerns | No concerns | Low | ["Imprecision"]  [“Within-study bias”/”indirectness”] |
| **PVI_comb:PVI_trig** | 0 | Some concerns | Low risk | Some concerns | Major concerns | No concerns | No concerns | Low | ["Imprecision"]  [“Within-study bias”/”indirectness”] |
| **PVI_comb:Singlebox** | 0 | Some concerns | Low risk | Some concerns | Major concerns | No concerns | No concerns | Low | ["Imprecision"]  [“Within-study bias”/”indirectness”] |
| **PVI_comb:Singlebox_lines** | 0 | Some concerns | Low risk | Some concerns | Major concerns | No concerns | No concerns | Low | ["Imprecision"]  [“Within-study bias”/”indirectness”] |
| **lines:PVI_comb** | 0 | Some concerns | Low risk | Some concerns | Major concerns | No concerns | No concerns | Low | ["Imprecision"]  [“Within-study bias”/”indirectness”] |
| **PVI_lines:PVI_partly** | 0 | Some concerns | Low risk | Some concerns | Major concerns | No concerns | No concerns | Low | ["Imprecision"]  [“Within-study bias”/”indirectness”] |
| **lines:PVI_lines** | 0 | Some concerns | Low risk | Some concerns | Major concerns | No concerns | No concerns | Low | ["Imprecision"]  [“Within-study bias”/”indirectness”] |
| **PVI_partly:PVI_posterior_lines** | 0 | Some concerns | Low risk | Some concerns | Major concerns | No concerns | No concerns | Low | ["Imprecision"]  [“Within-study bias”/”indirectness”] |
| **PVI_partly:PVI_step** | 0 | Some concerns | Low risk | Some concerns | Major concerns | No concerns | No concerns | Low | ["Imprecision"]  [“Within-study bias”/”indirectness”] |
| **PVI_partly:PVI_trig** | 0 | Some concerns | Low risk | Some concerns | Major concerns | No concerns | No concerns | Low | ["Imprecision"]  [“Within-study bias”/”indirectness”] |
| **PVI_partly:Singlebox** | 0 | Some concerns | Low risk | Some concerns | Major concerns | No concerns | No concerns | Low | ["Imprecision"]  [“Within-study bias”/”indirectness”] |
| **PVI_partly:Singlebox_lines** | 0 | Some concerns | Low risk | Some concerns | Major concerns | No concerns | No concerns | Low | ["Imprecision"]  [“Within-study bias”/”indirectness”] |
| **lines:PVI_partly** | 0 | Some concerns | Low risk | Some concerns | Major concerns | No concerns | No concerns | Low | ["Imprecision"]  [“Within-study bias”/”indirectness”] |
| **PVI_posterior_lines:PVI_step** | 0 | Some concerns | Low risk | Some concerns | Major concerns | No concerns | No concerns | Low | ["Imprecision"]  [“Within-study bias”/”indirectness”] |
| **PVI_posterior_lines:PVI_trig** | 0 | Some concerns | Low risk | Some concerns | Major concerns | No concerns | No concerns | Low | ["Imprecision"]  [“Within-study bias”/”indirectness”] |
| **PVI_posterior_lines:Singlebox** | 0 | Some concerns | Low risk | Some concerns | Major concerns | No concerns | No concerns | Low | ["Imprecision"]  [“Within-study bias”/”indirectness”] |
| **PVI_posterior_lines:Singlebox_lines** | 0 | Some concerns | Low risk | Some concerns | Major concerns | No concerns | No concerns | Low | ["Imprecision"]  [“Within-study bias”/”indirectness”] |
| **lines:PVI_posterior_lines** | 0 | Some concerns | Low risk | Some concerns | Major concerns | No concerns | No concerns | Low | ["Imprecision"]  [“Within-study bias”/”indirectness”] |
| **PVI_step:Singlebox** | 0 | Some concerns | Low risk | Some concerns | Major concerns | No concerns | No concerns | Low | ["Imprecision"]  [“Within-study bias”/”indirectness”] |
| **PVI_step:Singlebox_lines** | 0 | Some concerns | Low risk | Some concerns | Major concerns | No concerns | No concerns | Low | ["Imprecision"]  [“Within-study bias”/”indirectness”] |
| **lines:PVI_step** | 0 | Some concerns | Low risk | Some concerns | Major concerns | No concerns | No concerns | Low | ["Imprecision"]  [“Within-study bias”/”indirectness”] |
| **PVI_trig:Singlebox** | 0 | Some concerns | Low risk | Some concerns | Major concerns | No concerns | No concerns | Low | ["Imprecision"]  [“Within-study bias”/”indirectness”] |
| **PVI_trig:Singlebox_lines** | 0 | Some concerns | Low risk | Some concerns | Major concerns | No concerns | No concerns | Low | ["Imprecision"]  [“Within-study bias”/”indirectness”] |
| **lines:PVI_trig** | 0 | Some concerns | Low risk | Some concerns | Major concerns | No concerns | No concerns | Low | ["Imprecision"]  [“Within-study bias”/”indirectness”] |
| **lines:Singlebox** | 0 | Some concerns | Low risk | Some concerns | Major concerns | No concerns | No concerns | Low | ["Imprecision"]  [“Within-study bias”/”indirectness”] |
| **lines:Singlebox_lines** | 0 | Some concerns | Low risk | Some concerns | Major concerns | No concerns | No concerns | Low | ["Imprecision"]  [“Within-study bias”/”indirectness”] |

**Table S2** Confidence rating for safety using CINeMA

| **Comparison** | **Number of studies** | **Within-study bias** | **Reporting bias** | **Indirectness** | **Imprecision** | **Heterogeneity** | **Incoherence** | **Confidence rating** | **Reason(s) for downgrading** |
| --- | --- | --- | --- | --- | --- | --- | --- | --- | --- |
| **EGM:PVI** | 3 | Some concerns | Low risk | Some concerns | Major concerns | No concerns | No concerns | Low | ["Imprecision"]  [“Within-study bias”/”indirectness”] |
| **EGM:PVI_EGM** | 2 | Some concerns | Low risk | Some concerns | Major concerns | No concerns | No concerns | Low | ["Imprecision"]  [“Within-study bias”/”indirectness”] |
| **GP:PVI** | 1 | Some concerns | Low risk | Some concerns | Major concerns | No concerns | No concerns | Low | ["Imprecision"]  [“Within-study bias”/”indirectness”] |
| **GP:PVI_GP** | 1 | Some concerns | Low risk | Some concerns | Major concerns | No concerns | No concerns | Low | ["Imprecision"]  [“Within-study bias”/”indirectness”] |
| **PVI:PVI_EGM** | 8 | Some concerns | Low risk | Some concerns | Major concerns | No concerns | No concerns | Low | ["Imprecision"]  [“Within-study bias”/”indirectness”] |
| **PVI:PVI_GP** | 2 | Some concerns | Low risk | Some concerns | Major concerns | No concerns | No concerns | Low | ["Imprecision"]  [“Within-study bias”/”indirectness”] |
| **PVI:PVI_LAA** | 1 | Some concerns | Low risk | Some concerns | Major concerns | No concerns | No concerns | Low | ["Imprecision"]  [“Within-study bias”/”indirectness”] |
| **PVI:PVI_RDN** | 3 | Some concerns | Low risk | Major concerns | Major concerns | No concerns | No concerns | Low | ["Imprecision"]  [“Within-study bias”/”indirectness”] |
| **PVI:PVI_SVC_lines** | 4 | Some concerns | Low risk | Some concerns | Major concerns | No concerns | No concerns | Low | ["Imprecision"]  [“Within-study bias”/”indirectness”] |
| **PVI:PVI_lines** | 11 | Some concerns | Low risk | Some concerns | Major concerns | No concerns | No concerns | Low | ["Imprecision"]  [“Within-study bias”/”indirectness”] |
| **PVI:PVI_partly** | 3 | Some concerns | Low risk | Some concerns | Major concerns | No concerns | No concerns | Low | ["Imprecision"]  [“Within-study bias”/”indirectness”] |
| **PVI:PVI_posterior_lines** | 6 | Some concerns | Low risk | Some concerns | Major concerns | No concerns | No concerns | Low | ["Imprecision"]  [“Within-study bias”/”indirectness”] |
| **PVI:PVI_step** | 2 | Some concerns | Low risk | Some concerns | Major concerns | No concerns | No concerns | Low | ["Imprecision"]  [“Within-study bias”/”indirectness”] |
| **PVI:Singlebox** | 1 | Some concerns | Low risk | No concerns | Major concerns | No concerns | No concerns | Low | ["Imprecision"]  [“Within-study bias”/”indirectness”] |
| **lines:PVI** | 1 | Some concerns | Low risk | Some concerns | Major concerns | No concerns | No concerns | Low | ["Imprecision"]  [“Within-study bias”/”indirectness”] |
| **PVI_BI_mod:PVI_lines** | 1 | Some concerns | Low risk | Some concerns | Major concerns | No concerns | No concerns | Low | ["Imprecision"]  [“Within-study bias”/”indirectness”] |
| **PVI_comb:PVI_EGM** | 2 | Some concerns | Low risk | Major concerns | Major concerns | No concerns | No concerns | Low | ["Indirectness"]  ["Imprecision"] |
| **PVI_EGM:PVI_lines** | 4 | Some concerns | Low risk | Some concerns | Major concerns | No concerns | No concerns | Low | ["Imprecision"]  [“Within-study bias”/”indirectness”] |
| **PVI_EGM:PVI_posterior_lines** | 2 | Some concerns | Low risk | Some concerns | Major concerns | No concerns | No concerns | Low | ["Imprecision"]  [“Within-study bias”/”indirectness”] |
| **PVI_EGM:PVI_trig** | 2 | Some concerns | Low risk | Some concerns | Major concerns | No concerns | No concerns | Low | ["Imprecision"]  [“Within-study bias”/”indirectness”] |
| **PVI_GP:PVI_lines** | 1 | Some concerns | Low risk | Some concerns | Major concerns | No concerns | No concerns | Low | ["Imprecision"]  [“Within-study bias”/”indirectness”] |
| **PVI_posterior_lines:PVI_SUB_mod** | 1 | Some concerns | Low risk | No concerns | Major concerns | No concerns | No concerns | Moderate | ["Imprecision"] |
| **PVI_step:PVI_SUB_mod** | 2 | Some concerns | Low risk | Some concerns | Major concerns | No concerns | No concerns | Low | ["Imprecision"]  [“Within-study bias”/”indirectness”] |
| **PVI_comb:PVI_SVC_lines** | 1 | Some concerns | Low risk | Some concerns | Major concerns | No concerns | No concerns | Low | ["Imprecision"]  [“Within-study bias”/”indirectness”] |
| **PVI_comb:PVI_posterior_lines** | 2 | Some concerns | Low risk | Major concerns | Major concerns | No concerns | No concerns | Low | ["Indirectness"]  ["Imprecision"] |
| **PVI_lines:PVI_posterior_lines** | 1 | Some concerns | Low risk | Some concerns | Major concerns | No concerns | No concerns | Low | ["Imprecision"]  [“Within-study bias”/”indirectness”] |
| **PVI_lines:PVI_step** | 1 | Some concerns | Low risk | Some concerns | Major concerns | No concerns | No concerns | Low | ["Imprecision"]  [“Within-study bias”/”indirectness”] |
| **PVI_lines:PVI_trig** | 1 | Some concerns | Low risk | Some concerns | Major concerns | No concerns | No concerns | Low | ["Imprecision"]  [“Within-study bias”/”indirectness”] |
| **PVI_step:PVI_trig** | 1 | Some concerns | Low risk | Some concerns | Major concerns | No concerns | No concerns | Low | ["Imprecision"]  [“Within-study bias”/”indirectness”] |
| **EGM:GP** | 0 | Some concerns | Low risk | Some concerns | Major concerns | No concerns | No concerns | Low | ["Imprecision"]  [“Within-study bias”/”indirectness”] |
| **EGM:PVI_BI_mod** | 0 | Some concerns | Low risk | Some concerns | Major concerns | No concerns | No concerns | Low | ["Imprecision"]  [“Within-study bias”/”indirectness”] |
| **EGM:PVI_GP** | 0 | Some concerns | Low risk | Some concerns | Major concerns | No concerns | No concerns | Low | ["Imprecision"]  [“Within-study bias”/”indirectness”] |
| **EGM:PVI_LAA** | 0 | Some concerns | Low risk | Some concerns | Major concerns | No concerns | No concerns | Low | ["Imprecision"]  [“Within-study bias”/”indirectness”] |
| **EGM:PVI_RDN** | 0 | Some concerns | Low risk | Some concerns | Major concerns | No concerns | No concerns | Low | ["Imprecision"]  [“Within-study bias”/”indirectness”] |
| **EGM:PVI_SUB_mod** | 0 | Some concerns | Low risk | Some concerns | Major concerns | No concerns | No concerns | Low | ["Imprecision"]  [“Within-study bias”/”indirectness”] |
| **EGM:PVI_SVC_lines** | 0 | Some concerns | Low risk | Some concerns | Major concerns | No concerns | No concerns | Low | ["Imprecision"]  [“Within-study bias”/”indirectness”] |
| **EGM:PVI_comb** | 0 | Some concerns | Low risk | Some concerns | Major concerns | No concerns | No concerns | Low | ["Imprecision"]  [“Within-study bias”/”indirectness”] |
| **EGM:PVI_lines** | 0 | Some concerns | Low risk | Some concerns | Major concerns | No concerns | No concerns | Low | ["Imprecision"]  [“Within-study bias”/”indirectness”] |
| **EGM:PVI_partly** | 0 | Some concerns | Low risk | Some concerns | Major concerns | No concerns | No concerns | Low | ["Imprecision"]  [“Within-study bias”/”indirectness”] |
| **EGM:PVI_posterior_lines** | 0 | Some concerns | Low risk | Some concerns | Major concerns | No concerns | No concerns | Low | ["Imprecision"]  [“Within-study bias”/”indirectness”] |
| **EGM:PVI_step** | 0 | Some concerns | Low risk | Some concerns | No concerns | No concerns | No concerns | Moderate | ["Indirectness"] |
| **EGM:PVI_trig** | 0 | Some concerns | Low risk | Some concerns | Major concerns | No concerns | No concerns | Low | ["Imprecision"]  [“Within-study bias”/”indirectness”] |
| **EGM:Singlebox** | 0 | Some concerns | Low risk | Some concerns | Major concerns | No concerns | No concerns | Low | ["Imprecision"]  [“Within-study bias”/”indirectness”] |
| **EGM:lines** | 0 | Some concerns | Low risk | Some concerns | Major concerns | No concerns | No concerns | Low | ["Imprecision"]  [“Within-study bias”/”indirectness”] |
| **GP:PVI_BI_mod** | 0 | Some concerns | Low risk | Some concerns | Major concerns | No concerns | No concerns | Low | ["Imprecision"]  [“Within-study bias”/”indirectness”] |
| **GP:PVI_EGM** | 0 | Some concerns | Low risk | Some concerns | Major concerns | No concerns | No concerns | Low | ["Imprecision"]  [“Within-study bias”/”indirectness”] |
| **GP:PVI_LAA** | 0 | Some concerns | Low risk | Some concerns | Major concerns | No concerns | No concerns | Low | ["Imprecision"]  [“Within-study bias”/”indirectness”] |
| **GP:PVI_RDN** | 0 | Some concerns | Low risk | Some concerns | Major concerns | No concerns | No concerns | Low | ["Imprecision"]  [“Within-study bias”/”indirectness”] |
| **GP:PVI_SUB_mod** | 0 | Some concerns | Low risk | Some concerns | Major concerns | No concerns | No concerns | Low | ["Imprecision"]  [“Within-study bias”/”indirectness”] |
| **GP:PVI_SVC_lines** | 0 | Some concerns | Low risk | Some concerns | Major concerns | No concerns | No concerns | Low | ["Imprecision"]  [“Within-study bias”/”indirectness”] |
| **GP:PVI_comb** | 0 | Some concerns | Low risk | Some concerns | Major concerns | No concerns | No concerns | Low | ["Imprecision"]  [“Within-study bias”/”indirectness”] |
| **GP:PVI_lines** | 0 | Some concerns | Low risk | Some concerns | Major concerns | No concerns | No concerns | Low | ["Imprecision"]  [“Within-study bias”/”indirectness”] |
| **GP:PVI_partly** | 0 | Some concerns | Low risk | Some concerns | Major concerns | No concerns | No concerns | Low | ["Imprecision"]  [“Within-study bias”/”indirectness”] |
| **GP:PVI_posterior_lines** | 0 | Some concerns | Low risk | Some concerns | Major concerns | No concerns | No concerns | Low | ["Imprecision"]  [“Within-study bias”/”indirectness”] |
| **GP:PVI_step** | 0 | Some concerns | Low risk | Some concerns | Major concerns | No concerns | No concerns | Low | ["Imprecision"]  [“Within-study bias”/”indirectness”] |
| **GP:PVI_trig** | 0 | Some concerns | Low risk | Some concerns | Major concerns | No concerns | No concerns | Low | ["Imprecision"]  [“Within-study bias”/”indirectness”] |
| **GP:Singlebox** | 0 | Some concerns | Low risk | Some concerns | Major concerns | No concerns | No concerns | Low | ["Imprecision"]  [“Within-study bias”/”indirectness”] |
| **GP:lines** | 0 | Some concerns | Low risk | Some concerns | Major concerns | No concerns | No concerns | Low | ["Imprecision"]  [“Within-study bias”/”indirectness”] |
| **PVI:PVI_BI_mod** | 0 | Some concerns | Low risk | Some concerns | Major concerns | No concerns | No concerns | Low | ["Imprecision"]  [“Within-study bias”/”indirectness”] |
| **PVI:PVI_SUB_mod** | 0 | Some concerns | Low risk | Some concerns | Major concerns | No concerns | No concerns | Low | ["Imprecision"]  [“Within-study bias”/”indirectness”] |
| **PVI:PVI_comb** | 0 | Some concerns | Low risk | Some concerns | Major concerns | No concerns | No concerns | Low | ["Imprecision"]  [“Within-study bias”/”indirectness”] |
| **PVI:PVI_trig** | 0 | Some concerns | Low risk | Some concerns | Major concerns | No concerns | No concerns | Low | ["Imprecision"]  [“Within-study bias”/”indirectness”] |
| **PVI_BI_mod:PVI_EGM** | 0 | Some concerns | Low risk | Some concerns | Major concerns | No concerns | No concerns | Low | ["Imprecision"]  [“Within-study bias”/”indirectness”] |
| **PVI_BI_mod:PVI_GP** | 0 | Some concerns | Low risk | Some concerns | Major concerns | No concerns | No concerns | Low | ["Imprecision"]  [“Within-study bias”/”indirectness”] |
| **PVI_BI_mod:PVI_LAA** | 0 | Some concerns | Low risk | Some concerns | Major concerns | No concerns | No concerns | Low | ["Imprecision"]  [“Within-study bias”/”indirectness”] |
| **PVI_BI_mod:PVI_RDN** | 0 | Some concerns | Low risk | Some concerns | Major concerns | No concerns | No concerns | Low | ["Imprecision"]  [“Within-study bias”/”indirectness”] |
| **PVI_BI_mod:PVI_SUB_mod** | 0 | Some concerns | Low risk | Some concerns | Major concerns | No concerns | No concerns | Low | ["Imprecision"]  [“Within-study bias”/”indirectness”] |
| **PVI_BI_mod:PVI_SVC_lines** | 0 | Some concerns | Low risk | Some concerns | Major concerns | No concerns | No concerns | Low | ["Imprecision"]  [“Within-study bias”/”indirectness”] |
| **PVI_BI_mod:PVI_comb** | 0 | Some concerns | Low risk | Some concerns | Major concerns | No concerns | No concerns | Low | ["Imprecision"]  [“Within-study bias”/”indirectness”] |
| **PVI_BI_mod:PVI_partly** | 0 | Some concerns | Low risk | Some concerns | Major concerns | No concerns | No concerns | Low | ["Imprecision"]  [“Within-study bias”/”indirectness”] |
| **PVI_BI_mod:PVI_posterior_lines** | 0 | Some concerns | Low risk | Some concerns | Major concerns | No concerns | No concerns | Low | ["Imprecision"]  [“Within-study bias”/”indirectness”] |
| **PVI_BI_mod:PVI_step** | 0 | Some concerns | Low risk | Some concerns | Major concerns | No concerns | No concerns | Low | ["Imprecision"]  [“Within-study bias”/”indirectness”] |
| **PVI_BI_mod:PVI_trig** | 0 | Some concerns | Low risk | Some concerns | Major concerns | No concerns | No concerns | Low | ["Imprecision"]  [“Within-study bias”/”indirectness”] |
| **PVI_BI_mod:Singlebox** | 0 | Some concerns | Low risk | Some concerns | Major concerns | No concerns | No concerns | Low | ["Imprecision"]  [“Within-study bias”/”indirectness”] |
| **lines:PVI_BI_mod** | 0 | Some concerns | Low risk | Some concerns | Major concerns | No concerns | No concerns | Low | ["Imprecision"]  [“Within-study bias”/”indirectness”] |
| **PVI_EGM:PVI_GP** | 0 | Some concerns | Low risk | Some concerns | Major concerns | No concerns | No concerns | Low | ["Imprecision"]  [“Within-study bias”/”indirectness”] |
| **PVI_EGM:PVI_LAA** | 0 | Some concerns | Low risk | Some concerns | Major concerns | No concerns | No concerns | Low | ["Imprecision"]  [“Within-study bias”/”indirectness”] |
| **PVI_EGM:PVI_RDN** | 0 | Some concerns | Low risk | Some concerns | Major concerns | No concerns | No concerns | Low | ["Imprecision"]  [“Within-study bias”/”indirectness”] |
| **PVI_EGM:PVI_SUB_mod** | 0 | Some concerns | Low risk | Some concerns | Major concerns | No concerns | No concerns | Low | ["Imprecision"]  [“Within-study bias”/”indirectness”] |
| **PVI_EGM:PVI_SVC_lines** | 0 | Some concerns | Low risk | Some concerns | Major concerns | No concerns | No concerns | Low | ["Imprecision"]  [“Within-study bias”/”indirectness”] |
| **PVI_EGM:PVI_partly** | 0 | Some concerns | Low risk | Some concerns | Major concerns | No concerns | No concerns | Low | ["Imprecision"]  [“Within-study bias”/”indirectness”] |
| **PVI_EGM:PVI_step** | 0 | Some concerns | Low risk | Some concerns | Major concerns | No concerns | No concerns | Low | ["Imprecision"]  [“Within-study bias”/”indirectness”] |
| **PVI_EGM:Singlebox** | 0 | Some concerns | Low risk | Some concerns | Major concerns | No concerns | No concerns | Low | ["Imprecision"]  [“Within-study bias”/”indirectness”] |
| **lines:PVI_EGM** | 0 | Some concerns | Low risk | Some concerns | Major concerns | No concerns | No concerns | Low | ["Imprecision"]  [“Within-study bias”/”indirectness”] |
| **PVI_GP:PVI_LAA** | 0 | Some concerns | Low risk | Some concerns | Major concerns | No concerns | No concerns | Low | ["Imprecision"]  [“Within-study bias”/”indirectness”] |
| **PVI_GP:PVI_RDN** | 0 | Some concerns | Low risk | Some concerns | Major concerns | No concerns | No concerns | Low | ["Imprecision"]  [“Within-study bias”/”indirectness”] |
| **PVI_GP:PVI_SUB_mod** | 0 | Some concerns | Low risk | Some concerns | Major concerns | No concerns | No concerns | Low | ["Imprecision"]  [“Within-study bias”/”indirectness”] |
| **PVI_GP:PVI_SVC_lines** | 0 | Some concerns | Low risk | Some concerns | Major concerns | No concerns | No concerns | Low | ["Imprecision"]  [“Within-study bias”/”indirectness”] |
| **PVI_comb:PVI_GP** | 0 | Some concerns | Low risk | Some concerns | Major concerns | No concerns | No concerns | Low | ["Imprecision"]  [“Within-study bias”/”indirectness”] |
| **PVI_GP:PVI_partly** | 0 | Some concerns | Low risk | Some concerns | Major concerns | No concerns | No concerns | Low | ["Imprecision"]  [“Within-study bias”/”indirectness”] |
| **PVI_GP:PVI_posterior_lines** | 0 | Some concerns | Low risk | Some concerns | Major concerns | No concerns | No concerns | Low | ["Imprecision"]  [“Within-study bias”/”indirectness”] |
| **PVI_GP:PVI_step** | 0 | Some concerns | Low risk | Some concerns | Major concerns | No concerns | No concerns | Low | ["Imprecision"]  [“Within-study bias”/”indirectness”] |
| **PVI_GP:PVI_trig** | 0 | Some concerns | Low risk | Some concerns | Major concerns | No concerns | No concerns | Low | ["Imprecision"]  [“Within-study bias”/”indirectness”] |
| **PVI_GP:Singlebox** | 0 | Some concerns | Low risk | Some concerns | Major concerns | No concerns | No concerns | Low | ["Imprecision"]  [“Within-study bias”/”indirectness”] |
| **lines:PVI_GP** | 0 | Some concerns | Low risk | Some concerns | Major concerns | No concerns | No concerns | Low | ["Imprecision"]  [“Within-study bias”/”indirectness”] |
| **PVI_LAA:PVI_RDN** | 0 | Some concerns | Low risk | Some concerns | Major concerns | No concerns | No concerns | Low | ["Imprecision"]  [“Within-study bias”/”indirectness”] |
| **PVI_LAA:PVI_SUB_mod** | 0 | Some concerns | Low risk | Some concerns | Major concerns | No concerns | No concerns | Low | ["Imprecision"]  [“Within-study bias”/”indirectness”] |
| **PVI_LAA:PVI_SVC_lines** | 0 | Some concerns | Low risk | Some concerns | Major concerns | No concerns | No concerns | Low | ["Imprecision"]  [“Within-study bias”/”indirectness”] |
| **PVI_comb:PVI_LAA** | 0 | Some concerns | Low risk | Some concerns | Major concerns | No concerns | No concerns | Low | ["Imprecision"]  [“Within-study bias”/”indirectness”] |
| **PVI_LAA:PVI_lines** | 0 | Some concerns | Low risk | Some concerns | Major concerns | No concerns | No concerns | Low | ["Imprecision"]  [“Within-study bias”/”indirectness”] |
| **PVI_LAA:PVI_partly** | 0 | Some concerns | Low risk | Some concerns | Major concerns | No concerns | No concerns | Low | ["Imprecision"]  [“Within-study bias”/”indirectness”] |
| **PVI_LAA:PVI_posterior_lines** | 0 | Some concerns | Low risk | Some concerns | Major concerns | No concerns | No concerns | Low | ["Imprecision"]  [“Within-study bias”/”indirectness”] |
| **PVI_LAA:PVI_step** | 0 | Some concerns | Low risk | Some concerns | Major concerns | No concerns | No concerns | Low | ["Imprecision"]  [“Within-study bias”/”indirectness”] |
| **PVI_LAA:PVI_trig** | 0 | Some concerns | Low risk | Some concerns | Major concerns | No concerns | No concerns | Low | ["Imprecision"]  [“Within-study bias”/”indirectness”] |
| **PVI_LAA:Singlebox** | 0 | Some concerns | Low risk | Some concerns | Major concerns | No concerns | No concerns | Low | ["Imprecision"]  [“Within-study bias”/”indirectness”] |
| **lines:PVI_LAA** | 0 | Some concerns | Low risk | Some concerns | Major concerns | No concerns | No concerns | Low | ["Imprecision"]  [“Within-study bias”/”indirectness”] |
| **PVI_RDN:PVI_SUB_mod** | 0 | Some concerns | Low risk | Some concerns | Major concerns | No concerns | No concerns | Low | ["Imprecision"]  [“Within-study bias”/”indirectness”] |
| **PVI_RDN:PVI_SVC_lines** | 0 | Some concerns | Low risk | Major concerns | Major concerns | No concerns | No concerns | Low | ["Indirectness"]["Imprecision"] |
| **PVI_comb:PVI_RDN** | 0 | Some concerns | Low risk | Some concerns | Major concerns | No concerns | No concerns | Low | ["Imprecision"]  [“Within-study bias”/”indirectness”] |
| **PVI_lines:PVI_RDN** | 0 | Some concerns | Low risk | Some concerns | Major concerns | No concerns | No concerns | Low | ["Imprecision"]  [“Within-study bias”/”indirectness”] |
| **PVI_partly:PVI_RDN** | 0 | Some concerns | Low risk | Some concerns | Major concerns | No concerns | No concerns | Low | ["Imprecision"]  [“Within-study bias”/”indirectness”] |
| **PVI_posterior_lines:PVI_RDN** | 0 | Some concerns | Low risk | Some concerns | Major concerns | No concerns | No concerns | Low | ["Imprecision"]  [“Within-study bias”/”indirectness”] |
| **PVI_RDN:PVI_step** | 0 | Some concerns | Low risk | Some concerns | Major concerns | No concerns | No concerns | Low | ["Imprecision"]  [“Within-study bias”/”indirectness”] |
| **PVI_RDN:PVI_trig** | 0 | Some concerns | Low risk | Some concerns | Major concerns | No concerns | No concerns | Low | ["Imprecision"]  [“Within-study bias”/”indirectness”] |
| **PVI_RDN:Singlebox** | 0 | Some concerns | Low risk | Some concerns | Major concerns | No concerns | No concerns | Low | ["Imprecision"]  [“Within-study bias”/”indirectness”] |
| **lines:PVI_RDN** | 0 | Some concerns | Low risk | Some concerns | Major concerns | No concerns | No concerns | Low | ["Imprecision"]  [“Within-study bias”/”indirectness”] |
| **PVI_SUB_mod:PVI_SVC_lines** | 0 | Some concerns | Low risk | Some concerns | Major concerns | No concerns | No concerns | Low | ["Imprecision"]  [“Within-study bias”/”indirectness”] |
| **PVI_comb:PVI_SUB_mod** | 0 | Some concerns | Low risk | Some concerns | Major concerns | No concerns | No concerns | Low | ["Imprecision"]  [“Within-study bias”/”indirectness”] |
| **PVI_lines:PVI_SUB_mod** | 0 | Some concerns | Low risk | Some concerns | Major concerns | No concerns | No concerns | Low | ["Imprecision"]  [“Within-study bias”/”indirectness”] |
| **PVI_partly:PVI_SUB_mod** | 0 | Some concerns | Low risk | Some concerns | Major concerns | No concerns | No concerns | Low | ["Imprecision"]  [“Within-study bias”/”indirectness”] |
| **PVI_SUB_mod:PVI_trig** | 0 | Some concerns | Low risk | Some concerns | Major concerns | No concerns | No concerns | Low | ["Imprecision"]  [“Within-study bias”/”indirectness”] |
| **PVI_SUB_mod:Singlebox** | 0 | Some concerns | Low risk | Some concerns | Major concerns | No concerns | No concerns | Low | ["Imprecision"]  [“Within-study bias”/”indirectness”] |
| **lines:PVI_SUB_mod** | 0 | Some concerns | Low risk | Some concerns | Major concerns | No concerns | No concerns | Low | ["Imprecision"]  [“Within-study bias”/”indirectness”] |
| **PVI_lines:PVI_SVC_lines** | 0 | Some concerns | Low risk | Some concerns | Major concerns | No concerns | No concerns | Low | ["Imprecision"]  [“Within-study bias”/”indirectness”] |
| **PVI_partly:PVI_SVC_lines** | 0 | Some concerns | Low risk | Some concerns | Major concerns | No concerns | No concerns | Low | ["Imprecision"]  [“Within-study bias”/”indirectness”] |
| **PVI_posterior_lines:PVI_SVC_lines** | 0 | Some concerns | Low risk | Some concerns | Major concerns | No concerns | No concerns | Low | ["Imprecision"]  [“Within-study bias”/”indirectness”] |
| **PVI_step:PVI_SVC_lines** | 0 | Some concerns | Low risk | Some concerns | Major concerns | No concerns | No concerns | Low | ["Imprecision"]  [“Within-study bias”/”indirectness”] |
| **PVI_SVC_lines:PVI_trig** | 0 | Some concerns | Low risk | Some concerns | Major concerns | No concerns | No concerns | Low | ["Imprecision"]  [“Within-study bias”/”indirectness”] |
| **PVI_SVC_lines:Singlebox** | 0 | Some concerns | Low risk | Some concerns | Major concerns | No concerns | No concerns | Low | ["Imprecision"]  [“Within-study bias”/”indirectness”] |
| **lines:PVI_SVC_lines** | 0 | Some concerns | Low risk | Some concerns | Major concerns | No concerns | No concerns | Low | ["Imprecision"]  [“Within-study bias”/”indirectness”] |
| **PVI_comb:PVI_lines** | 0 | Some concerns | Low risk | Some concerns | Major concerns | No concerns | No concerns | Low | ["Imprecision"]  [“Within-study bias”/”indirectness”] |
| **PVI_comb:PVI_partly** | 0 | Some concerns | Low risk | Some concerns | Major concerns | No concerns | No concerns | Low | ["Imprecision"]  [“Within-study bias”/”indirectness”] |
| **PVI_comb:PVI_step** | 0 | Some concerns | Low risk | Some concerns | Major concerns | No concerns | No concerns | Low | ["Imprecision"]  [“Within-study bias”/”indirectness”] |
| **PVI_comb:PVI_trig** | 0 | Some concerns | Low risk | Some concerns | Major concerns | No concerns | No concerns | Low | ["Imprecision"]  [“Within-study bias”/”indirectness”] |
| **PVI_comb:Singlebox** | 0 | Some concerns | Low risk | Some concerns | Major concerns | No concerns | No concerns | Low | ["Imprecision"]  [“Within-study bias”/”indirectness”] |
| **lines:PVI_comb** | 0 | Some concerns | Low risk | Some concerns | Major concerns | No concerns | No concerns | Low | ["Imprecision"]  [“Within-study bias”/”indirectness”] |
| **PVI_lines:PVI_partly** | 0 | Some concerns | Low risk | Some concerns | Major concerns | No concerns | No concerns | Low | ["Imprecision"]  [“Within-study bias”/”indirectness”] |
| **PVI_lines:Singlebox** | 0 | Some concerns | Low risk | Some concerns | Major concerns | No concerns | No concerns | Low | ["Imprecision"]  [“Within-study bias”/”indirectness”] |
| **lines:PVI_lines** | 0 | Some concerns | Low risk | Some concerns | Major concerns | No concerns | No concerns | Low | ["Imprecision"]  [“Within-study bias”/”indirectness”] |
| **PVI_partly:PVI_posterior_lines** | 0 | Some concerns | Low risk | Some concerns | Major concerns | No concerns | No concerns | Low | ["Imprecision"]  [“Within-study bias”/”indirectness”] |
| **PVI_partly:PVI_step** | 0 | Some concerns | Low risk | Some concerns | Major concerns | No concerns | No concerns | Low | ["Imprecision"]  [“Within-study bias”/”indirectness”] |
| **PVI_partly:PVI_trig** | 0 | Some concerns | Low risk | Some concerns | Major concerns | No concerns | No concerns | Low | ["Imprecision"]  [“Within-study bias”/”indirectness”] |
| **PVI_partly:Singlebox** | 0 | Some concerns | Low risk | Some concerns | Major concerns | No concerns | No concerns | Low | ["Imprecision"]  [“Within-study bias”/”indirectness”] |
| **lines:PVI_partly** | 0 | Some concerns | Low risk | Some concerns | Major concerns | No concerns | No concerns | Low | ["Imprecision"]  [“Within-study bias”/”indirectness”] |
| **PVI_posterior_lines:PVI_step** | 0 | Some concerns | Low risk | Some concerns | Major concerns | No concerns | No concerns | Low | ["Imprecision"]  [“Within-study bias”/”indirectness”] |
| **PVI_posterior_lines:PVI_trig** | 0 | Some concerns | Low risk | Some concerns | Major concerns | No concerns | No concerns | Low | ["Imprecision"]  [“Within-study bias”/”indirectness”] |
| **PVI_posterior_lines:Singlebox** | 0 | Some concerns | Low risk | Some concerns | Major concerns | No concerns | No concerns | Low | ["Imprecision"]  [“Within-study bias”/”indirectness”] |
| **lines:PVI_posterior_lines** | 0 | Some concerns | Low risk | Some concerns | Major concerns | No concerns | No concerns | Low | ["Imprecision"]  [“Within-study bias”/”indirectness”] |
| **PVI_step:Singlebox** | 0 | Some concerns | Low risk | Some concerns | Major concerns | No concerns | No concerns | Low | ["Imprecision"]  [“Within-study bias”/”indirectness”] |
| **lines:PVI_step** | 0 | Some concerns | Low risk | Some concerns | Major concerns | No concerns | No concerns | Low | ["Imprecision"]  [“Within-study bias”/”indirectness”] |
| **PVI_trig:Singlebox** | 0 | Some concerns | Low risk | Some concerns | Major concerns | No concerns | No concerns | Low | ["Imprecision"]  [“Within-study bias”/”indirectness”] |
| **lines:PVI_trig** | 0 | Some concerns | Low risk | Some concerns | Major concerns | No concerns | No concerns | Low | ["Imprecision"]  [“Within-study bias”/”indirectness”] |
| **lines:Singlebox** | 0 | Some concerns | Low risk | Some concerns | Major concerns | No concerns | No concerns | Low | ["Imprecision"]  [“Within-study bias”/”indirectness”] |
